# Plausible Pnicogen Bonding of *epi-*Cinchonidine as a Chiral Scaffold in Catalysis

**DOI:** 10.3389/fchem.2021.669515

**Published:** 2021-07-06

**Authors:** Zakir Ullah, Kang Kim, Arramshetti Venkanna, Hye su Kim, Moon Il Kim, Mi-hyun Kim

**Affiliations:** ^1^ Department of Pharmacy, College of Pharmacy, Gachon Institute of Pharmaceutical Science, Gachon University, Incheon, South Korea; ^2^ Department of Chemistry, Korea Advanced Institute of Science and Technology, Daejeon, South Korea; ^3^ Department of BioNano Technology, Gachon University, Seongnam, South Korea

**Keywords:** pnicogen bonding, DFT calculation, enantiotopic face, HOMO–LUMO, UV-Vis spectroscopy

## Abstract

As a non-covalent interaction of a chiral scaffold in catalysis, pnicogen bonding of *epi-*cinchonidine (*epi-CD*), a cinchona alkaloid, was simulated to consider whether the interaction can have the potential controlling enantiotopic face like hydrogen bonding. Among five reactive functional groups in *epi-CD*, two stable complexes of the hydroxyl group (X-epi-CD1) at C_17_ and of the quinoline ring (X-epi-CD2) at N_16_ with pnictide family analytes [X = substituted phosphine (PX), i.e., F, Br, Cl, CF_3_, CN, HO, NO_2_, and CH_3_, and pnictide family analytes, i.e., PBr_3_, BiI_3_, SbI_3_, and AsI_3_] were predicted with intermolecular interaction energies, charge transfer (Q_Mulliken_ and Q_NBO_), and band gap energies of HOMO–LUMO (Eg) at the B3LYP/6-31G(d,p) level of density functional theory. It was found that the dominant site of pnicogen bonding in epi-CD is the quinoline ring (N_16_ atom) rather than the hydroxyl group (O_36_ atom). In addition, the UV-Vis spectra of the complex were calculated by time-dependent density functional theory (TD-DFT) at the B3LYP/6-31+G(d,p) level and compared with experimental measurements. Through these calculations, two intermolecular interactions (H-bond vs. pnicogen bond) of *epi-CD* were compared.

## Introduction

**FIGURE 1:**
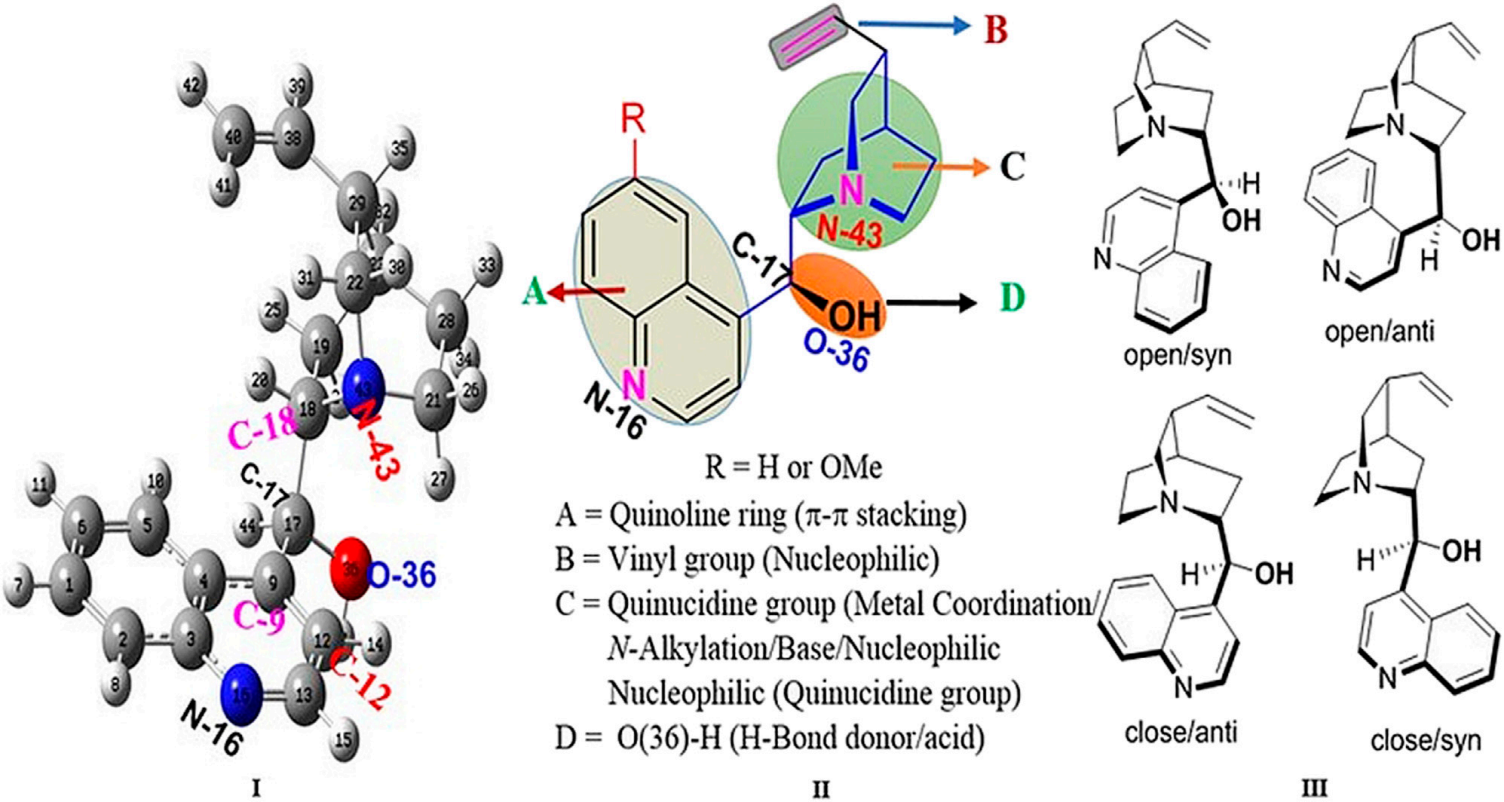
Reactive sites of cinchona alkaloids and the representative conformations of epi-cinchonidine (**right:** syn/anti, open/close; **left:** optimized geometry with the atomic number).

Cinchona alkaloids have played an important role as privileged chiral sources in the history of chemistry due to their diverse chiral skeletons and tunable reactions (like chiral ligands in Sharpless asymmetric dihydroxylation; Maeda et al., 2011; Tian et al., 2004; Szollosi et al., 2011; Sim et al., 2015; Marcelli and Hiemstra, 2010). In addition, they were clinically used as antimalarial or anti-arrhythmic agents (Gurung and De, 2017; Yardley et al., 1971; de Villiers et al., 2012; Vandekerckhove and D’hooghe, 2015; Karle and Bhattacharjee, 1999; Wesche et al., 1990). Recently, cinchona alkaloids have been screened as an anti-diabetic agent (Ezekwesili et al., 2012) and used as the most powerful chiral template for designing new organic catalysts (e.g., bifunctional catalyst, phase-transfer catalyst) (Dalko and Moisan, 2001; Jones, 2001; Lygo and Wainwright, 1997). Reactive sites in cinchona alkaloids and their derivatives were widely studied (Ullah and Itsuno, 2017; Li et al., 2004; Wang et al., 2007; Yeboah et al., 2011; Luo et al., 2009). The highly basic and bulky nitrogen atom of quinuclidine is able to bind with an electrophile or metal to produce a stereotopic face (Figure 1) (Song, 2009a). The vicinal aminoalcohol in cinchona alkaloids has the capability to associate the proximal hydroxyl group at C_17_ (Lewis acid) with the nitrogen atom of quinuclidine (Lewis base). The methoxy group in the quinolone ring of quinines and quinidines can be converted into the free phenolic −OH group as an H-donor. Similarly, quinoline exhibits π–π stacking, and its vinyl group can act as a nucleophile. However, the delicate differences in the reactivity of their five functional groups have not been sufficiently investigated when compared with intensive use of cinchona alkaloids. In particular, despite their potential controlling asymmetric reactions through the non-covalent interaction (NCI) (Manna and Mugesh, 2012; Pal et al., 2016; Wheeler et al., 2016; Benz et al., 2017; Breugst et al., 2017; Li and Hong, 2018), the scope and limitation of the NCIs in these five functional groups has not been properly studied. Rather than the study, conformational investigations of cinchona are reported (Yanuka et al., 1981; Dijkstra et al., 1989a; Dijkstra et al., 1989b; Caner et al., 2003; Prakash et al., 2011; Wang et al., 2014). Exceptionally, an oxyanion hole between the hydroxyl group at C_17_ and the nitrogen atom of quinuclidine (N_43_) was proposed (Bürgi and Baiker, 1998), and a density functional theory (DFT) study of quinine-catalyzed aza-Henry reaction explained the mechanism through the hydrogen bonding interaction (Xue et al., 2016).

**GRAPHICAL ABSTRACT:**
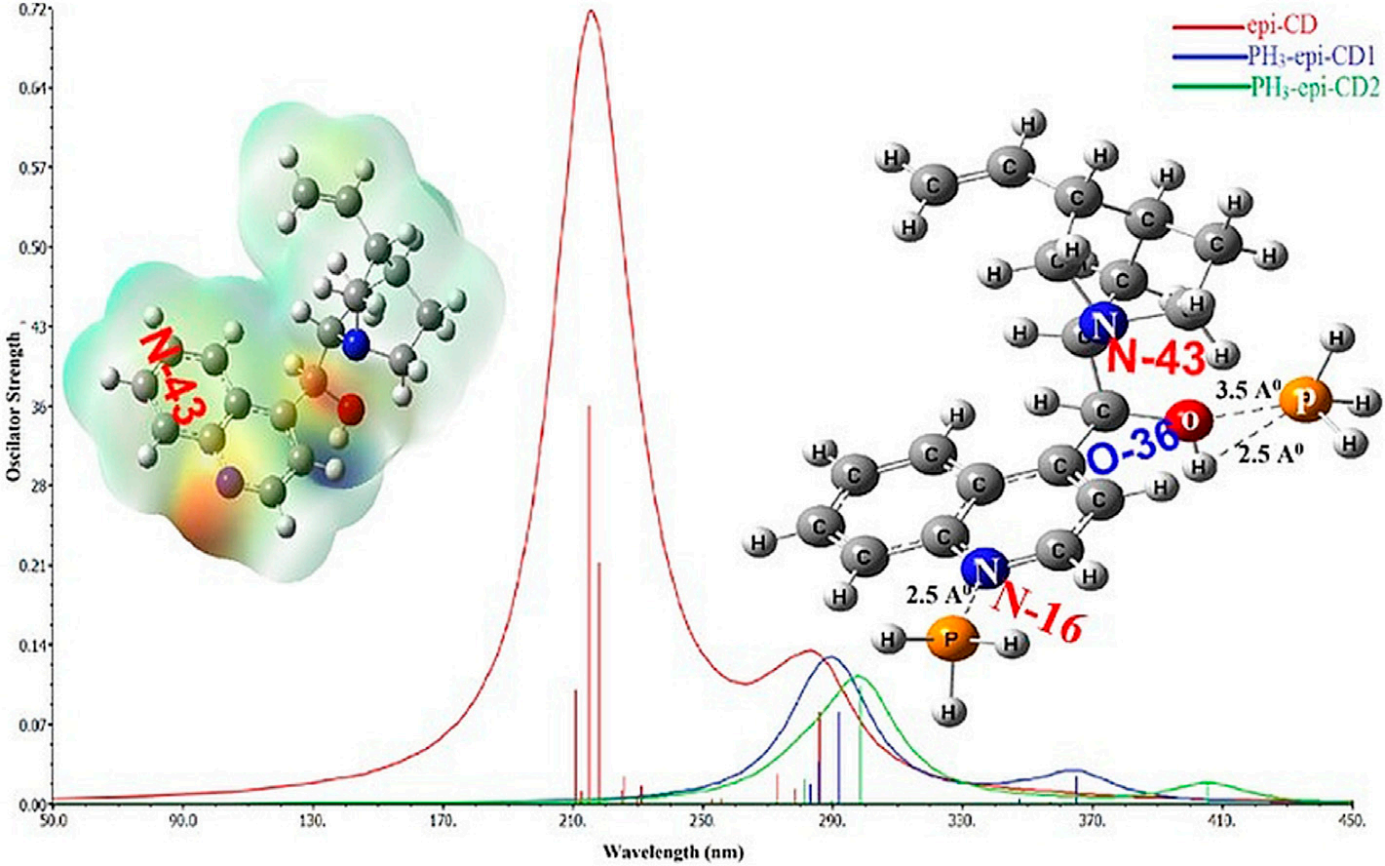


NCIs are very crucial phenomena happening at the atomic level so that hydrogen bonding and halogen bonding among them have been strongly used for molecular design in various fields (e.g., drug design, catalyst design, porous material architecture design) (Hiemstra and Wynberg, 1981; Shi and Xu, 2002; Li et al., 2005a; Li et al., 2005b; Akiyama et al., 2006; Taylor and Jacobsen, 2006; Doyle and Jacobsen, 2007; Yang and Wong, 2013; Grayson and Houk, 2016a; Cavallo et al., 2016; Qian et al., 2017; Heinen et al., 2018). Recently, new types of non-covalent bonds such as carbon bonding (Escudero-Adán et al., 2015; Hatakeyama et al., 2015), pnicogen bonding (Scheiner, 2011a; Scheiner, 2011b; Scheiner and Adhikari, 2011; Sánchez-Sanz et al., 2012; Alkorta and Elguero, 2013; Alkorta et al., 2013; Scheiner, 2013a; Scheiner, 2013b; Alkorta and Elguero, 2014; Del Bene et al., 2014; Sánchez-Sanz et al., 2015; Scheiner, 2015; Del Bene et al., 2017), chalcogen bonding (Fanfrlík et al., 2014; George et al., 2014), and aerogen bonding (Bauz and Frontera, 2015; Esrafili and Vessally, 2016; Gao et al., 2016; Esrafili and Qasemsolb, 2017) have been investigated, and properties of the bonds have also been applied to catalysis (Manna and Mugesh, 2012; Pal et al., 2016; Wheeler et al., 2016; Benz et al., 2017; Breugst et al., 2017; Li and Hong, 2018). Among the applicable properties, σ-hole, a positive electrostatic region, is the most outstanding. The σ-hole is the interaction between a covalently bonded atom of groups 14–18 as a Lewis acid and a lone pair present in a Lewis base or an anion (Politzer et al., 2013). The formation of a σ-hole bond results from the existence of an electron-deficient region (σ-hole) in the outer lobe of the half-filled p orbital that is involved in the covalent bond. To our knowledge, such new types of non-covalent bonds have not been applied yet to directly control the enantiotopic face of a chiral organocatalyst. If such bonds can be designed onto a chiral scaffold (or catalyst) and can play key interactions in controlling the stereoelectronic environment, the interactions inducing chiral bias of the product can be state-of-the-art based on more diverse available elements and bond strength of a broader range rather than the current interactions (hydrogen bonding, ionic interaction, etc.) in the catalytic enantioselective transformation area. Until today, promising interactions have been conceptually established, and some successful studies were reported through computational study and experimental proofs (Jungbauer and Huber, 2015; Benz et al., 2016; Bulfield and Huber, 2016; Benz et al., 2017; Gliese et al., 2017; Schmauck and Breugst, 2017). However, reported studies are on racemic reactions (Athawale and Manjrekar, 2001; Kacprzak and Gawronski, 2001; Jha and Joshi, 2002; Gaunt et al., 2007; Vakulya et al., 2008; Wang et al., 2011; De Fusco et al., 2012; Pham et al., 2013; Jusseau et al., 2014; Chen et al., 2015; Nath et al., 2015; Scorzelli et al., 2015; Singh and Yeboah, 2016; Viveros-Ceballos et al., 2016; Auria-Luna et al., 2017; Gliese et al., 2017; Guo and Wong, 2017; Szollosi, 2017; Fulton et al., 2018; Li and Hong, 2018; Zhang et al., 2018), or new types of bonds cannot contribute to enantioselectivity (Athawale and Manjrekar, 2001; Kacprzak and Gawronski, 2001; Jha and Joshi, 2002; Gaunt et al., 2007; Vakulya et al., 2008; Wang et al., 2011; De Fusco et al., 2012; Pham et al., 2013; Jusseau et al., 2014; Chen et al., 2015; Nath et al., 2015; Scorzelli et al., 2015; Benz et al., 2016; Bulfield and Huber, 2016; Singh and Yeboah, 2016; Viveros-Ceballos et al., 2016; Auria-Luna et al., 2017; Guo and Wong, 2017; Szollosi, 2017; Fulton et al., 2018; Zhang et al., 2018). Therefore, for future research, the current research background motivated us in applying the new types of NCIs (groups 14–18) into a cinchona alkaloid as a privileged chiral scaffold of the organocatalyst or an organocatalyst itself.

In this paper, we studied the possibility of pnicogen bonding between a Lewis base of *epi*-cinchonidine (*epi-CD*), a type of cinchona alkaloid, and covalently bonded P, As, Sb, and Bi of the pnictide family as a Lewis acid. To our knowledge, there are no reports on pnicogen bonding in any chiral scaffold (among chiral ligands, chiral auxiliaries, and chiral catalysts) like cinchona alkaloids so that a theoretical study on pnicogen bonding can be a guidance for reaction designs in the field of catalysis and asymmetric synthesis. To compare hydrogen bonding with pnicogen bonding, *epi-CD* complexes interacting with methacrylic acid (MA) were simulated to locate the hydrogen bonding in *epi-CD*. The simulation of pnicogen bonding in *epi-CD* was designed with PH_3_ and substituted phosphane derivatives having F, Br, Cl, CF_3_, CN, HO, NO_2_, and CH_3_ as substituents to describe substituent effects on pnicogen bonding.

## Computational Methodology

DFT calculations were performed using a hybrid functional [Becke 3-parameter (exchange), Lee, Yang, and Parr with both the local and non-local correlations, B3LYP] (Becke, 1988; Becke, 1993) with well-accepted basis sets: 6-31G(d,p) and 6-31G+(d,p) (Bürgi et al., 2002). The DGDZVP basis set for As and Sb atoms and SDD basis set for Bi atoms were used. All calculations were performed at the default temperature and pressure (298.15 K and 1.00 atm). All calculations were performed using Gaussian 09 (Frisch et al., 2009), and the results were visualized with the GaussView, Gabedit (Allouche, 2011), and GaussSum (O’boyle et al., 2008) computer programs. The term “epi-CD” was used as an abbreviated name for epi-cinchonidine, and epi-CD-X was used for its complexes. epi-CD and its epi-CD-X complexes (where X is an analyte) were neutral with singlet spins (charge = 0 and singlet state). Vibrational frequency calculations were used to confirm that the optimized structures were true minimum on the potential energy surface, as characterized by the absence of imaginary vibrational frequencies. The intermolecular interaction energies and excited-state properties (e.g., UV-Vis spectra) (Salzner, 2008), Q_Mulliken_ and Q_NBO_, charge analysis, and band gap energies (Eg) were calculated at the above-mentioned level of theory. We simulated Q_Mulliken_ and Q_NBO_ charge analyses with different types of basis sets and concluded that the properties were basis set dependent (Ullah et al., 2013). The band gap energies were estimated from the energy differences for the lowest unoccupied molecular orbital (LUMO) and highest occupied molecular orbital (HOMO), where the negative of the HOMO was the ionization potential (IP) and the energy of the LUMO was estimated from the electron affinity (EA). Eq. 1 was used to simulate the binding energies of the various optimized structures. The counterpoise method corrected this energy, which was based on Eq. 2 (Boys and Bernardi, 1970):

$$\Delta {\text{E}}_{\mathrm{int}}={\text{E}}_{\left(\text{Product}\right)}-\left({\text{E}}_{\left(\text{Reactant}1\right)}+{\text{E}}_{\left(\text{Reactant}2\right)}\right),$$ (1)

$$\Delta {\text{E}}_{\text{int,CP}}=\Delta {\text{E}}_{\text{int}}-{\text{E}}_{\text{BSSE}},$$ (2)

where ΔE_int_ is the total binding energy of the optimized epi-CD interacting with different analytes. E_(Reactant1)_ is the total energy of isolated epi-CD and E_(Reactant2)_ is that of analytes, while E_(Product)_ is the total energy of a particular epi-CD-X complex. E_BSSE_ is the basis set superposition error energy of epi-CD-X, and ΔE_int,CP_ is the geometrical counterpoise–corrected interaction energy of these complexes. The binding energies of Eqs 1, 2 are related to the relaxed structures with the minimum amount of energy. The B3LYPgCP-D3/6-31G (Brandenburg et al., 2013) energy corrections are also made from Grimme’s web server (http://wwwtc.thch.uni-bonn.de/) (Kruse et al., 2012). ΔE_gCP-D3_ is the geometrical counterpoise–corrected energy with D3 corrections, whereas E_(Reactant1)gCP-D3_ is used for epi-CD and the analytes and E_(Product)gCP-D3_ is the binding energies of the epi-CD complex. In addition, geometry optimization of one example considering the dispersion term also was conducted using B3LYP-D3 with 6-31G(d,p) (Kruse et al., 2012; Brandenburg et al., 2013), and then two geometries resulting from B3LYP and B3LYP-D3 were compared to confirm the acceptability of our chosen method, B3LYP/6-31G. The NCI energy can be estimated as negative, i.e., more negative energy relates to high stability, and vice versa.

## Synthetic Description and Spectra Measurement

**FIGURE 2:**
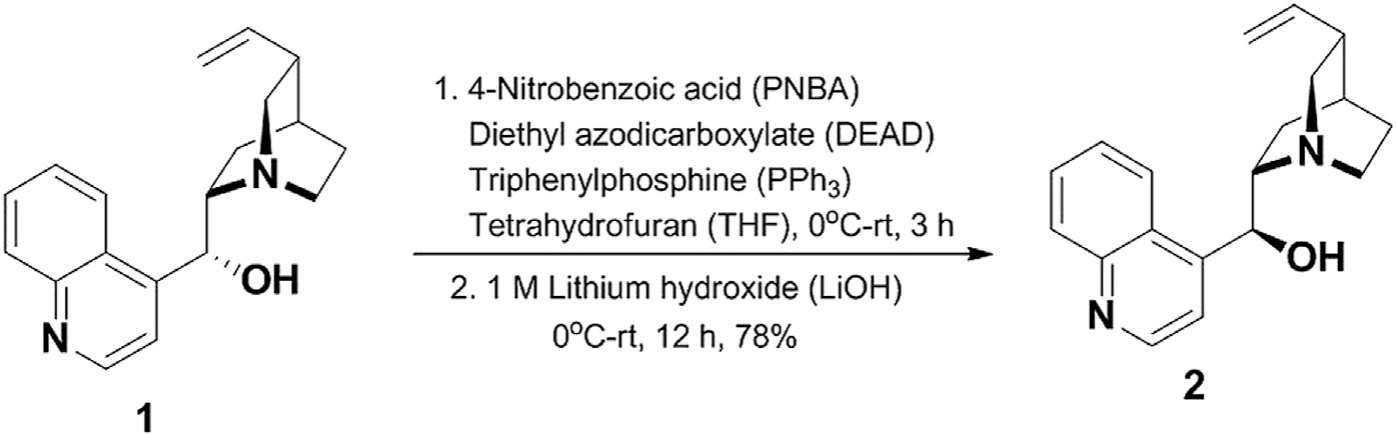
Synthetic route of *epi*-cinchonidine.

For explanation of pnicogen bonding of *epi*-cinchonidine, we synthesized *epi*-cinchonidine (**2**) from cheaply available cinchonidine (**1**) as mentioned in Figure 2. Cinchonidine (**1**) was subjected to one-pot Mitsunobu inversion followed by saponification with 4-nitrobenzoic acid (PNBA)/diethyl azodicarboxylate (DEAD)/triphenylphosphine (PPh_3_) and 1 M LiOH (lithium hydroxide) to afford diastereomerically pure *epi*-cinchonidine (**2**) in 78% yield (Sidorowicz and Skarżewski, 2011). All spectra data are given in the supplementary material.

For the IR and UV-Vis absorption spectra, 0.5 mg/ml epi-CD solution in three different solvents [CHCl3, EtOH, and DW (distilled water)] was prepared according to solubility of analytes (Qin et al., 2009). Analyte solutions were also prepared to the same concentration (1.7 mM). Without additional treatment, the UV-Vis absorption spectra of sole epi-CD solution were measured after the blank test (solvent). After mixing two solutions (analyte: epi-CD = 1 to 1), the equilibrium between the complex and the free form waited, and then UV-Vis absorption spectra were recorded on a NanoDrop 1000 UV-Vis spectrophotometer. The experimental result could be integrated for the comparison with the predicted result.

## Results and Discussion

**Geometry Selection of epi-CD.** In this study, epi-cinchonidine (epi-CD) was chosen among available four cinchona alkaloids due to conformational rigidity (Yanuka et al., 1981; Dijkstra et al., 1989a; Dijkstra et al., 1989b; Caner et al., 2003; Prakash et al., 2011; Wang et al., 2014). For economic geometry selection among an enormous number of conformers, two torsional angles τ_1_ (C_12_−C_9_−C_17_−C_18_) and τ_2_ (C_9_−C_17_−C_18_−N_43_) were used as criteria of conformer generation based on previous reports on cinchona alkaloids (Bürgi and Baiker, 1998). The τ_1_ rotation decides the interconversion between closed and open conformations on whether the quinuclidine nitrogen atom of cinchona is close to ring A of quinolone or not. The *τ*_2_ rotation determines *syn* and *anti* conformations according to the relative position of the hydroxy group (OH) aligning with ring B of quinoline. Even though more stable conformation can change according to solvents and substituents, every conformer used in this study was in the *anti* opened state for maximizing the space which analytes can be close to. The prominent optimized structural parameters (bond length, bond angle, and dihedral angle) in the geometry of epi-CD were calculated at DFT-B3LYP/6-31G(d,p) and 6-311G levels of theory, and the results are compared with the Cambridge Crystallographic Data Centre (CCDC) data of the epi-cinchonidine derivative (Martin and Zipse, 2005; Klare et al., 2014) in Table 1.

**TABLE 1:** Optimized geometric parameters of *epi-CD*.

Geometric parameter	Experimental X-ray^a^	B3LYP/6-31G(d,p)	B3LYP/6-311G
**Bond length(Å)**			
O_36_-H_37_, C_17_-O_36_, C_17_-H_44_	NA, NA, 0.999	0.966, 1.427, 1.101	0.962, 1.429, 1.098
C_17_-C_18_, C_17_-C_9_, C_18_-N_43_	1.535, 1.317, 1.473	1.545, 1.522, 1.478	1.543, 1.520, 1.477
C_6_-C_1_, C_4_-C_3_, C_22_-C_29_	1.413, 1.427, 1.600	1.414, 1.435, 1.565	1.412, 1.432, 1.565
**Bond angle(Å)**			
C_17_-O_36_-H_37_, H_44_-C_17_-O_36_	NA, NA	107.66, 108.56	107.95, 108.38
C_3_-N_16_-C_13_, C_18_-N_43_-C_21_	117.148, 108.047	117.15, 112.13	117.29, 112.24
**Dihedral angle(Å)**			
H_37_-O_36_-C_17_-C_9_, C_17_-C_18_-N_43_-C_21_	NA, NA	58.008,80.108	59.79, 80.54
H_15_-C_13_-N_16_-C_3_, C_2_-C_3_-N_16_-C_13_	179.439, 180.000	179.808, 179.820	179.68, 179.85

a The experimental values were acquired from CCDC data of the epi-cinchonidine derivative (CCDC ID: 958721). When a corresponding value is not available in the compound, the value is presented as “NA (not available).”

**Charge Analysis and Molecular Electrostatic Potential of *epi-CD.*** Before the reactive site simulation of epi-CD, both Mulliken charge and natural bonding orbital (NBO) charge on the optimized geometry of epi-CD were calculated. The highest negatively charged atom (the most nucleophilic) commonly present was O_36_ in both charges (NBO: −0.76 e^−^, Mulliken: −0.55 e^−^). N_16_, C_40_, N_43_, and C_28_ were also negatively charged, but the reactivity order was not identical (Mulliken: N_16_ > N_43_ >> C_40_ > C_28_, NBO: N_43_ > C_28_ > C_40_ > N_16_) in Supplementary Tables S1, S2. The molecular electrostatic potential (MEP) of epi-CD predicted the nature of the electrophilic and nucleophilic reactions of the molecule to present the charge density, delocalization, and site of the molecular chemical reactivity. Based on known reports depicting five reactive functional groups of cinchona alkaloids (Song, 2009b), the quinoline ring and hydroxyl group (O_36_ atom), tertiary nitrogen (N_43,_ N_16_), and olefin region (C_40_) were expected to show high electron density. In particular, the MEP map presented the quinoline ring as the highest charge density and the region around O_36_ as the highest electron density.

**FIGURE 3:**
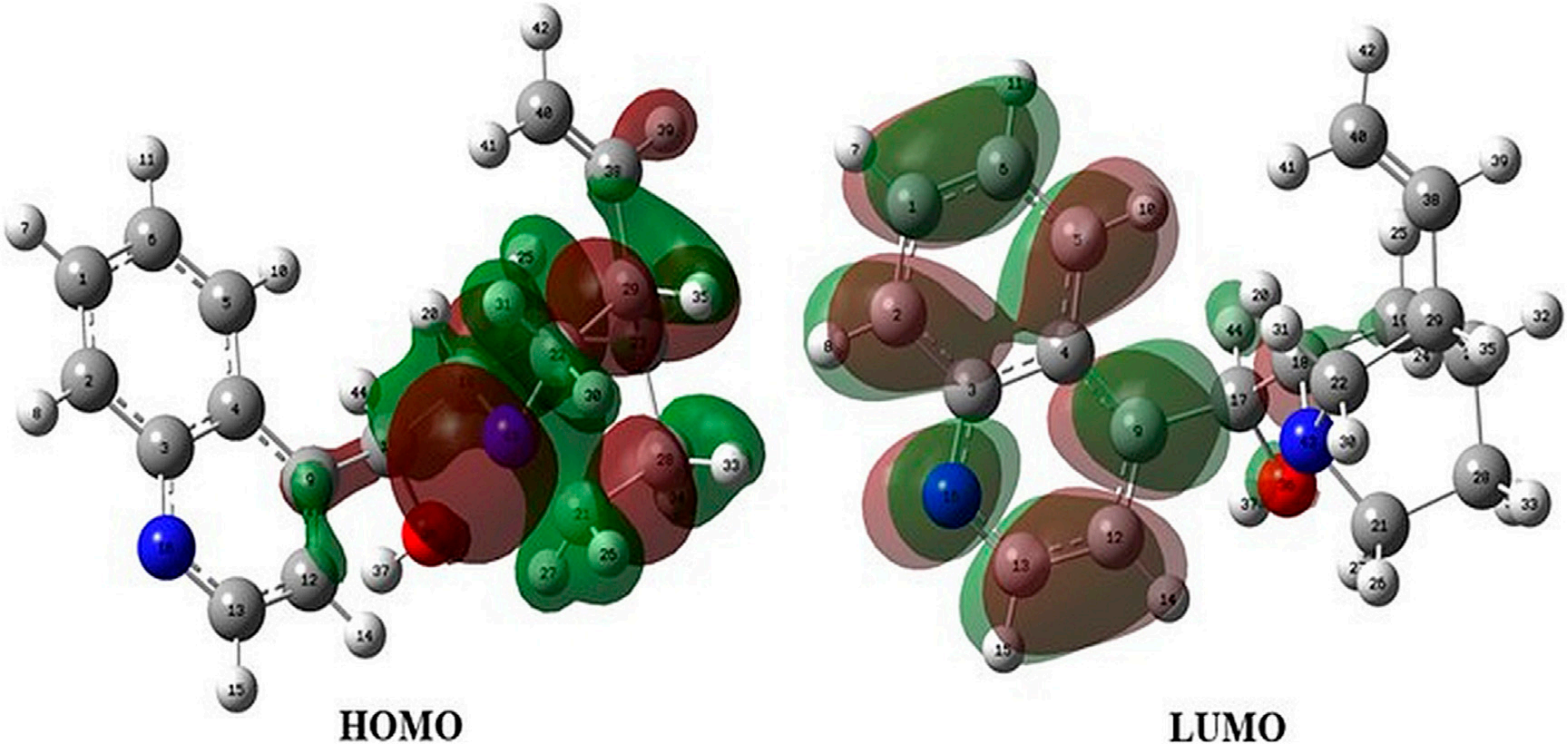
HOMO and LUMO of epi-cinchonidine.

Orbital energies of the HOMO and the lowest LUMO were also calculated for electric and optical properties like the UV-Vis spectra at the B3LYP/6-31G(d,p) level. The energy gap between the HOMO and the LUMO, indicating the molecular chemical stability and electrical transport property, was 4.20 eV, and the value as the standard was compared with the HOMO–LUMO energy gap of each epi-CD-X complex. In contrast to the MEP map, the frontier molecular orbital analysis could not propose the reactivity of the olefin (C_40_), and the electron density of the HOMO was distributed on the right side of epi-CD, especially on the C_17_ hydroxyl group (O_36_), quinuclidine (N_43_), and some C–C bonds (Figure 3). In the LUMO, the quinoline ring (N_16_) and the hydroxyl group (O_36_) showed prominent electron density. Our experimental reaction with excessive electrophile, methyl iodide, under aqueous basic condition also showed the dialkylated product at only N_43_ and N_16_ positions (Supplementary Figure S3).

**Infrared Spectral Characteristics and UV-Vis Spectral Analysis of *epi-CD.*** According to the non-linear molecular vibrational mode formula, epi-CD is expected to have 126 normal modes of vibrations under C1 symmetry. The simulated infrared (IR) spectrum of epi-CD is plotted and compared with the experimental spectrum in Supplementary Figure S4. Comparison of simulated spectra with experimental spectra enabled assignment of the vibrational modes to experimental vibrational bands. Among 126 modes, selected major band peaks and their approximate assignments are listed in Table 2. In particular, the functional group region of the simulated IR spectrum contained seven peaks comprising hydroxyl and CH group stretching. When compared with the C–C/C–O/C–N stretching band, the predicted value of the O–H stretching band (3,737 cm^−1^) presented a slightly larger gap with the experimental band in the CHCl_3_ phase (3,433 cm^−1^). In spite of quantitative limitation, the qualitative agreement between the simulation and the experiment was achieved under our computational method.

**TABLE 2:** Simulated IR frequencies (in cm^−1^) of *epi-CD* (gas phase) at the B3LYP/6-311+G(d,p) level of theory.

S: No.	Simulated	Experimental	Approximate assignment
1	3,792	3,433	υ O-H
2	3,084, 3,066	3,079	υ C-H (unsym)
3	3,025, 3,029	3,049	υ C-H
4	1621	1639	υ C-C; β C-H
5	1506	1450	υ C-C; Wag C-H
6	1116, 1105, 1160	1165	υ C-C, C-O; β C-H, O-H
7	917	918	υ C-O, C-N; β C-H

υ, stretching; β, out-of-plane bending; Wag, wagging; unsym, unsymmetrical.

The UV-Vis spectrum of epi-CD describing the electronic transitions was acquired through time-dependent (TD)-DFT calculations at B3LYP/6-31+G(d,p) and 6-311+G(d,p) levels of theory. The simulation was performed both in a vacuum and in CHCl_3_ as solvent media to predict absorption wavelengths including λmax, electronic excitation values [such as excitation energies (E), oscillator strengths (f), and major contributions of the transitions], and their assignments (electronic transitions) in Table 3. The simulated UV-Vis spectra of epi-CD in double zeta showed two absorption bands at 288.83 and 222.48 nm. The strong absorption band peak was 284 approximate to the simulated spectrum (double zeta: 288.84 nm, triple zeta: 289.14 nm in CHCl_3_ solution). The verified spectrum through the experimental UV-Vis spectrum was used as the standard for the comparison with spectra of each epi-CD-X complex.

**TABLE 3:** Experimental and calculated electronic excitations of *epi-CD*.

Peak	Calc. λ (nm)		Exp. λ (nm)	Excitation energy (eV)		Oscillator strength		Electronic transition	
	t-zeta	d-zeta	CHCl3	t-zeta	d-zeta	t-zeta	d-zeta	t-zeta	d-zeta
1	289.14	288.83	284	4.2881	4.2926	0.1059	0.1116	H_−1_→L	H_−1_→L
2	235.02	222.48	237	5.2755	5.5729	0.0156	0.6821	H_−5_→L	H_−1_→L1

*d*-zeta, TD-B3LYP/6-31+G(d,p) in the CHCl_3_ PCM; *t*-zeta, TD-B3LYP/6-311+G(d,p) in the CHCl_3_ PCM.

**Substituent Effect on Pnicogen Bonding of *epi-CD-X*.** During the study, we had three questions, (1) “ Is a pnicogen bond applicable for controlling the enantiotopic face in the chiral scaffold?”, (2) “Can a pnicogen bonding also make an effective interaction as hydrogen bonding or halogen bonding with the chiral scaffold?”, and (3) “If the pnicogen bonding can have an effective force, which functional group is the most promising in a cinchona alkaloid?” To get answers for these questions, the dataset for our simulation was considered, and the efficient design of the simulation was possible through sampling representative analytes. For sampling enough number of cases, the next three factors were considered: 1) the aspect of the pnicogen bonding according to substituent diversity, PH_3_, and substituted phosphanes with eight kinds of substituents (X = F, Br, Cl, CF_3_, CN, OH, NO_2_, and CH_3_), 2) the spectral aspect of the bonding according to an element type of group 15, and 3) the comparison of the pnicogen bonding with the hydrogen bonding in the epi-CD-X complex. For the purpose, Q_Mulliken_ and Q_NBO_, HOMO–LUMO interaction, and UV-Vis spectra were simulated for the mixture of epi-CD and analyte (X) at TD-B3LYP/6-31+G(d,p) and B3LYP/6-31+G(d,p) levels of theory.

**FIGURE 4:**
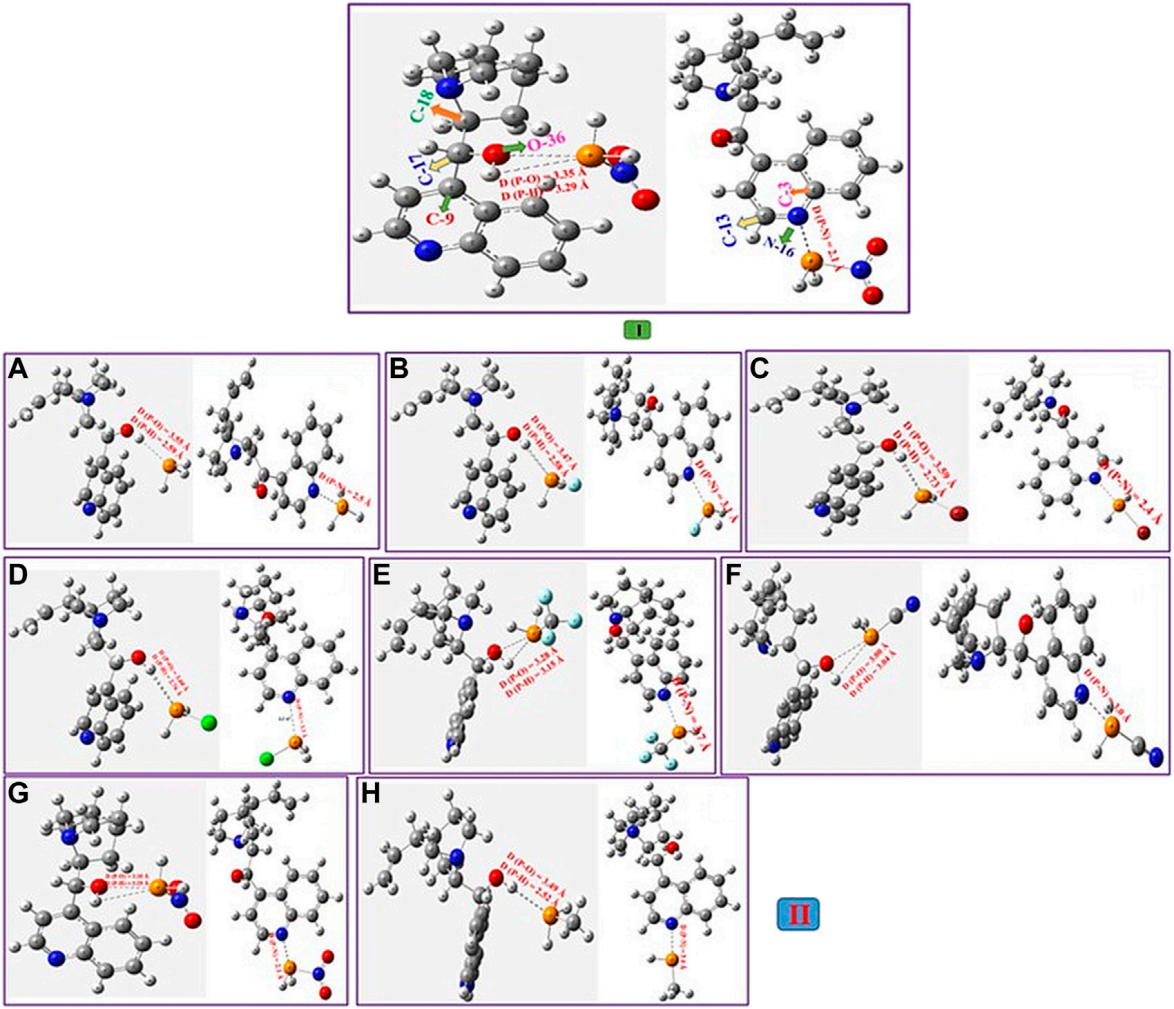
Geometry analysis of the epi-cinchonidine complex (epi-CD-X1/epi-CD-X2) with the pnicogen bond.

Firstly, the binding characteristics of the analytes, phosphane derivatives complexed with epi-CD, were investigated. They interacted with two functional groups of epi-CD, the hydroxyl group (X-epi-CD1) and the quinoline ring (X-epi-CD2). The distances of the pnicogen bonding are ca. 2.6–3.6 Å between the hydroxyl group and phosphorus in X-epi-CD1 and ca. 2.1−4.0 Å between quinoline nitrogen (N_17_) and phosphorus in X-epi-CD2 in Figure 4 and Supplementary Figures S7–S22. Even though the stability of non-covalent bonds partially depends on charge transfer from the electron-donor atom to the σ* antibonding orbital of the acceptor, electrostatic attraction also needs to be considered. In the case of the pnicogen bond, a σ-hole of the pnictide element is not essential so that the electrostatic potential of the whole complex is considered rather than the charge of binding atoms (Scheiner, 2013a). From this point, the amount of charge transfer is simulated from Mulliken and NBO charge analyses (Bibi et al., 2015; Guo and Wong, 2017). In Table 4, the charge transfers (Q_NBO_ and Q_Mulliken_) between epi-CD and an analyte present less deviation in X-epi-CD1 (up to 0.117 and 0.311 e^−^) than the values in X-epi-CD2 (up to 0.467 and 0.179 e^−^). Even though electron-withdrawing substituents tend to present the enhanced charge transfer, the highly steric hindered nitro group showed bigger Q_NBO_ and Q_Mulliken_ in X-epi-CD2 (N_16_) than in X-epi-CD1 (O_36_). In mono-substituted phosphine, XPH_2_, σ*(XP) was the LUMO that withdrew the electrons from the HOMO of N_16_ of quinoline or hydroxyl (−O_36_H_37_) at C_17_. In NO_2_PH_2_ and CNPH_2_, the LUMO was dπ* and resulted from the binding between the π orbital of the cyano or nitro substituent and the d orbital on phosphorous, and σ*(XP) became LUMO+1. The ΔE_int_ and ΔE_int_,_CP_ could show a moderately strong pnicogen bond between PH_3_ and O_36_/N_16_ of epi-CD. The strength comparison of the two pnicogen bonds (P···N and P···O) in both ΔE_int_,_CP_ (O_36_ vs. N_16_) and distance (O_36_ vs. N_16_) can propose the pnicogen bonding of X-epi-CD2 is generally stronger than the bonding of X-epi-CD1, but the electron-withdrawing substituent (σI) that is less bulky like Cl can make the pattern inversed. In the case of HOPH_2_, because the electron donating substituent, −OH, has a strong sigma inductive effect and also has the hydrogen bonding interaction, two pnicogen bonds could not be compared and showed out of the pattern. Based on the distances of HOPH_2_, X-epi-CD1 favored pnicogen bonding without a hydrogen bonding and X-epi-CD2 favored hydrogen bonding with the existence of the hydrogen bonding (Figure 4). When compared with the energy of X-epi-CD1 resulting from hydrogen bonding (Supplementary Table S3), the binding energy of X-epi-CD1 resulting from pnicogen bonding was not inferior.

**TABLE 4:** Optimized geometric parameters, ΔE_int_, ΔE_int, CP_, Q_NBO_, and Q_Mulliken_ of *X-epi-CD1/X-epi-CD2*.

Entry	X	σI	Es^a^	<C17O36H37	<C13N16C3	ΔE_int_	ΔE_int,CP_	Q_NBO_	Q_Mulliken_
1				107.6				0	0
2	PH3	0	0	108		0.15	−0.70	−0.117	0.311
3	BrPH2	0.5	−1.16	107.8		−1.44	−2.58	−0.028	0.096
4	(CF3)PH2	0.42	−2.4	107.6		−1.66	−2.22	0.049	0.117
5	(CH3)PH2	−0.04	−1.24	108		−0.12	−0.87	0.001	0.088
6	ClPH2	0.47	−0.97	107.5		−2.45	−5.03	−0.038	0.181
7	(CN)PH2	0.53	−0.51	108.8		0.19	−1.80	0.066	0.1
8	FPH2	0.52	−0.46	108.6		−3.21	−4.81	−0.037	0.072
9	(OH)PH2	0.29	−0.55	111		−5.86	−7.60	−0.057	0.059
10	(NO2)PH2	0.76	−2.52	107.6		−4.43	−7.32	−0.08	0.19
11					117.1			0	0
12	PH3	0	0		116.3	−0.25	−1.25	−0.467	−0.17
13	BrPH2	0.5	−1.16		118.3	−7.05	−9.65	−0.084	0.055
14	(CF3)PH2	0.42	−2.4		117.6	−2.77	−4.61	0.117	0.159
15	(CH3)PH2	−0.04	−1.24		117.4	−0.29	−1.42	0.055	0.109
16	ClPH2	0.47	−0.97		117.4	−1.13	−2.65	−0.195	−0.003
17	(CN)PH2	0.53	−0.51		117.3	−3.64	−5.43	−0.295	−0.063
18	FPH2	0.52	−0.46		117.6	−7.29	−10.28	−0.304	−0.109
19	(OH)PH2	0.29	−0.55		118.2	−10.56	−12.75	−0.366	−0.179
20	(NO2)PH2	0.76	−2.52		118.7	−10.78	−12.95	−0.305	−0.06

a Taft size parameter.

In sequence, the dipole moment and HOMO–LUMO energy of the X-epi-CD1 complex are further calculated in Table 5, and the substituent effect on interaction energy was compared with the effect on the band gap. Notably, the dipole moment of the complex was sensitive to the substituent type and the number of substituents, but the deviation of band gap was less than the deviation of dipole moment or interaction energy. Mono-substituted analytes were expected to present a higher dipole moment and HOMO–LUMO energy gap than di-substituted and tri-substituted analytes. An electron-withdrawing substituent also made us expect a larger HOMO–LUMO energy gap than PH_3_ based on the literature (Sarkar et al., 2015). The simulation presented the expected result in CF_3_, CN, and NO_2_ (entries 8–19) but did not explain every case. In particular, the high electronegative and small halide substituent (entries 2–7) showed the mismatch between our expectation and simulation. When relative arrangement between the analyte and epi-CD in optimal geometry was described through the angle (<O-H-P) and distances [D(P-O) and D(P-H)], the mismatch can be explained by the confounding effect between expected pnicogen bonding and hydrogen bonding. The reports on P-substituent effects on a pnicogen bond described the interaction energy grew in the order: F > Cl > OH > CF_3_ > H > CH_3_ (Scheiner, 2013a), and di- or tri-halogenation does not produce any additional stabilization, in marked contrast to H-bonds. In our X-epi-CD complex, mono-halogenation also showed the biggest band gap as well as ΔE_int,CP_ in NO_2_, CN, and CF_3_.

**TABLE 5:** Orbital energies of the HOMO and LUMO (hartree), band gap (eV), and dipole moment (D) for the *X-epi-CD1* complexes.

No.	X	HOMO	LUMO	Gap	D	**<O-H-P**	D(P-O)	D(P-H)
1	PH_3_	−0.202	−0.048	4.18	2.32	168.1	3.55	2.60
2	FPH_2_	−0.205	−0.059	3.98	1.63	152.4	3.47	2.58
3	F_2_PH	−0.210	−0.054	4.24	1.73	72.8	2.93	3.06
4	F_3_P	−0.210	−0.053	4.27	2.17	72.2	2.95	3.10
5	ClPH_2_	−0.207	−0.062	3.94	2.29	145.6	3.61	2.76
6	Cl_2_PH	−0.222	−0.058	4.46	3.48	76.8	3.19	3.27
7	Cl_3_P	−0.208	−0.074	3.65	1.56	102.9	3.37	3.02
8	BrPH_2_	−0.207	−0.063	3.91	1.99	147.6	3.59	2.73
9	Br_2_PH	−0.211	−0.079	3.60	2.37	89.3	3.19	3.05
10	Br_3_P	−0.207	−0.092	3.15	1.98	79.9	3.24	3.27
11	F_3_CPH_2_	−0.217	−0.056	4.38	2.76	88.6	3.28	3.16
12	(CF_3_)_2_PH	−0.209	−0.052	4.26	2.30	92.0	3.30	3.12
13	(CF_3_)_3_P	−0.207	−0.054	4.17	1.32	98.8	3.35	3.06
14	CNPH_2_	−0.217	−0.057	4.35	3.58	78.3	3.01	3.05
15	(CN)_2_PH	−0.216	−0.060	4.24	3.40	70.2	2.87	3.05
16	(CN)_3_P	−0.214	−0.087	3.45	3.40	94.4	3.49	3.28
17	O_2_NPH_2_	−0.207	−0.086	3.31	4.60	85.5	3.36	3.29
18	(NO_2_)_2_PH	−0.219	−0.099	3.28	4.32	71.3	2.59	3.08
19	(NO_2_)_3_P	−0.219	−0.130	2.44	4.41	66.6	2.57	2.80
20	HOPH_2_	−0.203	−0.051	4.14	2.16	163.6	3.48	2.53
21	(OH)_2_PH	−0.207	−0.059	4.01	2.77	158.9	3.41	2.48
22	(OH)_3_P	−0.204	−0.059	3.93	1.61	160.7	3.42	2.48
23	H_3_CPH_2_	−0.200	−0.046	4.20	3.04	172.9	3.49	2.53
24	(CH_3_)_2_PH	−0.199	−0.046	4.17	3.23	168.1	3.43	2.47
25	(CH_3_)_3_P	−0.199	−0.047	4.14	2.94	168.4	3.40	2.43

**FIGURE 5:**
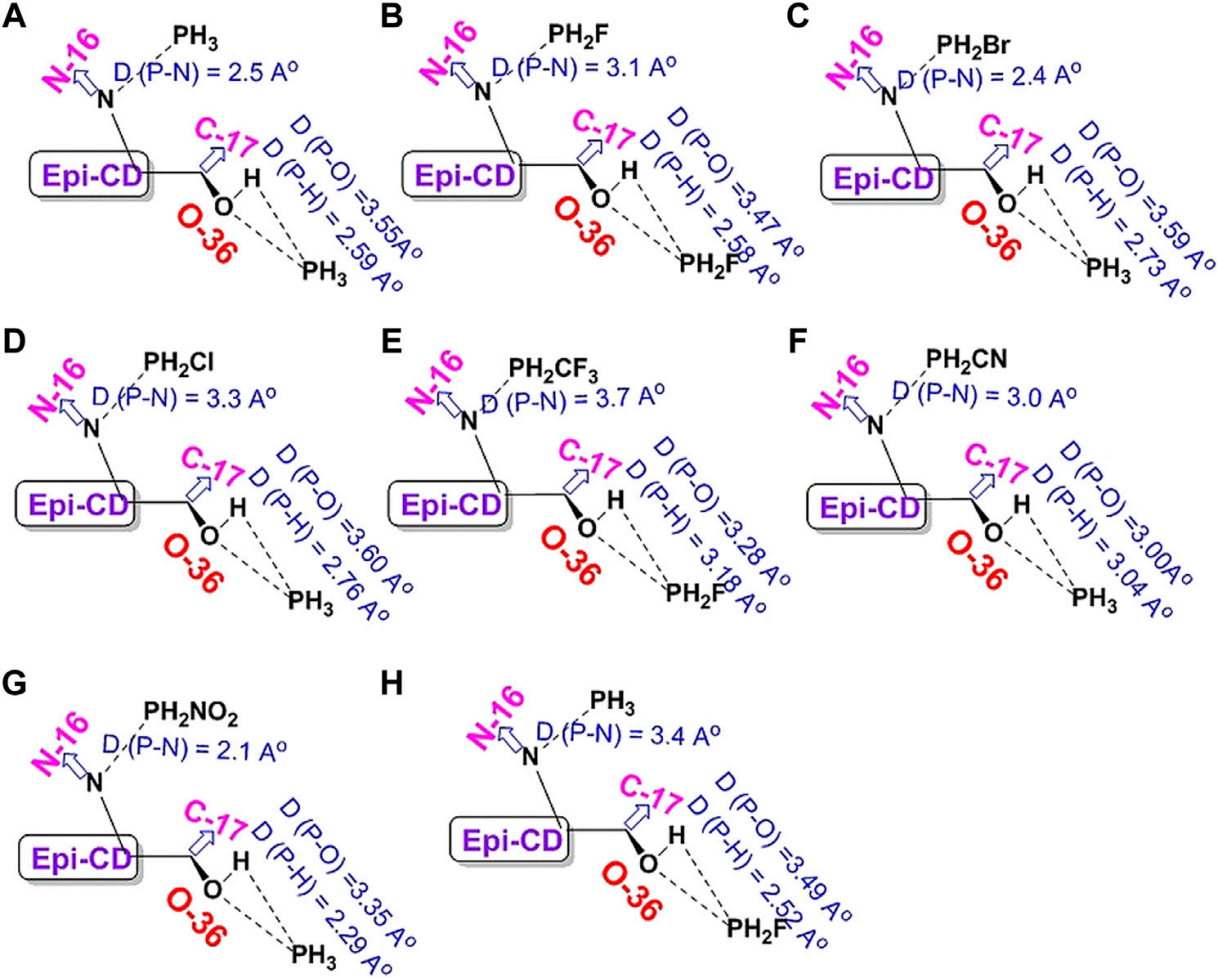
Predicted full UV-Vis spectrum of the epi-cinchonidine complex: X-axis = wavelength and Y-axis = oscillator strengths and epsilon. Experimental full UV-Vis spectrum of the epi-cinchonidine complex: X-axis = wavelength and Y-axis = observance.

**UV-Vis Spectroscopic Study of *epi-CD-X* for Pnicogen Bonding.** The aspect of the bonding according to an element type of group 15 was studied through UV-Vis spectra prediction and experimental measurement of the spectra (Figure 5). The simulated excitation energies (eV), oscillator strengths, and molecular orbitals of the first allowed singlet transition involved in the excitation for the epi-CD, X-epi-CD1, and X-epi-CD2 complexes are given in Table 6 and Supplementary Figures S22, S23. Under both gas and solution phases, the binding of the pnictide analytes (P, As, Sb, and Bi) with epi-CD at the two positions caused the red-shifted absorption energy peak (λmax). In general, a more prominent increase in λmax was predicted in X-epi-CD1 rather than in X-epi-CD2, and experimental values were closer to the values of X-epi-CD2 than X-epi-CD1 except for phosphoric acid (PA) (entries 11 and 12). It seems that the variation of λmax depends on the stereoelectronic property of an analyte so that the bulkier and lower electron density of the analyte tends to show a larger variation of the red-shift. Even though the aspect of λmax according to an element type of group 15 under the same halogen substituent (BiI_3_, SbI_3_, and AsI_3_) did not exactly match with the atomic diameter, with the difference between metallic and non-metallic elements, BiI_3_ and AsI_3_ could present more dramatic variation of red-shift than PBr_3_ and phosphoric acid. The excited state energies for the first allowed transition states were 2.06, 3.07, 2.47, 3.33, 2.10, and 3.26 eV for the AsI_3_-epi-CD1, AsI_3_-epi-CD2, SbI_3_-epi-CD1, SbI_3_-epi-CD2, BiI_3_-epi-CD1, and BiI_3_-epi-CD2 complexes, respectively. When compared with hydrogen bonding, pnicogen bonding presents a larger variation (entry 2) in Table 6. The interaction with AsI_3_ and BiI_3_ was stronger than that with other tested analytes (entries 5, 6, 9, and 10) in our simulation, and the predicted tendency matched with experimental spectra. Due to the excellent interaction, the electronic structure of epi-CD was greatly altered and more outstanding alteration was simulated in X-epi-CD1 between the hydroxyl group (X-epi-CD1) and the quinoline ring (X-epi-CD2). Notably, the experimental data were closer to X-epi-CD2 than X-epi-CD1 so that the data could support the possibility of dominant binding of the corresponding analytes with the quinoline ring.

**TABLE 6:** Calculated excitation energies, oscillator strengths, and molecular orbitals (MOs) of CD and CD-X at the TD-B3LYP/6-31+G(d,p) level of theory.

No.	Species	Peak	Energy (eV)	Calc. λ (nm)	Exp. λ (nm)	Strength	Molecular orbital	Coefficient
1	epi-CD	1	4.2926	288.83	284	0.1116	H_−1_→L	0.68341
		2	5.5729	222.48	237	0.6821	H_−1_→L_1_	0.46481
2	MA-CD	1	4.2984	288.44	285	0.1165	H_−1_→L	0.63651
		2	5.4281	228.41	232	0.0429	H_−6_→L	0.57214
3	Br_3_P-CD1	1	3.5722	347.08	318	0.0690	H→L_3_	0.67614
		2	4.1844	296.30	235	0.0233	H_−6_→L_3_	0.58271
4	Br_3_P-CD2	1	3.8103	325.39	320	0.0874	H→L_6_	0.50492
		2	5.1798	239.36	235	0.0851	H_−5_→L_2_	0.49543
5^a^	Br_3_P-CD1	1	3.4699	357.32	318	0.0134	H→L_1_	0.55407
		2	4.2389	292.49	235	0.0472	H_−1_→L_1_	0.58319
6^a^	Br_3_P-CD2	1	4.0001	309.96	320	0.0800	H_−4_→L	0.65344
		2	4.1378	299.64	235	0.0552	H_−1_→L_2_	0.65601
7	I_3_As-CD1	1	2.0623	601.19	362	0.0023	H→L	0.70694
		2	2.8463	435.6	292	0.0019	H_−1_→L	0.70322
		3	3.2812	377.86	232	0.019	H_−7_→L	0.67673
8	I_3_As-CD2	1	3.072	403.6	358	0.0189	H_−2_→L	0.60869
		2	3.3554	369.51	288	0.0213	H_−7_→L	0.66956
		3	3.9813	311.42	233	0.0564	H_−7_→L_1_	0.48214
9	I_3_Sb-CD1	1	2.4695	502.07	318	0.002	H→L	0.7069
		2	3.3973	364.94	250	0.0207	H_−3_→L	0.63835
		3	3.8764	319.84	232	0.0329	H_−1_→L_2_	0.66762
10	I_3_Sb-CD2	1	3.332	372.1	317	0.0201	H_−1_→L	0.65117
		2	4.0891	303.21	236	0.0722	H_−10_→L	0.41281
11	I_3_Bi-CD1	1	2.1036	589.38	375	0.0045	H→L	0.70669
		2	4.0479	306.29	292	0.0977	H_−1_→L_2_	0.55728
12	I_3_Bi-CD2	1	3.2635	379.91	363	0.0421	H_−1_→L	0.65687
		2	3.7971	326.52	292	0.0678	H_−1_→L_1_	0.53361
13	PA-CD1	1	4.2212	293.72	316	0.103	H_−1_→L	0.68772
		2	5.5264	224.35	235	0.6572	H_−1_→L_1_	0.50141
14	PA-CD2	1	4.1416	299.36	316	0.1062	H_−1_→L	0.69007
		2	5.5029	225.31	230	0.7259	H_−1_→L_1_	0.52953

a Basis set was 6–311+G(d,p).

**AIM Analysis of epi-CD-X.** The quantum theory of atoms in molecules (AIM) has been widely used to analyze the real space functions and characterize the different types of interactions. Herein, the NCIs of the epi-CD complex with PH_2_CN, as a representative X-epi-CD, were analyzed according to Bader’s AIM theory (Bader, 1985), using the Multiwfn code (Lu and Chen, 2012). It is known that the isosurface of the reduced density gradient (RDG) is a valuable tool for delicately revealing NCI regions (particularly weak interactions) based on the next dimensionless equation given as follows:

$$\text{RDG}\left(\text{r}\right)=\frac{\left|\nabla \rho \left(r\right)\right|}{\rho^{\frac{4}{3}}\left(r\right)}.$$ (3)

**FIGURE 6:**
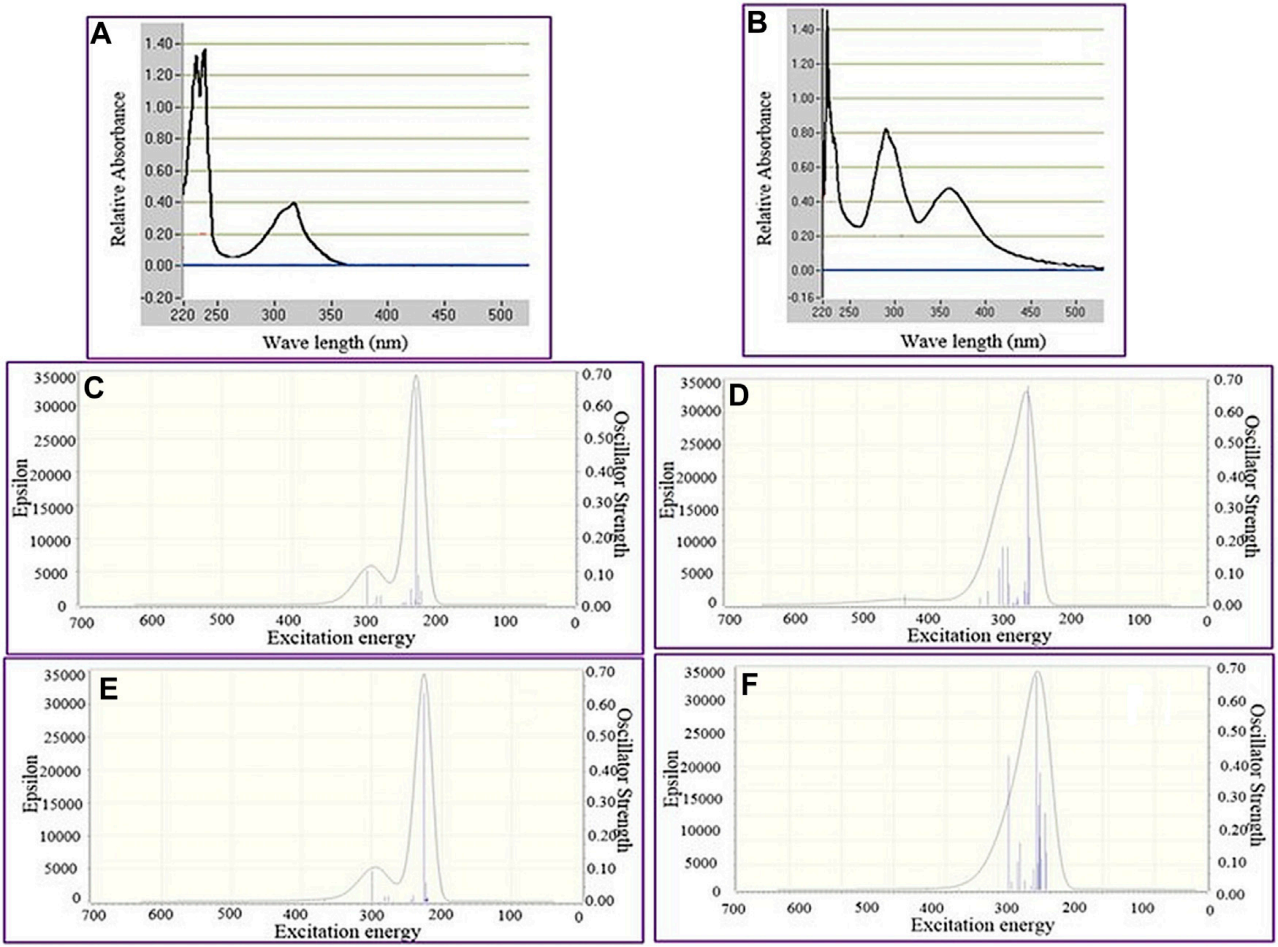
AIM analysis of the epi-CD-X complex. RDG isosurface maps: **(A)** PH_2_CN–OH as a representative X-epi-CD1 (X: PH_2_CN, OH: O_36_H_37_ at C_17_) and **(B)** PH_2_CN–N as a representative X-epi-CD2 (X: PH_2_CN, N: N_16_ of quinoline). Display of critical points: **(C)** PH_2_CN–OH (as X-epi-CD1) and **(D)** PH_2_CN (as X-epi-CD2). NCI plot of the epi-CD-X complex: **(E)** PH_2_CN–OH (as X-epi-CD1) and **(F)** PH_2_CN (as X-epi-CD2).

The sign of *λ*_2_, the second largest eigenvalue of the Hessian matrix of electron density, discriminates (3, −1) type critical point (CP), which appears in the chemical bond path or between atom pairs that have a weak attractive interaction, from (3, +1) type CP, which appears in the ring center or displays a steric effect in Bader’s AIM theory. As shown in 2D plots of the RDG with the sign of *λ*_2_ (Figure 6), RDG isosurfaces having the values of Λ(r) of the following equation can show the region of the interaction and also the type and strength:

$$\text{Λ}\left(\text{r}\right)=Sign\left[\lambda_{2}\left(r\right)\right]\rho \left(r\right).$$ (4)

**FIGURE 7:**
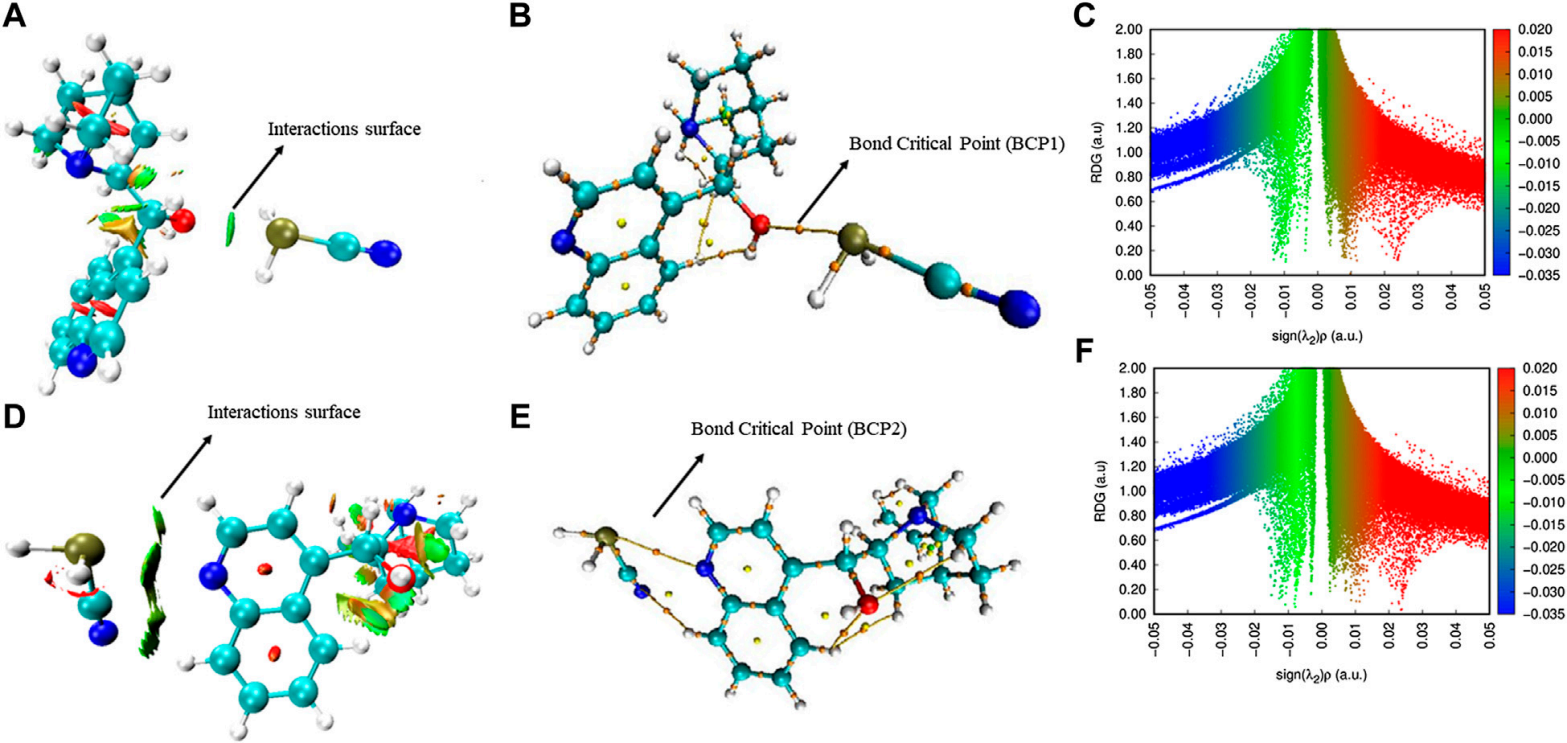
Proposed models of the cinchona alkaloid–catalyzed asymmetric reaction.

In general, blue, red, and green (or earth green) colors indicate the strong attractive, strong repulsive, and van der Waals interactions, respectively. In other words, Figures 6A,B indicate the pnicogen bonding interactions between epi-CD and PH_2_CN show the strength between strong, attractive, and van der Waals interactions. Furthermore, the density of electrons (*ρ*) and Laplacian of electron density (Δ^2^ρ) at bond critical points (BCPs) in Figures 7C,D were calculated. For example, in the case of hydrogen bonding, *ρ* and Δ^2^ρ typically varied in the range from 0.002 to 0.04 a.u. and from 0.020 to 0.139 a.u., respectively, in AIM analysis (Roohi et al., 2011). In the epi-CD complex, *ρ* and Δ^2^ *ρ* were calculated to be 0.00074762 a.u. and Laplacian of electron density was 0.013382 a.u. at BCP1 (PH_2_CN–OH as X-epi-CD1). In contrast to BCP1, the density of electrons was 0.00011455 a.u. and the Laplacian of electron density at BCP2 was 0.0012619 a.u., respectively, which shows the corresponding interactions (PH_2_CN–N as X-epi-CD2). The two-dimensional NCI plots of RDG with Λ(r) were achieved (Figures 6E,F). The results are similar to what we have obtained from the abovementioned results. Peaks appear in the range from *ρ* ≃ 0.01 to *ρ* ≃ 0.05 a.u. for the interactions. In brief, the AIM analysis clearly showed the current X-epi-CD has less sufficient strength rather than known hydrogen bond–controlled reactions. For the improved feasibility, innovative structural modification is required for either analytes or cinchona alkaloids. Fortunately, X-epi-CD1, able to control chirality, has more promising *ρ*, Δ^2^ *ρ*, and Λ(r) for the improvement (ca. 50% of hydrogen bonding).

**Conceptual Sketch of X-epi-CD.** To improve the insufficiency of the X-epi-CD system, we considered the sketch based on how to revise this system. In the literature (Tanriver et al., 2016), it has been proposed that cinchona alkaloids tend to have complexes with substrates through hydrogen bonds in the hydroxyl group (H-O_36_) or quinuclidine (H^+^-N_43_) and long range interactions at conjugated pi electrons to produce the activation of substrate reactivity as well as enantioselectivity. For example, two models of conjugate addition reaction of thiolate (Wynberg’s and Grayson/Houk’s) elucidated the mediation of two protons between an electrophile/nucleophile and a cinchona alkaloid in the asymmetric catalysis as shown in Figure 7 (left). If such mediation of the hydrogen atom can be replaced with other elements, enantiotopic selectivity of the complexes also can be archived by new types of non-covalent bonds. Through the experimental data as well as simulation study, the dominant site of pnicogen bonding in epi-CD is the quinoline ring (N_16_ atom) rather than the hydroxyl group (O_36_ atom) showing P···O distance longer than P···N distance in almost all complexes. Because the nitrogen atom of the quinoline ring is very far from the complex and dense chiral environment of the cinchona alkaloid, it is rarely possible that X-epi-CD2 controls any enantiotopic face regardless of strength of the interaction. However, if the design of an analyte can modify the ratio of X-epi-CD1 (major) to X-epi-CD2 (minor) to reduce X-epi-CD2, the problem of uncontrolled stereoselectivity can be overcome. In particular, the HOMO–LUMO band gap energy as well as binding energy sometimes presented reversed pattern between X-epi-CD1 and X-epi-CD2. For example, the binding energy of ClPH_2_ (pnicogen bond) is similar to the binding energy of MA (hydrogen bond) at the same position, the hydroxyl group (O_36_ atom) in Supplementary Table S3. Moreover, AIM analysis of the PH_2_CN epi-CD1 complex also showed less difference from hydrogen bonding rather than epi-CD2 in *ρ* and Δ^2^ *ρ*. It makes us propose the preliminary sketch of enantioselective reaction in which a pnicogen bonding of the cinchona alkaloid assigns enantiotopic selectivity into a substrate. Based on the literature (Yaghoobi and Sohrabi−Mahboub, 2018; LiXueCheng, 2017; Grayson and Houk, 2016b), the plausible idea is that the pnicogen bonding catalyzed the enantioselective aza-Diels–Alder reaction in Figure 7. For an ideal example, the complex X-epi-CD1 can have desirable interactions with a dienophile through pnicogen bonding as well as hydrogen bonding. In that situation, the enantiotopic face can be formed through the triangle composition of the 1) quinuclidine core, 2) chiral OH group of epi-CD, and 3) dienophile (or pnictide atom) because a face can be defined by three points. At that time, a diene can have the bias among front side and back side of the enantiotopic face. After pre-activating the preferred complex X-epi-CD1 (having a P···O bond), a diene and a dienophile can be added into the complex. If the dienophile has a substituent (e.g., NH_2_) bearing an unshared pair of electrons at the α-position, the strength of the pnicogen bond can be more enhanced through another NCI bond (P-H···N bond) with a dienophile. At that time, the mediation of phosphane (through the pnicogen bond) can make the complex able to discriminate the enantiotopic face as described in Figure 7. In other words, the behind face of dienophile is less favored by the attack of diene. While the σ-hole of halogen bonding is essential at the halogen atom, the pnicogen bond does not require such a strong σ-hole at the pnictogen atom (Scheiner, 2013a). Rather than electrostatic attraction, it is expected that the geometry for charge transfer is critical between atoms of the NCIs. To make the current theoretical reaction more feasible, the four components (epi-CD, analyte, dienophile, and diene) need to be modified to be an entropically favored form.

## Conclusion

In this study, at B3LYP/6-31G(d) and B3LYPgCP-D3/6-31G levels of theory, intermolecular interactions of epi-CD with analytes (X) were described by the geometrical parameters and electronic, thermodynamic, and charge analyses. O_36_ of the hydroxyl group (in X-epi-CD1) and N_16_ of the quinoline ring (in X-epi-CD2) among Lewis basic atoms of epi-CD could be the interacting atoms of pnicogen bonds. While the dominant force of hydrogen bonding generally is electrostatic attraction, HOMO–LUMO energy, Q_NBO_, Q_Mulliken_, AIM analysis, and UV-Vis analysis of the pnicogen bonds elucidated the interaction including the polarization and electron transfer. Based on the results, researchers can further progress pnicogen-based asymmetric catalysis based on our study in the recent future.

## Data Availability

The original contributions presented in the study are included in the article/Supplementary Material, and further inquiries can be directed to the corresponding author.

## Abbreviations

CD, cinchonidine; X, phosphine (PH3)-substituted analytes (F, Br, Cl, CF_3_, CN, HO, NO_2_, and CH_3_); HOMO, highest occupied molecular orbital; LUMO, lowest unoccupied molecular orbital; TD, time-dependent; DFT, density functional theory; X-epi-CD1, analytes bonding with the hydroxyl group (O-36); X-epi-CD2, analytes bonding with the quinoline ring (N_16_); DOS, density of states; IP, ionization potential; EA, electron affinity; PNBA, 4-nitrobenzoic acid; DEAD, diethyl azodicarboxylate; PPh_3_, triphenylphosphine; DCM, dichloromethane; LiOH, lithium hydroxide, CHCl_3_, chloroform; EtOH, ethanol; DW, distilled water; MEP, molecular electrostatic potential; NBO, natural bonding orbital; IR, infrared; MA, methyl acrylate, PBr_3_, phosphorus tribromide; BiI_3_, bismuth(III) iodide; SbI_3_, antimony triiodide; AsI_3_, arsenic triiodide.
