# Supplementary material for: Plausible Pnicogen Bonding of epi-Cinchonidine as a Chiral Scaffold in Catalysis
Source: Front Chem. 2021 Jul 6;9:669515. doi: 10.3389/fchem.2021.669515 (PMC8290064; doi:10.3389/fchem.2021.669515)
Supplement: Supplementary file 1 [file DataSheet2.docx]

Plausible Pnicogen Bonding of *epi*-Cinchonidine as a Chiral Scaffold in Catalysis

Zakir Ullah^1,2, ǂ^, Kang Kim^1,ǂ^, Arramshetti Venkanna^1,ǂ^, Hye su Kim^3^, Moon Il Kim^3^ and Mi-hyun Kim^1*^

^1^Gachon Institute of Pharmaceutical Science & Department of Pharmacy, College of Pharmacy, Gachon University, 191 Hambakmoeiro, Yeonsu-gu, Incheon, Republic of Korea, ^2^Department of Chemistry, Korea Advanced Institute of Science and Technology, Daehak-ro 291, Yuseong-gu, Daejeon, Republic of Korea, ^3^Department of BioNano Technology, Gachon University, Seongnam, Gyeonggi 13120, Republic of Korea

^*^Author for correspondence E-mail: Mi-hyun Kim: [kmh0515@gachon.ac.kr](mailto:kmh0515@gachon.ac.kr)

^ǂ^ The authors are co-first authors

The Cartesian Coordinate of Simulated Complexes.

BR2PHCD1COMPLEXOPT.LOG.out

xyz generated by gcpd3 webservice:

C -2.943139 -3.925369 -2.237291

C -3.456776 -4.094125 -0.973512

C -3.100966 -3.211991 0.081322

C -2.196239 -2.123505 -0.178771

C -1.677397 -1.989848 -1.499270

C -2.043952 -2.864873 -2.497292

H -3.221008 -4.606314 -3.036414

H -4.144179 -4.898389 -0.733622

C -1.878418 -1.257502 0.919683

H -0.972261 -1.196850 -1.705453

H -1.633618 -2.741374 -3.495324

C -2.447542 -1.548723 2.144731

C -3.326310 -2.644790 2.287784

H -2.225293 -0.931560 3.011048

H -3.767788 -2.854224 3.261590

N -3.655394 -3.455640 1.304889

C -0.996726 -0.009657 0.849166

C -1.802717 1.260712 0.463997

C -2.018914 1.505717 -1.057836

H -2.781675 1.120588 0.933132

C -0.062080 2.979434 0.395094

C -2.287628 3.512988 1.106778

C -1.887951 3.021564 -1.315733

H -1.264025 0.983942 -1.651649

H -2.999045 1.133457 -1.369216

H 0.346392 3.812876 0.976711

H 0.691988 2.190705 0.385746

C -0.425627 3.427766 -1.053667

C -2.800416 3.819462 -0.339089

H -1.860361 4.414084 1.558475

H -3.098487 3.186601 1.763315

H -2.166867 3.250726 -2.350762

H -0.307957 4.511253 -1.175852

H 0.232376 2.949514 -1.788298

H -2.631777 4.885359 -0.555880

O 0.140066 -0.160186 -0.010604

H 0.569575 -1.000551 0.210470

C -4.263809 3.560397 -0.616149

H -4.550021 3.756558 -1.651714

C -5.218070 3.145788 0.218250

H -5.040134 2.930871 1.267526

H -6.238178 3.009633 -0.127679

N -1.244433 2.470538 1.117196

H -0.647098 0.186615 1.871625

P 3.267573 0.429034 0.240166

H 2.811097 0.468267 -1.104644

Br 5.437488 0.738022 -0.280601

Br 3.078828 -1.829472 0.419042

BR2PHCD2COMPLEXOPT.LOG.out

xyz generated by gcpd3 webservice:

C -0.627875 3.439821 0.559541

C -1.284377 2.239613 0.422155

C -0.633952 1.124579 -0.168516

C 0.732203 1.244149 -0.606218

C 1.368231 2.510908 -0.459933

C 0.704363 3.575846 0.106262

H -1.132832 4.286052 1.015751

H -2.298650 2.099349 0.778207

C 1.355048 0.078581 -1.162646

H 2.381437 2.626721 -0.817647

H 1.211546 4.530801 0.207519

C 0.589606 -1.068583 -1.259279

C -0.747284 -1.080699 -0.814312

H 1.012507 -1.975101 -1.683175

H -1.341754 -1.988573 -0.903488

N -1.351314 -0.034040 -0.289309

C 2.811143 -0.027566 -1.623937

C 3.758513 -0.486818 -0.484975

C 4.239857 0.622723 0.494820

H 3.179180 -1.222845 0.082009

C 5.954500 -0.348842 -1.562305

C 5.489726 -2.034001 0.081360

C 5.720222 0.343300 0.831914

H 4.166442 1.612930 0.037820

H 3.622706 0.629136 1.398046

H 6.726797 -0.977597 -2.018326

H 5.508002 0.252053 -2.354998

C 6.554072 0.558348 -0.444609

C 5.900244 -1.132340 1.291962

H 6.361987 -2.566567 -0.311019

H 4.760960 -2.794974 0.373109

H 6.056744 1.020119 1.625833

H 7.605202 0.311328 -0.252470

H 6.521449 1.612876 -0.740718

H 6.974914 -1.268641 1.486920

O 3.321747 1.163918 -2.223440

H 2.782027 1.360760 -3.000642

C 5.198138 -1.388722 2.606068

H 5.485014 -0.689318 3.394247

C 4.312191 -2.336305 2.915954

H 3.969208 -3.085057 2.208577

H 3.895073 -2.401911 3.916203

N 4.911675 -1.248361 -1.025938

H 2.849424 -0.845707 -2.356218

P -4.100048 -0.710833 -0.245461

H -3.985897 0.438303 -1.068086

Br -6.224183 -1.185696 -0.940396

Br -4.480808 0.453121 1.663793

BR3PHCD1COMPLEXOPT.LOG.out

xyz generated by gcpd3 webservice:

C 1.449073 -4.744033 1.166061

C 0.796716 -4.676769 -0.043612

C 0.620605 -3.434215 -0.706764

C 1.124547 -2.235090 -0.102165

C 1.795351 -2.345087 1.146580

C 1.954085 -3.566818 1.763868

H 1.581902 -5.700384 1.663120

H 0.402942 -5.561104 -0.533287

C 0.913197 -1.000840 -0.793160

H 2.211298 -1.461566 1.617224

H 2.476874 -3.626890 2.713884

C 0.274527 -1.058341 -2.016327

C -0.171096 -2.300200 -2.530268

H 0.071630 -0.152032 -2.574297

H -0.684593 -2.326438 -3.490636

N -0.023227 -3.450861 -1.911622

C 1.300167 0.353617 -0.219168

C 2.846562 0.628043 -0.149018

C 3.454444 0.717818 1.291706

H 3.349289 -0.189540 -0.677490

C 2.725751 3.052776 -0.185604

C 4.610495 1.909842 -1.137579

C 4.364672 1.960578 1.356365

H 2.661750 0.809976 2.040703

H 4.034509 -0.182062 1.520767

H 2.891846 3.914931 -0.839608

H 1.646609 2.990825 -0.015339

C 3.489168 3.210951 1.165610

C 5.413566 1.917427 0.206022

H 4.824189 2.816804 -1.713023

H 4.889921 1.062825 -1.769168

H 4.879144 1.997822 2.323671

H 4.114479 4.112350 1.163074

H 2.787403 3.309054 2.000520

H 5.983647 2.856562 0.271520

O 0.574377 0.637252 0.990898

H 0.503991 -0.173294 1.510247

C 6.413721 0.802669 0.410672

H 6.949757 0.870402 1.359693

C 6.716501 -0.215256 -0.396458

H 6.248164 -0.368593 -1.363546

H 7.469280 -0.944483 -0.112880

N 3.155368 1.846546 -0.918703

H 0.916935 1.083133 -0.936849

P -2.733590 0.943793 0.598560

Br -4.926857 1.264688 1.043182

Br -2.625161 1.552355 -1.576110

Br -2.627394 -1.303483 0.458295

BR3PHCD2COMPLEXOPT.LOG.out

xyz generated by gcpd3 webservice:

C 0.000625 3.532089 0.820814

C -0.668277 2.342290 0.656003

C -0.046744 1.245257 0.002991

C 1.307008 1.374331 -0.469398

C 1.957919 2.629507 -0.291722

C 1.320230 3.676621 0.334568

H -0.484259 4.363296 1.324183

H -1.673102 2.196364 1.035080

C 1.904076 0.227857 -1.089713

H 2.961828 2.751448 -0.673190

H 1.838264 4.623120 0.458270

C 1.126934 -0.908900 -1.205267

C -0.196500 -0.930599 -0.719987

H 1.529328 -1.801383 -1.676651

H -0.798815 -1.830071 -0.825621

N -0.778821 0.098048 -0.137599

C 3.343617 0.130571 -1.601314

C 4.325487 -0.375958 -0.512807

C 4.857712 0.695804 0.482302

H 3.758105 -1.122612 0.052170

C 6.483304 -0.228538 -1.662376

C 6.054608 -1.962855 -0.060632

C 6.344075 0.384439 0.760391

H 4.785281 1.700033 0.056815

H 4.271280 0.684508 1.405671

H 7.228781 -0.849777 -2.170482

H 6.014950 0.407150 -2.414413

C 7.137890 0.629140 -0.536404

C 6.517382 -1.108191 1.165291

H 6.907005 -2.490277 -0.500869

H 5.328151 -2.726376 0.230315

H 6.717389 1.029934 1.563908

H 8.190193 0.357345 -0.389360

H 7.114776 1.693650 -0.795363

H 7.595615 -1.265840 1.320661

O 3.843432 1.340053 -2.174213

H 3.270143 1.575958 -2.915673

C 5.854337 -1.398319 2.492635

H 6.181351 -0.732955 3.294604

C 4.959551 -2.338625 2.799218

H 4.576964 -3.054288 2.078006

H 4.574457 -2.431321 3.810136

N 5.448574 -1.133393 -1.119313

H 3.351796 -0.660327 -2.363926

P -3.824122 0.021145 -0.379364

Br -6.022194 0.149315 -0.948651

Br -3.590348 -2.225762 -0.231999

Br -3.953538 0.593496 1.803242

c2f6phcd1complexoptoutput.LOG.out

xyz generated by gcpd3 webservice:

C 0.391543 -4.273477 1.809523

C 1.441925 -4.645841 1.004652

C 1.954149 -3.761150 0.018262

C 1.370027 -2.454527 -0.135607

C 0.281664 -2.109182 0.717139

C -0.192233 -2.993870 1.659573

H 0.005950 -4.959261 2.558261

H 1.914626 -5.618813 1.087477

C 1.923527 -1.603477 -1.148674

H -0.181250 -1.139876 0.609608

H -1.028648 -2.701633 2.287030

C 2.965283 -2.108714 -1.903678

C 3.469322 -3.404721 -1.660317

H 3.407855 -1.509134 -2.694809

H 4.295359 -3.780674 -2.263221

N 2.995119 -4.215975 -0.738884

C 1.485896 -0.169699 -1.458515

C 2.298186 0.884228 -0.664963

C 1.868435 1.113947 0.814477

H 3.323792 0.500505 -0.674588

C 1.124937 2.952067 -1.270888

C 3.480541 2.955311 -0.827565

C 1.899600 2.631450 1.089310

H 0.855945 0.747991 0.996006

H 2.538249 0.577621 1.493108

H 1.248515 3.860715 -1.870203

H 0.313644 2.373995 -1.713921

C 0.818771 3.299283 0.219349

C 3.283615 3.226001 0.700912

H 3.540178 3.899444 -1.378709

H 4.410204 2.414763 -1.024295

H 1.702700 2.821389 2.150871

H 0.818387 4.383761 0.382882

H -0.168838 2.927404 0.513112

H 3.209980 4.312749 0.860823

O 0.082948 0.059653 -1.283774

H -0.395404 -0.590820 -1.815307

C 4.373972 2.738553 1.627254

H 4.148501 2.897567 2.683947

C 5.542140 2.170265 1.325408

H 5.865956 1.977993 0.307129

H 6.236235 1.877336 2.107289

N 2.371995 2.170370 -1.401542

H 1.747005 0.018741 -2.509034

P -3.152625 0.395435 -0.737824

H -3.024999 1.802145 -0.851179

C -2.944323 0.366678 1.148593

F -1.725688 0.867422 1.455188

F -2.988835 -0.904680 1.596895

F -3.855525 1.072251 1.838274

C -5.048507 0.401448 -0.792090

F -5.525116 -0.721641 -0.220023

F -5.426086 0.405473 -2.088781

F -5.640836 1.451078 -0.198737

c2f6phcd2complexoptoutput.LOG.out

xyz generated by gcpd3 webservice:

C -0.668557 3.654746 0.920878

C -1.384368 2.552984 0.516625

C -0.756163 1.504373 -0.207117

C 0.649037 1.591950 -0.508619

C 1.351175 2.756461 -0.080561

C 0.708311 3.756045 0.614423

H -1.158897 4.450742 1.473651

H -2.439805 2.446130 0.738165

C 1.237797 0.499102 -1.226916

H 2.399586 2.850999 -0.326411

H 1.265717 4.633479 0.929393

C 0.405907 -0.541228 -1.594867

C -0.961241 -0.529168 -1.247324

H 0.801133 -1.385573 -2.153465

H -1.598591 -1.362098 -1.535742

N -1.538285 0.446763 -0.577147

C 2.718144 0.354648 -1.589110

C 3.521017 -0.396297 -0.496315

C 3.953903 0.448194 0.737860

H 2.847725 -1.189140 -0.154495

C 5.822233 -0.200411 -1.316734

C 5.102217 -2.146258 -0.113803

C 5.376751 0.004948 1.138315

H 3.973889 1.514709 0.499549

H 3.249714 0.307293 1.562996

H 6.606036 -0.779965 -1.816487

H 5.494368 0.576588 -2.007591

C 6.344541 0.420974 0.015189

C 5.438825 -1.542881 1.290020

H 5.979382 -2.651807 -0.530648

H 4.308329 -2.895585 -0.052120

H 5.663758 0.479399 2.083982

H 7.359383 0.074660 0.245708

H 6.386624 1.513693 -0.056148

H 6.481898 -1.789272 1.540698

O 3.367494 1.589015 -1.899817

H 2.891425 1.995506 -2.636143

C 4.603047 -2.014038 2.458142

H 4.866780 -1.530557 3.401306

C 3.623147 -2.918382 2.483176

H 3.289752 -3.459982 1.603277

H 3.111496 -3.158178 3.410319

N 4.683418 -1.111970 -1.078292

H 2.765052 -0.307111 -2.465232

P -5.033911 0.045598 -0.837725

H -6.454975 0.040925 -0.845310

C -4.935770 0.516198 1.000353

F -4.874520 1.869414 1.083977

F -3.833038 0.014931 1.576391

F -5.995844 0.127221 1.733735

C -4.935654 -1.836478 -0.618005

F -3.656373 -2.252769 -0.526776

F -5.473937 -2.406115 -1.720210

F -5.591044 -2.322393 0.451725

c3f9phcd1complexoptoutput.LOG.out

xyz generated by gcpd3 webservice:

C 0.599838 -4.363850 1.646512

C 1.605810 -4.748269 0.792363

C 2.152611 -3.835098 -0.148817

C 1.651030 -2.487291 -0.204629

C 0.612172 -2.127366 0.702909

C 0.101388 -3.040833 1.597947

H 0.185985 -5.071592 2.358814

H 2.015873 -5.752631 0.800354

C 2.228174 -1.611389 -1.183011

H 0.222169 -1.120825 0.677417

H -0.699384 -2.740405 2.267014

C 3.218700 -2.131460 -1.994619

C 3.648233 -3.467581 -1.842210

H 3.675650 -1.513570 -2.763181

H 4.435588 -3.854786 -2.488167

N 3.144676 -4.304865 -0.960340

C 1.856767 -0.142496 -1.405002

C 2.747310 0.827434 -0.592144

C 2.438038 0.948174 0.927226

H 3.757648 0.419789 -0.704983

C 1.636108 2.996417 -0.860019

C 4.024248 2.839745 -0.729471

C 2.677631 2.417256 1.339178

H 1.398126 0.691079 1.142520

H 3.071344 0.265109 1.500954

H 1.700780 3.926682 -1.434918

H 0.736378 2.472837 -1.182622

C 1.593698 3.283963 0.672481

C 4.066675 2.903980 0.833284

H 4.040331 3.849594 -1.151983

H 4.892857 2.314980 -1.136359

H 2.627761 2.513769 2.430004

H 1.778401 4.344185 0.884589

H 0.608624 3.040172 1.082545

H 4.157033 3.957009 1.140976

O 0.474473 0.142977 -1.169310

H -0.048890 -0.443012 -1.732190

C 5.193846 2.167765 1.520766

H 5.136623 2.209518 2.610629

C 6.219209 1.505298 0.983462

H 6.372558 1.412046 -0.087335

H 6.963544 1.027222 1.613008

N 2.806468 2.169047 -1.223467

H 2.091604 0.087811 -2.453457

P -2.794824 -0.000302 -0.448695

C -2.271517 1.367317 0.765888

F -1.859780 2.433314 0.052495

F -1.238678 0.928305 1.510502

F -3.231182 1.776428 1.609528

C -3.862966 -1.014111 0.755852

F -3.092526 -1.362904 1.808684

F -4.264498 -2.137616 0.137435

F -4.951632 -0.389785 1.226814

C -4.197264 0.941084 -1.323732

F -5.032791 0.029806 -1.861009

F -3.665526 1.667585 -2.323691

F -4.917882 1.766458 -0.552686

c3f9phcd2complexoptoutput.LOG.out

xyz generated by gcpd3 webservice:

C 0.052454 3.444261 1.666619

C -0.689140 2.500112 0.997047

C -0.075895 1.632398 0.055208

C 1.337232 1.739894 -0.198151

C 2.066017 2.739401 0.510158

C 1.439234 3.563518 1.417536

H -0.425923 4.103480 2.384950

H -1.753962 2.389459 1.159656

C 1.907863 0.834357 -1.153005

H 3.122067 2.852650 0.309668

H 2.016891 4.317022 1.944925

C 1.054246 -0.058004 -1.773889

C -0.317470 -0.084558 -1.449733

H 1.435426 -0.752971 -2.517112

H -0.976078 -0.798017 -1.943357

N -0.877767 0.719307 -0.570961

C 3.389981 0.736511 -1.524191

C 4.154592 -0.280037 -0.637974

C 4.593532 0.228809 0.765327

H 3.454923 -1.111069 -0.501893

C 6.472973 0.036551 -1.364723

C 5.676631 -2.120853 -0.683046

C 5.993204 -0.351909 1.061449

H 4.656353 1.319972 0.788229

H 3.868589 -0.075323 1.526020

H 7.246232 -0.424281 -1.988970

H 6.177505 0.971401 -1.841450

C 6.994864 0.289208 0.083256

C 6.001912 -1.891258 0.830193

H 6.548060 -2.534910 -1.200515

H 4.861502 -2.837171 -0.817363

H 6.277730 -0.133349 2.097257

H 7.991972 -0.143800 0.229130

H 7.079361 1.363192 0.283835

H 7.030996 -2.229015 1.025962

O 4.076157 1.989101 -1.520751

H 3.633503 2.571085 -2.152723

C 5.127058 -2.603717 1.836196

H 5.388622 -2.379323 2.872549

C 4.115333 -3.446751 1.625374

H 3.781316 -3.740355 0.634945

H 3.576694 -3.888349 2.458253

N 5.304302 -0.868674 -1.367976

H 3.433144 0.304242 -2.533567

P -3.818543 -0.405911 -0.644035

C -4.761910 1.131513 -0.034702

F -5.745978 1.393367 -0.920564

F -3.944153 2.200471 -0.004275

F -5.319800 1.011494 1.181220

C -3.200897 -1.025543 1.054846

F -2.716558 -0.005019 1.784178

F -2.204806 -1.907992 0.849545

F -4.139550 -1.641771 1.796588

C -5.341035 -1.546812 -0.773737

F -4.910809 -2.824669 -0.691260

F -5.897761 -1.375826 -1.989874

F -6.307968 -1.375133 0.141146

H6C2PHCD1COMPLEXOPT.LOG.out

xyz generated by gcpd3 webservice:

C 2.952569 -2.417950 -2.211647

C 2.956242 -2.908526 -0.927648

C 2.199949 -2.277302 0.096447

C 1.414295 -1.112301 -0.216117

C 1.446098 -0.627941 -1.557456

C 2.192457 -1.265913 -2.524119

H 3.533008 -2.909453 -2.987110

H 3.529062 -3.786518 -0.648050

C 0.660826 -0.518461 0.851115

H 0.884464 0.264083 -1.797148

H 2.195475 -0.879949 -3.539583

C 0.764591 -1.105031 2.098792

C 1.566035 -2.250151 2.295090

H 0.225422 -0.685483 2.944188

H 1.630026 -2.696069 3.287394

N 2.266257 -2.830891 1.343183

C -0.277582 0.689051 0.728650

C -1.742376 0.252837 0.462077

C -2.076884 -0.116181 -1.013053

H -1.892456 -0.634404 1.086326

C -2.828551 2.419474 0.091877

C -4.023351 0.594677 1.088796

C -3.463511 0.470919 -1.349463

H -1.341655 0.309756 -1.700392

H -2.065326 -1.201997 -1.147769

H -3.506740 3.131440 0.575127

H -1.848911 2.890434 0.015951

C -3.357070 2.006738 -1.316945

C -4.511191 0.030062 -0.286622

H -4.734601 1.334659 1.470284

H -3.951042 -0.192356 1.844581

H -3.782440 0.132640 -2.342552

H -4.336260 2.456087 -1.523333

H -2.673612 2.348827 -2.102171

H -5.455985 0.526039 -0.557563

O 0.125307 1.648586 -0.237414

H 0.989892 2.013871 0.035752

C -4.782265 -1.454938 -0.360791

H -5.072729 -1.792673 -1.358095

C -4.724590 -2.371836 0.606082

H -4.454539 -2.139871 1.631731

H -4.957222 -3.412266 0.400058

N -2.707033 1.250201 0.987854

H -0.308542 1.157356 1.723131

P 3.378437 2.514448 0.416284

H 3.956013 3.751255 0.816035

C 4.273791 1.398130 1.608282

H 3.918020 1.593641 2.623580

H 4.038851 0.358178 1.366353

H 5.358394 1.533649 1.578820

C 4.350705 2.249977 -1.150182

H 4.096320 1.268081 -1.558599

H 4.061089 3.004889 -1.886521

H 5.431328 2.303576 -0.991613

H6C2PHCD2COMPLEXOPT.LOG.out

xyz generated by gcpd3 webservice:

C -2.288349 2.458748 1.886727

C -2.810865 1.499730 1.051923

C -2.005351 0.889403 0.053318

C -0.625357 1.277194 -0.083707

C -0.127048 2.283705 0.794424

C -0.936622 2.852672 1.751929

H -2.912538 2.919042 2.646987

H -3.845498 1.180277 1.123296

C 0.142709 0.622985 -1.103710

H 0.898207 2.608637 0.686073

H -0.531925 3.616944 2.409199

C -0.505820 -0.313266 -1.886709

C -1.864847 -0.621279 -1.663878

H 0.029273 -0.823754 -2.683216

H -2.355913 -1.366063 -2.289385

N -2.602714 -0.053838 -0.733400

C 1.631308 0.841664 -1.387509

C 2.535908 -0.135470 -0.595136

C 2.797214 0.229930 0.895236

H 2.005631 -1.092798 -0.629335

C 4.780041 0.713735 -1.097067

C 4.392836 -1.629829 -0.770065

C 4.269491 -0.105857 1.213157

H 2.635239 1.295782 1.074847

H 2.114327 -0.321170 1.548433

H 5.660257 0.497604 -1.712406

H 4.332792 1.634291 -1.472146

C 5.165772 0.853001 0.407901

C 4.599377 -1.563843 0.779491

H 5.350553 -1.794664 -1.274381

H 3.740214 -2.459318 -1.055905

H 4.456302 0.009743 2.287207

H 6.222063 0.608032 0.573054

H 5.016852 1.882214 0.753258

H 5.665985 -1.720612 1.001712

O 2.073517 2.183492 -1.175803

H 1.538951 2.762256 -1.735553

C 3.843381 -2.570748 1.615939

H 3.998344 -2.444913 2.689716

C 3.045506 -3.563992 1.221509

H 2.832742 -3.778253 0.178688

H 2.570735 -4.217474 1.947096

N 3.813723 -0.384684 -1.308064

H 1.793629 0.570841 -2.440249

P -6.532553 -0.584825 0.303876

H -5.416301 -0.540156 -0.575758

C -7.873406 -0.684560 -0.997243

H -8.836763 -0.861494 -0.509602

H -7.939322 0.273422 -1.521135

H -7.700734 -1.477107 -1.731498

C -6.387365 -2.388914 0.775387

H -5.488511 -2.527659 1.382741

H -7.249365 -2.674189 1.385825

H -6.333210 -3.055423 -0.090525

H9C3PCD1COMPLEXOPT.LOG.out

xyz generated by gcpd3 webservice:

C 2.339477 -3.128980 -2.116471

C 2.267709 -3.569164 -0.816145

C 1.625091 -2.789777 0.183163

C 1.035689 -1.525859 -0.172059

C 1.139444 -1.100436 -1.529334

C 1.771752 -1.882182 -2.471031

H 2.832429 -3.733397 -2.872534

H 2.693832 -4.517042 -0.504474

C 0.394730 -0.779766 0.872079

H 0.727704 -0.138638 -1.801304

H 1.835047 -1.536653 -3.499068

C 0.410703 -1.328543 2.140812

C 1.016021 -2.581193 2.378863

H -0.048611 -0.796122 2.969686

H 1.011704 -2.995946 3.386698

N 1.606086 -3.302486 1.448677

C -0.342223 0.555551 0.703396

C -1.858686 0.342792 0.455023

C -2.253351 -0.046263 -0.999523

H -2.143580 -0.475851 1.124512

C -2.601734 2.625741 -0.041124

C -4.054404 1.068020 1.059846

C -3.541635 0.718981 -1.366715

H -1.467819 0.232676 -1.706372

H -2.400530 -1.127495 -1.080275

H -3.151488 3.463622 0.401953

H -1.560618 2.928409 -0.149558

C -3.209461 2.221540 -1.420226

C -4.631714 0.498023 -0.278103

H -4.641771 1.931421 1.389625

H -4.100059 0.330195 1.865634

H -3.917382 0.376889 -2.338364

H -4.115101 2.799500 -1.642502

H -2.496778 2.416623 -2.229410

H -5.497903 1.107924 -0.577867

O 0.198354 1.402564 -0.298853

H 1.118089 1.629448 -0.049241

C -5.114312 -0.934086 -0.262894

H -5.474716 -1.279417 -1.234454

C -5.161067 -1.797573 0.752477

H -4.833610 -1.552514 1.758166

H -5.543151 -2.803852 0.609411

N -2.653538 1.506285 0.922485

H -0.292595 1.060084 1.679519

P 3.511389 1.973827 0.202642

C 4.331057 2.936080 1.567166

H 3.953911 3.962937 1.569375

H 4.081591 2.486403 2.532637

H 5.421198 2.960183 1.461031

C 4.272091 2.779709 -1.291327

H 3.983890 2.224874 -2.188819

H 3.892086 3.800478 -1.392401

H 5.365587 2.812936 -1.233199

C 4.476002 0.387128 0.264030

H 4.236759 -0.151441 1.185264

H 4.182322 -0.249028 -0.575424

H 5.556963 0.559825 0.223382

H9C3PHCD2COMPLEXOPT.LOG.out

xyz generated by gcpd3 webservice:

C -1.455017 3.561599 0.570317

C -2.251989 2.450580 0.423864

C -1.727134 1.235298 -0.088789

C -0.342891 1.170381 -0.470867

C 0.451922 2.338719 -0.294023

C -0.090408 3.501127 0.213004

H -1.870152 4.484729 0.964014

H -3.303669 2.467567 0.686229

C 0.142487 -0.068564 -1.006516

H 1.506183 2.315128 -0.543536

H 0.538626 4.376713 0.339147

C -0.753740 -1.112143 -1.103867

C -2.092133 -0.951258 -0.684643

H -0.436849 -2.068680 -1.508599

H -2.789320 -1.782024 -0.775035

N -2.578406 0.169413 -0.191636

C 1.574099 -0.306492 -1.471832

C 2.604393 -0.441285 -0.295216

C 3.517352 0.791765 -0.030715

H 2.021801 -0.654278 0.607585

C 4.417654 -1.430477 -1.579655

C 4.128865 -1.976213 0.742663

C 4.969676 0.299728 0.137229

H 3.478884 1.480983 -0.880343

H 3.188316 1.328158 0.863814

H 4.925224 -2.383302 -1.763761

H 3.882412 -1.168343 -2.495578

C 5.433725 -0.311092 -1.197288

C 5.038440 -0.802341 1.233142

H 4.727543 -2.876058 0.567128

H 3.374681 -2.240525 1.488935

H 5.616107 1.139817 0.417155

H 6.449697 -0.711448 -1.093255

H 5.470035 0.462937 -1.971803

H 6.082621 -1.150293 1.261547

O 1.984601 0.603636 -2.501695

H 1.438501 1.400508 -2.456964

C 4.748828 -0.237183 2.604565

H 5.402539 0.594814 2.875816

C 3.832287 -0.616036 3.496357

H 3.139521 -1.434841 3.327517

H 3.745637 -0.110593 4.453621

N 3.426998 -1.654888 -0.510416

H 1.576654 -1.295028 -1.942197

P -6.217053 -0.942736 -0.159451

C -7.999949 -1.487677 -0.143680

H -8.583314 -0.867471 -0.831160

H -8.060303 -2.520755 -0.500023

H -8.457323 -1.433112 0.852221

C -6.312815 0.693315 0.723203

H -5.282500 0.977205 0.965159

H -6.735210 1.456920 0.063484

H -6.903769 0.650801 1.645632

C -5.571359 -1.987203 1.245014

H -5.611418 -3.045191 0.966622

H -4.526028 -1.722837 1.429943

H -6.140035 -1.848500 2.172768

cl2phcd1optOUTPUT.LOG.out

xyz generated by gcpd3 webservice:

C 2.437968 -2.234263 -2.351665

C 2.273840 -3.013119 -1.230517

C 1.461688 -2.575280 -0.148911

C 0.801163 -1.299023 -0.228441

C 0.995665 -0.520651 -1.409242

C 1.791163 -0.978340 -2.438258

H 3.061379 -2.578496 -3.171421

H 2.751392 -3.981794 -1.127557

C 0.008122 -0.897490 0.897909

H 0.515563 0.445105 -1.480387

H 1.922626 -0.366258 -3.325518

C -0.058702 -1.770036 1.967874

C 0.616103 -3.009496 1.932467

H -0.633467 -1.505688 2.851507

H 0.543171 -3.684485 2.784409

N 1.357033 -3.414167 0.922396

C -0.791967 0.403335 1.015867

C -2.260411 0.233787 0.548385

C -2.495628 0.281642 -0.990386

H -2.556765 -0.754675 0.915418

C -3.086526 2.538783 0.667499

C -4.540258 0.681223 1.106589

C -3.787730 1.085138 -1.247671

H -1.667543 0.779442 -1.501418

H -2.573674 -0.731219 -1.396964

H -3.722054 3.190032 1.277231

H -2.060421 2.893557 0.763270

C -3.541485 2.544848 -0.824604

C -4.956710 0.516747 -0.392466

H -5.204326 1.389932 1.611845

H -4.613778 -0.263707 1.651821

H -4.052074 1.036278 -2.310527

H -4.456604 3.134007 -0.959626

H -2.772705 2.994114 -1.463173

H -5.827129 1.160863 -0.590350

O -0.198488 1.513896 0.345547

H 0.691743 1.643174 0.703159

C -5.348952 -0.870468 -0.846691

H -5.570694 -0.929985 -1.914439

C -5.467006 -1.989287 -0.130387

H -5.280013 -2.035201 0.938079

H -5.770549 -2.920251 -0.599516

N -3.160553 1.181994 1.249375

P 3.560687 1.027233 0.256956

H 3.925026 -0.273940 -0.179648

Cl 5.005427 1.142396 1.772483

Cl 4.448768 2.038190 -1.347224

H -0.870183 0.626479 2.089195

cl2phcd2optoutput.LOG.out

xyz generated by gcpd3 webservice:

C -1.761737 2.769299 1.946607

C -2.368660 1.761469 1.235205

C -1.650283 1.038869 0.246974

C -0.272839 1.365677 -0.014410

C 0.313086 2.425167 0.737537

C -0.411467 3.101833 1.692705

H -2.321381 3.316907 2.698892

H -3.408746 1.503014 1.397784

C 0.410443 0.604322 -1.019415

H 1.336734 2.703425 0.531152

H 0.058295 3.904966 2.252657

C -0.310456 -0.373394 -1.679546

C -1.656080 -0.623047 -1.346538

H 0.157502 -0.965600 -2.460800

H -2.211979 -1.401249 -1.866251

N -2.312480 0.044580 -0.419197

C 1.885591 0.745706 -1.405586

C 2.801429 -0.194516 -0.579476

C 3.187539 0.307236 0.842158

H 2.229132 -1.122407 -0.476414

C 5.030501 0.489471 -1.334877

C 4.578640 -1.784892 -0.719987

C 4.669086 -0.050326 1.083814

H 3.072449 1.390940 0.924611

H 2.542337 -0.151059 1.597461

H 5.853719 0.162694 -1.979209

H 4.595021 1.382354 -1.783784

C 5.530005 0.780054 0.113886

C 4.916879 -1.558413 0.790954

H 5.481763 -2.054199 -1.277030

H 3.868910 -2.605303 -0.856763

H 4.942221 0.172808 2.121736

H 6.587947 0.516111 0.230634

H 5.436824 1.845962 0.350075

H 5.992998 -1.732250 0.942711

O 2.381225 2.082955 -1.336644

H 1.866935 2.623062 -1.951443

C 4.203261 -2.439004 1.791174

H 4.444396 -2.199009 2.828942

C 3.354982 -3.444977 1.573555

H 3.058069 -3.770552 0.581246

H 2.924239 -3.997845 2.402813

N 4.008745 -0.579107 -1.350258

P -4.810480 -1.088630 -0.216155

Cl -5.709937 0.803491 -0.461223

Cl -6.497971 -2.282226 0.325390

H -4.416016 -0.879147 1.130742

H 1.977097 0.379166 -2.437346

cl3pcd1optoutput.LOG.out

xyz generated by gcpd3 webservice:

C 3.200666 -3.965422 2.006127

C 3.387220 -4.122550 0.653469

C 2.750478 -3.257121 -0.275642

C 1.902631 -2.198259 0.205721

C 1.728865 -2.074601 1.615422

C 2.361820 -2.932930 2.486627

H 3.692272 -4.632822 2.707784

H 4.019684 -4.904148 0.246056

C 1.290059 -1.350387 -0.776694

H 1.079205 -1.299090 1.997024

H 2.213062 -2.815779 3.556113

C 1.547934 -1.628182 -2.105800

C 2.402711 -2.692401 -2.466750

H 1.093738 -1.025360 -2.887526

H 2.596802 -2.890470 -3.520348

N 2.991841 -3.487577 -1.599269

C 0.397241 -0.142764 -0.488251

C 1.207601 1.170808 -0.342971

C 1.946680 1.389806 1.007824

H 1.958189 1.108367 -1.138108

C -0.451769 2.791138 0.446733

C 1.314669 3.453713 -1.032564

C 1.880861 2.895842 1.341889

H 1.467025 0.831933 1.816575

H 2.981693 1.042354 0.938874

H -1.095765 3.601858 0.089711

H -1.099141 1.960566 0.727026

C 0.416441 3.252167 1.656949

C 2.333792 3.745440 0.118462

H 0.711655 4.343470 -1.240472

H 1.821718 3.193056 -1.965390

H 2.522426 3.117155 2.202710

H 0.320615 4.331064 1.829483

H 0.096796 2.751174 2.577649

H 2.234680 4.799482 0.419440

O -0.467702 -0.322729 0.642349

H -0.847260 -1.210051 0.585907

C 3.798239 3.532544 -0.189949

H 4.452417 3.722767 0.663685

C 4.372851 3.162059 -1.335115

H 3.813720 2.957331 -2.243005

H 5.450939 3.055252 -1.407152

N 0.388548 2.357876 -0.689486

P -3.780500 -0.660955 0.134236

Cl -5.650375 -1.594875 0.020909

Cl -3.564706 0.046141 -1.818705

Cl -4.267917 1.088577 1.159101

H -0.221051 0.015217 -1.382199

cl3pcd2optoutput.out.out

xyz generated by gcpd3 webservice:

C -1.231647 2.987294 1.965108

C -1.883204 1.943415 1.352464

C -1.226297 1.149120 0.376205

C 0.141746 1.434890 0.030857

C 0.775277 2.534968 0.679239

C 0.106617 3.285273 1.620058

H -1.744236 3.587326 2.711068

H -2.909763 1.693855 1.595984

C 0.766628 0.592101 -0.946907

H 1.791151 2.783380 0.406412

H 0.612655 4.117495 2.100341

C 0.001781 -0.417802 -1.499579

C -1.333669 -0.614707 -1.094695

H 0.425414 -1.074381 -2.254377

H -1.921283 -1.416920 -1.536403

N -1.938502 0.130216 -0.192447

C 2.223254 0.680720 -1.410986

C 3.166173 -0.211864 -0.563748

C 3.615691 0.378461 0.804550

H 2.591153 -1.124704 -0.376039

C 5.367459 0.396648 -1.454272

C 4.924204 -1.826211 -0.670514

C 5.103490 0.023860 1.008246

H 3.511622 1.466340 0.819734

H 2.999501 -0.023103 1.614296

H 6.161518 0.017529 -2.106576

H 4.921172 1.261168 -1.945841

C 5.927553 0.780493 -0.049937

C 5.329300 -1.502946 0.805572

H 5.800667 -2.141677 -1.245591

H 4.202626 -2.645996 -0.721449

H 5.421614 0.312548 2.016792

H 6.988338 0.517520 0.041137

H 5.848527 1.860518 0.117526

H 6.410086 -1.675305 0.921913

O 2.737960 2.011881 -1.462485

H 2.196748 2.515288 -2.085217

C 4.655559 -2.307948 1.893285

H 4.956193 -2.011104 2.900398

C 3.776872 -3.305135 1.782162

H 3.419853 -3.681821 0.828526

H 3.379963 -3.797040 2.664982

N 4.337756 -0.659293 -1.356303

P -4.814655 -0.327427 0.312122

Cl -4.289638 -2.342189 0.038439

Cl -5.026311 0.310156 -1.675230

Cl -6.863029 -0.571770 0.815957

H 2.259745 0.239853 -2.416780

c2n2phcd1complexoptoutput.LOG.out

xyz generated by gcpd3 webservice:

C 4.506449 -1.631512 2.019411

C 4.757592 -1.758104 0.673459

C 3.732763 -1.528651 -0.282562

C 2.419611 -1.154347 0.163959

C 2.194082 -1.047242 1.566828

C 3.212033 -1.276633 2.466174

H 5.296933 -1.808790 2.742582

H 5.735160 -2.035838 0.293804

C 1.424912 -0.921117 -0.843606

H 1.202111 -0.804111 1.926588

H 3.016809 -1.188936 3.530986

C 1.793041 -1.097232 -2.160735

C 3.116219 -1.472239 -2.488346

H 1.072693 -0.940686 -2.957872

H 3.392280 -1.602759 -3.533996

N 4.063126 -1.680935 -1.599044

C 0.005516 -0.472835 -0.540747

C -0.085259 0.990833 -0.058909

C -1.523629 1.428487 0.349844

H 0.564035 1.074151 0.817074

C -0.463766 2.153675 -2.176063

C 0.733793 3.206906 -0.388072

C -1.739526 2.872487 -0.151090

H -2.279334 0.779783 -0.108137

H -1.647685 1.362484 1.434659

H 0.054799 2.767747 -2.919502

H -0.681630 1.197974 -2.660320

C -1.775556 2.842017 -1.689575

C -0.553623 3.779578 0.293325

H 1.108493 3.907024 -1.141535

H 1.542612 3.057607 0.331616

H -2.679763 3.272642 0.245203

H -1.857466 3.861201 -2.085270

H -2.657500 2.291859 -2.035724

H -0.757491 4.779142 -0.119198

O -0.615357 -1.290322 0.481798

H -0.242340 -2.178976 0.402037

C -0.519833 3.943889 1.795727

H -1.449604 4.341677 2.208143

C 0.469640 3.679162 2.650006

H 1.434841 3.289293 2.341692

H 0.346970 3.857010 3.714122

N 0.476644 1.920716 -1.061199

H -0.583110 -0.583416 -1.460398

P -3.259657 -2.356704 0.784605

H -3.295973 -1.062657 1.353353

C -5.029047 -2.680956 0.940816

N -6.132424 -3.002913 1.119468

C -3.265449 -1.824977 -0.933036

N -3.161332 -1.543642 -2.056969

c2n2phcd2complexoptoutput.LOG.out

xyz generated by gcpd3 webservice:

C -2.146914 2.943969 1.856954

C -2.768191 1.949840 1.139000

C -2.060138 1.207381 0.157478

C -0.672047 1.501089 -0.085565

C -0.069103 2.547396 0.671482

C -0.786640 3.244290 1.617260

H -2.700665 3.503842 2.604819

H -3.813701 1.709645 1.296238

C 0.004654 0.722425 -1.081619

H 0.963455 2.799830 0.476003

H -0.303299 4.036364 2.181507

C -0.735809 -0.231278 -1.753282

C -2.093796 -0.444307 -1.440132

H -0.275500 -0.835517 -2.529965

H -2.659030 -1.200489 -1.980317

N -2.746392 0.235226 -0.517851

C 1.488689 0.821160 -1.444968

C 2.366824 -0.138336 -0.601013

C 2.742557 0.357768 0.825545

H 1.769439 -1.050874 -0.504505

C 4.623943 0.486632 -1.324169

C 4.106444 -1.772759 -0.706198

C 4.211694 -0.032163 1.090898

H 2.650399 1.443945 0.904342

H 2.075869 -0.084817 1.571351

H 5.449620 0.135832 -1.952697

H 4.217976 1.387199 -1.785195

C 5.106567 0.773676 0.131015

C 4.429390 -1.546945 0.808213

H 5.010039 -2.068570 -1.249024

H 3.377330 -2.574903 -0.848613

H 4.474153 0.189194 2.132022

H 6.156366 0.486560 0.266116

H 5.033574 1.842720 0.360380

H 5.499284 -1.744176 0.975217

O 2.019379 2.146082 -1.375604

H 1.521661 2.696915 -1.994430

C 3.682179 -2.406267 1.802493

H 3.918337 -2.170093 2.842306

C 2.808462 -3.388541 1.577845

H 2.511883 -3.707088 0.583203

H 2.351725 -3.925843 2.403329

N 3.576844 -0.556402 -1.350679

H 1.586349 0.447193 -2.473482

P -5.638920 -0.572285 0.284371

C -5.396813 -1.307292 -1.336683

N -5.260810 -1.684534 -2.428396

C -4.624274 -1.681991 1.267293

N -3.975969 -2.316063 1.995160

H -6.848275 -1.283329 0.541923

C3n3pcd1complexoptOUTPUT.LOG.out

xyz generated by gcpd3 webservice:

C 1.089992 -4.680636 1.655711

C 0.846131 -4.870268 0.315691

C 0.533939 -3.774971 -0.532414

C 0.470528 -2.448267 0.014658

C 0.741436 -2.294398 1.405176

C 1.039890 -3.378150 2.202265

H 1.326450 -5.525408 2.295302

H 0.882201 -5.853352 -0.141531

C 0.120698 -1.382347 -0.879984

H 0.750782 -1.308601 1.858850

H 1.244704 -3.227893 3.257910

C -0.106068 -1.707802 -2.199918

C 0.007393 -3.047350 -2.638842

H -0.377466 -0.937785 -2.915895

H -0.169418 -3.284398 -3.687211

N 0.306412 -4.055727 -1.849472

C -0.050450 0.064785 -0.444939

C 1.251107 0.782398 -0.019332

C 1.005047 2.235955 0.483257

H 1.705189 0.205281 0.791773

C 1.921177 1.738798 -2.167356

C 3.558207 1.119016 -0.532549

C 2.120645 3.127771 -0.098808

H 0.029144 2.609560 0.153797

H 1.003850 2.268132 1.576397

H 2.651745 1.606870 -2.972107

H 0.946254 1.486887 -2.593624

C 1.932280 3.200087 -1.625050

C 3.517162 2.504328 0.194708

H 4.289936 1.134837 -1.346570

H 3.857515 0.313957 0.143399

H 2.065987 4.131154 0.338374

H 2.742883 3.782451 -2.078690

H 0.994461 3.713438 -1.863742

H 4.259149 3.170375 -0.271304

O -1.062589 0.201501 0.578300

H -0.888321 -0.429750 1.289207

C 3.821591 2.506414 1.675420

H 3.722441 3.491120 2.136998

C 4.196242 1.494284 2.459254

H 4.335753 0.480058 2.097505

H 4.392334 1.653133 3.515308

N 2.249893 0.759943 -1.111330

H -0.474581 0.613976 -1.289239

P -3.778675 0.447508 0.167029

C -5.503882 0.975785 0.048707

N -6.644182 1.161337 -0.085774

C -3.068477 1.840836 -0.717755

N -2.561315 2.634765 -1.399175

C -3.504958 0.978393 1.860591

N -3.314798 1.167307 2.991794

C3n3pcd2complexoptOUTPUT.LOG.out

xyz generated by gcpd3 webservice:

C -1.726105 2.710895 1.980031

C -2.329193 1.713956 1.250448

C -1.613119 1.021239 0.240383

C -0.237836 1.363196 -0.017559

C 0.343363 2.411434 0.753426

C -0.381487 3.062556 1.725380

H -2.285761 3.232422 2.750283

H -3.360327 1.438510 1.432590

C 0.452296 0.629114 -1.037321

H 1.364071 2.700346 0.548120

H 0.084299 3.857393 2.300061

C -0.259269 -0.341907 -1.717421

C -1.602762 -0.604771 -1.393529

H 0.213455 -0.915886 -2.509070

H -2.148292 -1.375253 -1.936009

N -2.268506 0.039036 -0.454413

C 1.926314 0.790726 -1.421486

C 2.847172 -0.166150 -0.620762

C 3.208736 0.286073 0.823387

H 2.287064 -1.105157 -0.558922

C 5.079948 0.571755 -1.313054

C 4.641794 -1.731131 -0.805610

C 4.687805 -0.076544 1.073781

H 3.089131 1.365936 0.943061

H 2.553111 -0.200564 1.551453

H 5.913273 0.283466 -1.962707

H 4.641102 1.480901 -1.724148

C 5.560463 0.796858 0.153486

C 4.945768 -1.570158 0.721019

H 5.559738 -1.962101 -1.355525

H 3.947001 -2.553922 -0.994871

H 4.943383 0.102937 2.124451

H 6.616950 0.528014 0.272103

H 5.464024 1.851109 0.436196

H 6.018598 -1.749078 0.889122

O 2.413396 2.127913 -1.317531

H 1.906123 2.679469 -1.927983

C 4.210957 -2.493983 1.665597

H 4.422493 -2.294758 2.718285

C 3.375265 -3.494739 1.384609

H 3.107707 -3.781381 0.372112

H 2.925361 -4.082694 2.178740

N 4.068351 -0.504033 -1.391136

H 2.019100 0.452169 -2.462683

P -4.773564 -1.066555 -0.515985

C -4.499543 -1.204831 1.254407

N -4.250047 -1.388309 2.375014

C -6.464837 -1.739785 -0.496068

N -7.488523 -2.279438 -0.615784

C -5.264378 0.658638 -0.624176

N -5.546049 1.771103 -0.810601

F2PHCD1COMPLEXOPT.LOG.out

xyz generated by gcpd3 webservice:

C -4.273217 -0.792237 -1.838240

C -4.633530 0.290531 -1.072240

C -3.748595 0.817369 -0.094187

C -2.456131 0.211762 0.095798

C -2.123978 -0.914358 -0.712821

C -3.008106 -1.396474 -1.652250

H -4.956855 -1.187601 -2.583780

H -5.595202 0.780440 -1.182130

C -1.603695 0.790159 1.094258

H -1.169982 -1.402967 -0.580042

H -2.728164 -2.254461 -2.256446

C -2.094491 1.870202 1.803496

C -3.378515 2.388866 1.529369

H -1.492141 2.332830 2.580787

H -3.743871 3.244409 2.096489

N -4.189698 1.894961 0.618689

C -0.180329 0.343320 1.430911

C 0.890767 1.067323 0.577499

C 1.114513 0.531753 -0.866702

H 0.524725 2.097300 0.511255

C 2.952836 -0.079976 1.240474

C 2.967036 2.248036 0.673873

C 2.629923 0.570494 -1.155681

H 0.770797 -0.498970 -0.972011

H 0.559370 1.139142 -1.587567

H 3.853991 0.059784 1.847271

H 2.363387 -0.867002 1.713068

C 3.316726 -0.451011 -0.230695

C 3.208457 1.982147 -0.848706

H 3.921758 2.315825 1.205355

H 2.445598 3.194348 0.840464

H 2.815171 0.315803 -2.205472

H 4.401940 -0.435692 -0.387519

H 2.968528 -1.459397 -0.478078

H 4.292527 1.919431 -1.029050

O -0.000265 -1.080860 1.373928

H -0.704922 -1.483521 1.899318

C 2.680214 3.016208 -1.816327

H 2.837914 2.749657 -2.863565

C 2.075210 4.177017 -1.560901

H 1.879027 4.538386 -0.556103

H 1.753257 4.825833 -2.369772

N 2.177810 1.175610 1.308448

H 0.020171 0.676737 2.457997

P 0.781660 -3.755405 0.483996

H 2.030356 -3.217897 0.935634

F 1.359121 -5.191652 0.030592

F 0.785599 -3.022265 -0.959369

F2PHCD2COMPLEXOPT.LOG.out

xyz generated by gcpd3 webservice:

C 2.388999 2.917680 -1.623182

C 2.968082 1.805161 -1.060760

C 2.236372 0.979730 -0.167522

C 0.872535 1.307748 0.151462

C 0.313570 2.475151 -0.445406

C 1.051535 3.252756 -1.309411

H 2.958198 3.543955 -2.303617

H 3.995193 1.529577 -1.272685

C 0.178393 0.436578 1.054897

H -0.700197 2.752396 -0.193147

H 0.602252 4.137419 -1.750837

C 0.878860 -0.639362 1.567456

C 2.215321 -0.878622 1.189059

H 0.401106 -1.318364 2.268311

H 2.760135 -1.728697 1.591340

N 2.878416 -0.109360 0.350109

C -1.287482 0.565743 1.477090

C -2.242243 -0.222331 0.543207

C -2.639341 0.485074 -0.785837

H -1.698658 -1.140905 0.298854

C -4.441298 0.403411 1.424705

C -4.057354 -1.775834 0.499113

C -4.131005 0.190045 -1.050642

H -2.504587 1.567404 -0.713168

H -2.013755 0.129052 -1.609596

H -5.260328 0.014429 2.039234

H -3.973149 1.218973 1.976368

C -4.961348 0.893896 0.038500

C -4.403946 -1.339827 -0.962792

H -4.963090 -2.091666 1.026702

H -3.371147 -2.626775 0.515175

H -4.414069 0.558297 -2.043571

H -6.025475 0.662877 -0.092293

H -4.855042 1.980623 -0.053946

H -5.484427 -1.472269 -1.125112

O -1.741395 1.914345 1.601694

H -1.197152 2.349611 2.271364

C -3.715762 -2.086203 -2.082765

H -3.978725 -1.714704 -3.075541

C -2.862647 -3.109072 -2.014277

H -2.543155 -3.556869 -1.078277

H -2.449667 -3.549841 -2.916529

N -3.447756 -0.680825 1.276660

H -1.379606 0.061892 2.449107

P 5.490242 -0.814932 -0.096858

F 6.861399 -1.560441 -0.558585

F 5.003250 -1.948511 0.958413

H 4.708986 -1.255660 -1.212024

F3PCD1COMPLEXOPT.LOG.out

xyz generated by gcpd3 webservice:

C -4.304122 -0.468037 -1.936949

C -4.646174 0.567068 -1.100022

C -3.752177 1.013471 -0.090657

C -2.467865 0.378374 0.053499

C -2.155771 -0.698485 -0.827399

C -3.049504 -1.104759 -1.793128

H -4.995388 -0.802653 -2.704876

H -5.600625 1.077405 -1.174064

C -1.604124 0.877337 1.084518

H -1.213120 -1.215164 -0.721387

H -2.786756 -1.928909 -2.449811

C -2.080162 1.910157 1.870249

C -3.358570 2.461791 1.638250

H -1.469009 2.311275 2.674386

H -3.712039 3.279379 2.265724

N -4.177523 2.044502 0.696523

C -0.180297 0.400542 1.376364

C 0.884684 1.197132 0.578647

C 1.126901 0.741618 -0.890869

H 0.505255 2.224064 0.567659

C 2.945016 0.030655 1.215592

C 2.954603 2.381187 0.745422

C 2.646262 0.779470 -1.155942

H 0.770023 -0.277262 -1.054721

H 0.589758 1.394215 -1.585299

H 3.844382 0.154337 1.828502

H 2.360883 -0.778585 1.655993

C 3.314857 -0.285010 -0.266932

C 3.230229 2.171538 -0.780475

H 3.897052 2.441805 1.299252

H 2.416816 3.314259 0.933196

H 2.844944 0.569775 -2.213354

H 4.401155 -0.277111 -0.416680

H 2.957704 -1.279426 -0.555723

H 4.317808 2.104389 -0.937309

O 0.008749 -1.006235 1.185525

H -0.700275 -1.476527 1.644323

C 2.736307 3.248159 -1.719404

H 2.904281 3.014775 -2.772979

C 2.152306 4.412144 -1.431825

H 1.949584 4.742888 -0.417822

H 1.855999 5.095400 -2.221928

N 2.164169 1.279524 1.325575

H 0.024955 0.644366 2.427656

P 1.141423 -3.593247 0.319668

F 1.277345 -5.100384 -0.200157

F -0.363975 -3.713649 0.864376

F 0.796004 -2.934681 -1.099582

f3pcd2complexopt.log.out

xyz generated by gcpd3 webservice:

C -2.032367 3.058875 1.741815

C -2.659583 1.958341 1.208554

C -1.980420 1.109231 0.295464

C -0.619593 1.397375 -0.072276

C -0.010877 2.554977 0.495037

C -0.698835 3.357566 1.377268

H -2.559299 3.703073 2.439438

H -3.682495 1.701764 1.461913

C 0.018609 0.498482 -0.990049

H 1.000541 2.804690 0.207006

H -0.211708 4.233493 1.795481

C -0.727259 -0.563712 -1.467000

C -2.056541 -0.759691 -1.042213

H -0.291044 -1.262207 -2.175826

H -2.635419 -1.600436 -1.417679

N -2.669961 0.037501 -0.192837

C 1.471549 0.583460 -1.465881

C 2.433109 -0.235768 -0.568072

C 2.862093 0.438239 0.768110

H 1.879793 -1.150969 -0.332670

C 4.627823 0.373928 -1.475390

C 4.224976 -1.815366 -0.584063

C 4.354009 0.119943 1.000846

H 2.738965 1.523332 0.721075

H 2.246236 0.072275 1.594788

H 5.432587 -0.019405 -2.105840

H 4.166122 1.204643 -2.009113

C 5.173850 0.835048 -0.088916

C 4.606430 -1.411279 0.878252

H 5.115033 -2.136941 -1.134384

H 3.523835 -2.654011 -0.601925

H 4.659163 0.465007 1.995601

H 6.237719 0.592303 0.021074

H 5.078773 1.920866 0.024353

H 5.688348 -1.559391 1.015733

O 1.961820 1.917711 -1.604216

H 1.405767 2.372173 -2.251017

C 3.932416 -2.171061 1.997850

H 4.218484 -1.820493 2.991859

C 3.067726 -3.184042 1.927597

H 2.725281 -3.611397 0.990145

H 2.667754 -3.637029 2.829654

N 3.619276 -0.696927 -1.330877

H 1.513128 0.080214 -2.441855

P -5.335564 -0.754230 0.222936

F -6.799863 -1.426724 0.243676

F -5.248237 -0.607028 -1.370598

F -4.523739 -2.133036 0.339840

o2h2phcd1complexoptOUTPUT.out.out

xyz generated by gcpd3 webservice:

C -4.006520 -1.965617 -1.827096

C -4.643018 -1.039710 -1.035684

C -3.925314 -0.301412 -0.057405

C -2.511307 -0.521886 0.105773

C -1.892065 -1.497694 -0.727882

C -2.620363 -2.194401 -1.666346

H -4.563892 -2.524683 -2.572866

H -5.705063 -0.837356 -1.124674

C -1.839981 0.259258 1.104412

H -0.839069 -1.709627 -0.619651

H -2.119929 -2.930424 -2.288388

C -2.603486 1.146292 1.839986

C -3.986343 1.285526 1.592112

H -2.142716 1.750950 2.616369

H -4.568205 1.994450 2.180112

N -4.640009 0.596724 0.681629

C -0.342796 0.242085 1.415433

C 0.465333 1.246238 0.557181

C 0.807501 0.828996 -0.902748

H -0.184368 2.126567 0.513174

C 2.797601 0.757170 1.134148

C 2.083451 2.998185 0.671251

C 2.220806 1.364742 -1.222345

H 0.813243 -0.254802 -1.035254

H 0.063956 1.234784 -1.594815

H 3.621446 1.126443 1.754665

H 2.479458 -0.197131 1.557737

C 3.236657 0.598811 -0.353864

C 2.319558 2.877704 -0.870488

H 2.999564 3.325003 1.173683

H 1.315006 3.739298 0.906941

H 2.441916 1.216697 -2.285241

H 4.242954 1.005014 -0.513065

H 3.263415 -0.456051 -0.644280

H 3.353656 3.180756 -1.094043

O 0.244729 -1.073102 1.359867

H -0.273431 -1.658074 1.929024

C 1.433472 3.712851 -1.766938

H 1.626426 3.556436 -2.830541

C 0.478027 4.596457 -1.433363

H 0.214878 4.831488 -0.406342

H -0.077634 5.130244 -2.198565

N 1.678556 1.713147 1.273677

H -0.228093 0.612093 2.441848

P 2.368082 -3.910048 0.219156

O 4.000039 -3.558615 0.102828

H 4.317960 -3.256884 0.964067

H 2.366474 -4.726798 -0.942527

O 1.601096 -2.631216 -0.508872

H 1.196794 -2.054880 0.170749

O2H2PHCD2COMPLEXOPT.LOG.out

xyz generated by gcpd3 webservice:

C 2.547832 2.441061 -1.921334

C 3.057397 1.594748 -0.965138

C 2.213439 1.068918 0.048982

C 0.817822 1.416260 0.077294

C 0.336368 2.304666 -0.928148

C 1.179501 2.797929 -1.898329

H 3.197493 2.840768 -2.694118

H 4.103673 1.306826 -0.953317

C 0.015628 0.846554 1.120578

H -0.702981 2.599862 -0.906331

H 0.788600 3.473243 -2.653721

C 0.644757 0.020118 2.034224

C 2.019323 -0.263589 1.920571

H 0.081147 -0.422203 2.850700

H 2.502712 -0.919424 2.641780

N 2.784185 0.233219 0.969139

C -1.493264 1.039339 1.299039

C -2.315133 -0.058916 0.575601

C -2.524665 0.144082 -0.953711

H -1.745267 -0.981218 0.729993

C -4.607821 0.739382 0.919361

C -4.119346 -1.606523 0.815097

C -3.975835 -0.256837 -1.290251

H -2.376296 1.188753 -1.238492

H -1.807431 -0.456910 -1.520494

H -5.507129 0.530103 1.508490

H -4.220159 1.705864 1.241780

C -4.923895 0.746663 -0.608158

C -4.286277 -1.678895 -0.738833

H -5.081500 -1.773378 1.309947

H -3.437819 -2.377641 1.184509

H -4.124174 -0.242630 -2.376276

H -5.967173 0.468524 -0.799938

H -4.774878 1.746623 -1.030496

H -5.343259 -1.878330 -0.971937

O -1.965888 2.328852 0.909150

H -1.517629 2.987962 1.455763

C -3.489336 -2.737957 -1.465459

H -3.624941 -2.714232 -2.548839

C -2.676494 -3.670674 -0.967247

H -2.481009 -3.785657 0.094531

H -2.172272 -4.375339 -1.621557

N -3.609083 -0.296058 1.260443

H -1.706656 0.883144 2.365583

P 5.873474 -1.756148 0.331939

H 7.280806 -1.602842 0.464678

O 5.527123 -0.164181 0.582622

H 4.567505 -0.062095 0.811697

O 5.948314 -1.949099 -1.335565

H 5.144418 -2.400221 -1.624593

o3h3pcd1complexoptOUTPUT.LOG.out

xyz generated by gcpd3 webservice:

C -5.007948 -0.973951 -1.387851

C -4.873436 -1.515537 -0.131077

C -3.606320 -1.563457 0.508400

C -2.453200 -1.036256 -0.166908

C -2.629954 -0.492849 -1.472234

C -3.874633 -0.461411 -2.061705

H -5.981331 -0.941644 -1.868285

H -5.718557 -1.922733 0.413840

C -1.199133 -1.100887 0.527309

H -1.769028 -0.118620 -2.012198

H -3.985817 -0.041994 -3.057333

C -1.187076 -1.678010 1.779507

C -2.384524 -2.167098 2.350307

H -0.259890 -1.754885 2.338856

H -2.359743 -2.616717 3.342236

N -3.557099 -2.119291 1.755142

C 0.101411 -0.550352 -0.028444

C 0.153242 0.992682 -0.046719

C 1.366071 1.587099 -0.822602

H -0.765569 1.338260 -0.528926

C 1.421274 1.435347 1.996351

C -0.283733 2.945698 1.250545

C 1.864780 2.818169 -0.035396

H 2.184061 0.864667 -0.913396

H 1.068497 1.858571 -1.839093

H 1.286345 1.742676 3.038661

H 1.736531 0.388742 2.017658

C 2.487829 2.319260 1.280259

C 0.674516 3.758914 0.317316

H -0.265690 3.355805 2.265442

H -1.321252 2.994301 0.910383

H 2.606905 3.367371 -0.625838

H 2.786092 3.168743 1.906258

H 3.397923 1.744617 1.070837

H 1.099650 4.596386 0.891241

O 0.346111 -1.006443 -1.386879

H -0.043177 -1.888245 -1.473023

C 0.067862 4.367618 -0.926294

H 0.795673 4.889080 -1.551627

C -1.201290 4.356930 -1.336102

H -2.001872 3.873657 -0.784606

H -1.490938 4.851041 -2.258586

N 0.104251 1.525700 1.333192

H 0.917900 -0.924327 0.599376

P 3.332388 -2.667533 -0.521666

O 3.403378 -1.818016 0.909065

H 4.015787 -1.065451 0.834039

O 3.088467 -1.465629 -1.609954

H 2.127230 -1.269257 -1.678210

O 4.943316 -2.820936 -0.888000

H 5.259792 -3.685166 -0.592872

o3h3phcd2complexoptOUTPUT.out.out

xyz generated by gcpd3 webservice:

C -2.203828 -2.520521 -1.751114

C -2.718076 -1.571193 -0.899366

C -1.885324 -0.964042 0.077472

C -0.497564 -1.331249 0.174236

C -0.013363 -2.328523 -0.721736

C -0.845419 -2.902288 -1.656526

H -2.843872 -2.983435 -2.496319

H -3.755968 -1.257373 -0.954034

C 0.294277 -0.670117 1.170858

H 1.017936 -2.641774 -0.643451

H -0.452843 -3.660397 -2.327930

C -0.337093 0.254173 1.983453

C -1.704300 0.543978 1.814163

H 0.218915 0.767268 2.762909

H -2.194983 1.270658 2.458600

N -2.457184 -0.032566 0.898909

C 1.795978 -0.865933 1.398546

C 2.650514 0.128173 0.570837

C 2.874037 -0.243600 -0.924294

H 2.098804 1.073037 0.613832

C 4.928058 -0.663971 1.009552

C 4.475525 1.667410 0.676480

C 4.327279 0.124147 -1.289831

H 2.732301 -1.314413 -1.091167

H 2.158761 0.286534 -1.560020

H 5.821107 -0.421764 1.595750

H 4.515269 -1.591945 1.405423

C 5.270207 -0.806861 -0.505558

C 4.634792 1.593096 -0.878022

H 5.444204 1.857833 1.149614

H 3.813485 2.484179 0.976694

H 4.483241 0.003872 -2.368204

H 6.314721 -0.538773 -0.705166

H 5.135043 -1.842119 -0.838225

H 5.689655 1.773730 -1.135013

O 2.250913 -2.199211 1.168380

H 1.775371 -2.784244 1.773136

C 3.828018 2.574249 -1.697637

H 3.949551 2.441314 -2.774825

C 3.021805 3.553389 -1.285232

H 2.840500 3.774259 -0.237821

H 2.508579 4.189324 -2.000127

N 3.942737 0.413447 1.242102

H 1.993692 -0.591744 2.444093

P -5.770811 1.430899 -0.151130

O -5.161160 0.535231 1.051036

H -4.190878 0.335209 0.937064

O -5.597312 0.311562 -1.393074

H -5.510079 0.792127 -2.227996

O -7.360471 1.224150 0.275529

H -7.828124 2.053210 0.110405

o4n2phcd1optOUTPUT.LOG.out

xyz generated by gcpd3 webservice:

C 3.388916 -3.294182 2.213881

C 3.573162 -3.719399 0.918722

C 2.917314 -3.071935 -0.161146

C 2.046329 -1.961245 0.103439

C 1.893124 -1.543650 1.454792

C 2.544167 -2.192822 2.481374

H 3.894349 -3.797120 3.032602

H 4.220432 -4.555011 0.674590

C 1.386729 -1.356605 -1.016579

H 1.281093 -0.681507 1.694175

H 2.410760 -1.851234 3.503306

C 1.658233 -1.868256 -2.268121

C 2.553681 -2.953689 -2.419168

H 1.182734 -1.446687 -3.148689

H 2.758925 -3.342928 -3.415620

N 3.161410 -3.550405 -1.417181

C 0.400415 -0.201439 -0.904737

C 1.052308 1.173658 -0.523773

C 0.768187 1.721070 0.910233

H 2.133392 1.042390 -0.635186

C -0.713433 2.634726 -1.352286

C 1.582021 3.338825 -1.417550

C 0.420691 3.220160 0.796229

H -0.077308 1.208430 1.380690

H 1.643388 1.580806 1.551293

H -0.965294 3.295048 -2.188570

H -1.373830 1.766050 -1.429290

C -0.899655 3.355716 0.016538

C 1.537044 3.969295 0.012474

H 1.284284 4.076545 -2.169513

H 2.589524 3.007844 -1.683257

H 0.317812 3.652207 1.797934

H -1.143173 4.414937 -0.126125

H -1.723562 2.911973 0.585603

H 1.207572 5.015679 -0.071793

O -0.744265 -0.564386 -0.109938

H -0.478967 -0.892635 0.760309

C 2.833027 3.996198 0.789793

H 2.735585 4.451637 1.777507

C 4.038714 3.551607 0.432487

H 4.243348 3.090141 -0.528660

H 4.886562 3.644007 1.104363

N 0.678463 2.181241 -1.546451

P -3.293027 -0.834289 -0.486445

H -2.924601 -2.101664 0.013927

H -0.013778 -0.048628 -1.903071

N -3.137896 0.047974 1.170365

O -3.706939 1.131238 1.209527

O -2.475730 -0.452918 2.074162

N -5.118679 -1.290569 -0.275489

O -5.865272 -0.764536 0.528381

O -5.415639 -2.153377 -1.101903

o4n2phcd2optOUTPUT.LOG.out

xyz generated by gcpd3 webservice:

C -1.200704 3.157353 1.388469

C -1.965370 2.122221 0.901675

C -1.387371 1.120407 0.080629

C 0.009773 1.187933 -0.244511

C 0.764024 2.271440 0.285938

C 0.175453 3.231034 1.079588

H -1.654154 3.916272 2.018214

H -3.019504 2.046325 1.139508

C 0.561457 0.162155 -1.080631

H 1.827331 2.332560 0.090162

H 0.774827 4.044244 1.476774

C -0.284569 -0.848619 -1.490231

C -1.640968 -0.846596 -1.108964

H 0.082526 -1.653065 -2.118761

H -2.297565 -1.654576 -1.420989

N -2.185454 0.100481 -0.368071

C 2.013501 0.127115 -1.544277

C 3.026049 -0.251917 -0.401449

C 3.946676 0.896635 0.111164

H 2.436255 -0.632161 0.439499

C 4.848166 -0.970982 -1.845462

C 4.528576 -1.986727 0.310003

C 5.390418 0.359622 0.200647

H 3.931403 1.742915 -0.583595

H 3.610967 1.250947 1.090543

H 5.345718 -1.868827 -2.226691

H 4.327653 -0.510379 -2.689362

C 5.872968 0.024421 -1.222123

C 5.424956 -0.942569 1.052119

H 5.136321 -2.823915 -0.047994

H 3.767971 -2.410521 0.971216

H 6.040271 1.115975 0.655686

H 6.877735 -0.412710 -1.184174

H 5.942975 0.938399 -1.822107

H 6.466941 -1.296019 1.037523

O 2.371710 1.280653 -2.305549

H 1.941725 2.060268 -1.933470

C 5.095631 -0.662333 2.500514

H 5.762430 0.069105 2.962096

C 4.128743 -1.179711 3.259777

H 3.419013 -1.918483 2.900438

H 4.016981 -0.880385 4.297452

N 3.840847 -1.404607 -0.855753

P -4.724888 0.144956 -0.190751

H -4.527410 -0.435702 -1.460964

N -6.580158 -0.306040 -0.320116

O -7.135326 -1.079656 0.443955

O -7.120010 0.342338 -1.217628

H 2.083593 -0.703712 -2.250723

N -4.327186 -1.404839 0.803509

O -4.020426 -2.442872 0.226734

O -4.408633 -1.251858 2.015831

o6n3pcd1optOUTPUT.LOG.out

xyz generated by gcpd3 webservice:

C 3.552693 -3.677508 1.791743

C 3.627293 -3.881434 0.433936

C 2.887564 -3.068939 -0.465547

C 2.052437 -2.020587 0.051336

C 1.995895 -1.844304 1.464126

C 2.728442 -2.650444 2.307529

H 4.122092 -4.304310 2.471403

H 4.245437 -4.661225 0.001974

C 1.330165 -1.228553 -0.902533

H 1.356856 -1.075993 1.882381

H 2.669187 -2.497141 3.380986

C 1.479213 -1.533840 -2.238319

C 2.332711 -2.588611 -2.636602

H 0.948977 -0.963878 -2.994773

H 2.443942 -2.816006 -3.695925

N 3.017403 -3.336354 -1.798365

C 0.440175 -0.060110 -0.519903

C 1.230792 1.167889 -0.016249

C 0.362809 2.277732 0.646557

H 1.941567 0.801725 0.729493

C 1.248345 2.560128 -2.027281

C 3.091156 2.581146 -0.495658

C 0.928129 3.642782 0.199969

H -0.686121 2.214224 0.337487

H 0.383946 2.174933 1.734931

H 1.897998 2.856814 -2.856964

H 0.457066 1.942514 -2.461354

C 0.648039 3.803774 -1.304545

C 2.469907 3.688203 0.419265

H 3.670953 3.035779 -1.305308

H 3.777784 1.934834 0.056648

H 0.450294 4.450395 0.765811

H 1.097641 4.732091 -1.675847

H -0.430857 3.874924 -1.481072

H 2.804437 4.672882 0.059508

O -0.518888 -0.430802 0.510603

H -0.606871 -1.398707 0.525841

C 2.817986 3.636946 1.889522

H 2.337397 4.423336 2.475336

C 3.616610 2.786827 2.536483

H 4.147689 1.976767 2.046137

H 3.777669 2.881201 3.606105

N 2.055133 1.729434 -1.109392

P -3.044540 -0.058031 0.831484

H -0.132326 0.221720 -1.411125

N -3.185493 -1.902795 0.253550

O -4.172045 -2.251653 -0.360157

O -2.238941 -2.615127 0.579727

N -4.942933 0.100354 0.793604

O -5.402742 -0.508489 1.757613

O -5.550743 0.756695 -0.027589

N -2.820118 0.650276 -0.895480

O -2.570001 -0.054217 -1.857535

O -2.911234 1.873668 -0.874452

o6n3pcd2optOUTPUT.LOG.out

xyz generated by gcpd3 webservice:

C -0.873632 2.983676 2.278392

C -1.576601 2.020994 1.592452

C -0.965023 1.289154 0.542758

C 0.408257 1.555812 0.199349

C 1.093601 2.569516 0.929337

C 0.469470 3.261068 1.941837

H -1.355830 3.534842 3.079775

H -2.607539 1.808969 1.844625

C 1.000044 0.786947 -0.854679

H 2.114478 2.796553 0.658199

H 1.013070 4.028346 2.484283

C 0.206645 -0.155517 -1.482884

C -1.124216 -0.357624 -1.085267

H 0.603110 -0.756613 -2.295274

H -1.733577 -1.108460 -1.578621

N -1.698935 0.332657 -0.113815

C 2.453753 0.877843 -1.329448

C 3.370967 -0.128205 -0.584944

C 3.868863 0.311424 0.822001

H 2.761841 -1.030785 -0.469222

C 5.596045 0.456426 -1.425583

C 5.030804 -1.814917 -0.899557

C 5.322698 -0.184930 0.978281

H 3.857806 1.400065 0.925806

H 3.222690 -0.102255 1.601848

H 6.351288 0.130080 -2.148253

H 5.188780 1.403765 -1.779626

C 6.208150 0.609953 0.000579

C 5.419476 -1.694257 0.610486

H 5.904965 -2.091884 -1.497140

H 4.280201 -2.592702 -1.063889

H 5.660342 -0.033618 2.009884

H 7.237255 0.232748 0.035047

H 6.243310 1.664253 0.297269

H 6.476832 -1.976034 0.727052

O 3.012089 2.188182 -1.258249

H 2.532967 2.753378 -1.878910

C 4.644934 -2.548968 1.588192

H 4.937562 -2.389810 2.628172

C 3.689307 -3.447743 1.347780

H 3.334073 -3.687854 0.350306

H 3.224229 -3.996083 2.161211

N 4.507338 -0.544990 -1.440259

P -4.104278 0.051158 0.363230

N -3.508505 -1.712302 0.688696

O -3.152542 -2.496750 -0.170234

O -3.530575 -1.907030 1.900870

H 2.463625 0.535503 -2.372889

N -4.159106 0.225998 -1.535290

O -4.067476 1.390092 -1.900151

O -4.286331 -0.757473 -2.246210

N -5.946225 -0.578660 0.271717

O -6.276982 -1.697578 0.625709

O -6.694497 0.318983 -0.111726
