# Supplementary material for: Plausible Pnicogen Bonding of epi-Cinchonidine as a Chiral Scaffold in Catalysis
Source: Front Chem. 2021 Jul 6;9:669515. doi: 10.3389/fchem.2021.669515 (PMC8290064; doi:10.3389/fchem.2021.669515)
Supplement: Supplementary file 2 [file DataSheet1.docx]

Plausible Pnicogen Bonding of *epi*-Cinchonidine as a Chiral Scaffold in Catalysis

Zakir Ullah^1,2, ǂ^, Kang Kim^1,ǂ^, Arramshetti Venkanna^1,ǂ^, Hye su Kim^3^, Moon Il Kim^3^ and Mi-hyun Kim^1*^

^1^Gachon Institute of Pharmaceutical Science & Department of Pharmacy, College of Pharmacy, Gachon University, 191 Hambakmoeiro, Yeonsu-gu, Incheon, Republic of Korea, ^2^Department of Chemistry, Korea Advanced Institute of Science and Technology, Daehak-ro 291, Yuseong-gu, Daejeon, Republic of Korea, ^3^Department of BioNano Technology, Gachon University, Seongnam, Gyeonggi 13120, Republic of Korea

^*^Author for correspondence E-mail: Mi-hyun Kim: [kmh0515@gachon.ac.kr](mailto:kmh0515@gachon.ac.kr)

^ǂ^ The authors are co-first authors


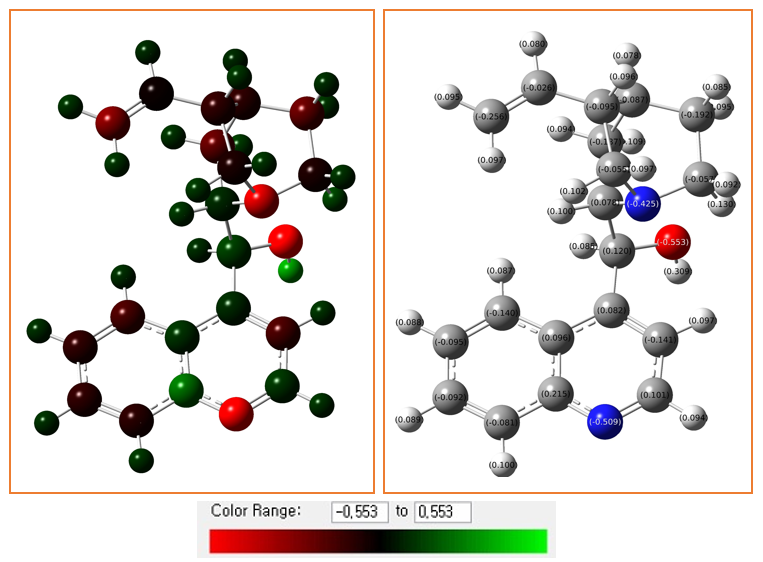


**FigureS1.** Mulliken Charge Analysis of *epi-CD*


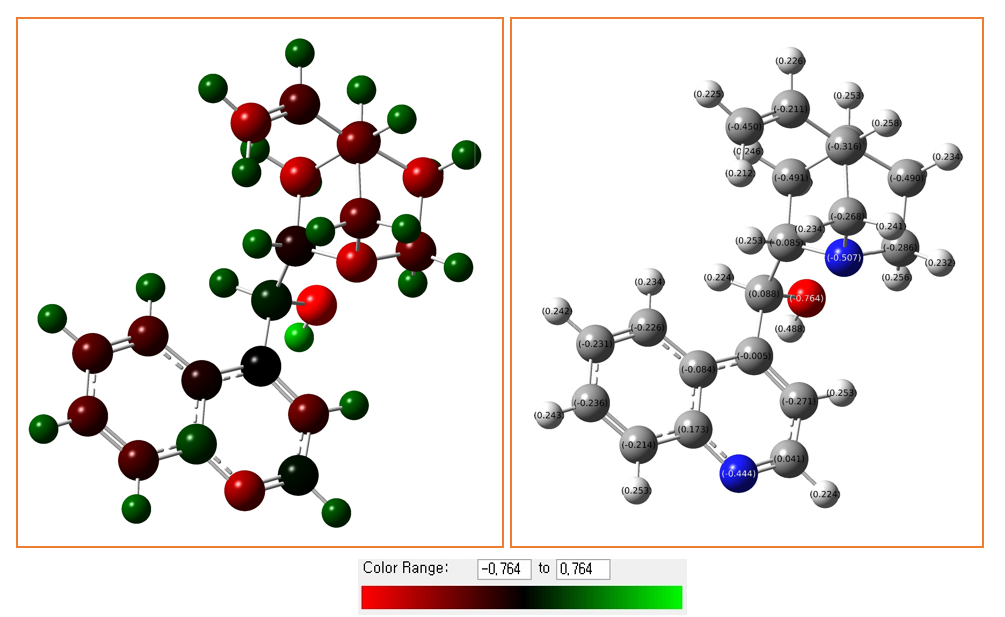


**FigureS2.** NBO Charge Analysis of *epi-CD*

**TableS1:** Mulliken Charge Analysis of *epi-CD*

| **Atoms** | **B3LYP/6-31G(d, p)** | **Atoms** | **B3LYP/6-31G (d, p)** | **Atoms** | **B3LYP/6-31G(d, p)** |
| --- | --- | --- | --- | --- | --- |
| 1 | -0.09 | 17 | 0.12 | 33 | 0.08 |
| 2 | -0.08 | 18 | 0.07 | 34 | 0.09 |
| 3 | 0.21 | 19 | -0.18 | 35 | 0.09 |
| 4 | 0.09 | 20 | 0.10 | **36** | **-0.55** |
| 5 | -0.14 | 21 | -0.05 | 37 | 0.30 |
| 6 | -0.09 | 22 | -0.05 | 38 | -0.02 |
| 7 | 0.08 | 23 | -0.08 | 39 | 0.08 |
| 8 | 0.10 | 24 | 0.10 | **40** | **-0.25** |
| 9 | 0.08 | 25 | 0.09 | 41 | 0.09 |
| 10 | 0.08 | 26 | 0.09 | 42 | 0.09 |
| 11 | 0.08 | 27 | 0.13 | **43** | **-0.42** |
| 12 | -0.14 | 28 | -0.19 | 44 | 0.08 |
| 13 | 0.10 | 29 | -0.09 |  |  |
| 14 | 0.09 | 30 | 0.09 |  |  |
| 15 | 0.09 | 31 | 0.10 |  |  |
| **16** | **-0.50** | 32 | 0.07 |  |  |

**TableS2:** NBO Charge Analysis of *epi-CD*

| **Atoms** | **B3LYP/6-31G (d, p)** | **Atoms** | **B3LYP/6-31G (d, p)** | **Atoms** | | **B3LYP/6-31G (d, p)** |
| --- | --- | --- | --- | --- | --- | --- |
| 1 | -0.23 | 17 | 0.08 | 33 | 0.23 | |
| 2 | -0.21 | 18 | -0.08 | 34 | 0.24 | |
| 3 | 0.17 | 19 | -0.04 | 35 | 0.25 | |
| 4 | -0.08 | 20 | 0.25 | **36** | **-0.76** | |
| 5 | -0.22 | 21 | -0.28 | 37 | 0.48 | |
| 6 | -0.23 | 22 | -0.20 | 38 | -0.21 | |
| 7 | 0.24 | 23 | -0.24 | 39 | 0.22 | |
| 8 | 0.25 | 24 | 0.24 | **40** | **-0.45** | |
| 9 | -0.00 | 25 | 0.24 | 41 | 0.21 | |
| 10 | 0.23 | 26 | 0.23 | 42 | 0.22 | |
| 11 | 0.24 | 27 | 0.25 | **43** | **-0.50** | |
| 12 | -0.27 | 28 | -0.49 | 44 | 0.22 | |
| 13 | 0.04 | 29 | -0.31 |  |  | |
| 14 | 0.25 | 30 | 0.24 |  |  | |
| 15 | 0.22 | 31 | 0.23 |  |  | |
| **16** | **-0.44** | 32 | 0.25 |  |  | |

**FigureS3.** Experimental ^1^H-NMR spectrum of epi-CD N-alkylation

 **FigureS4.**Simulated IR Spectra of *epi-CD*


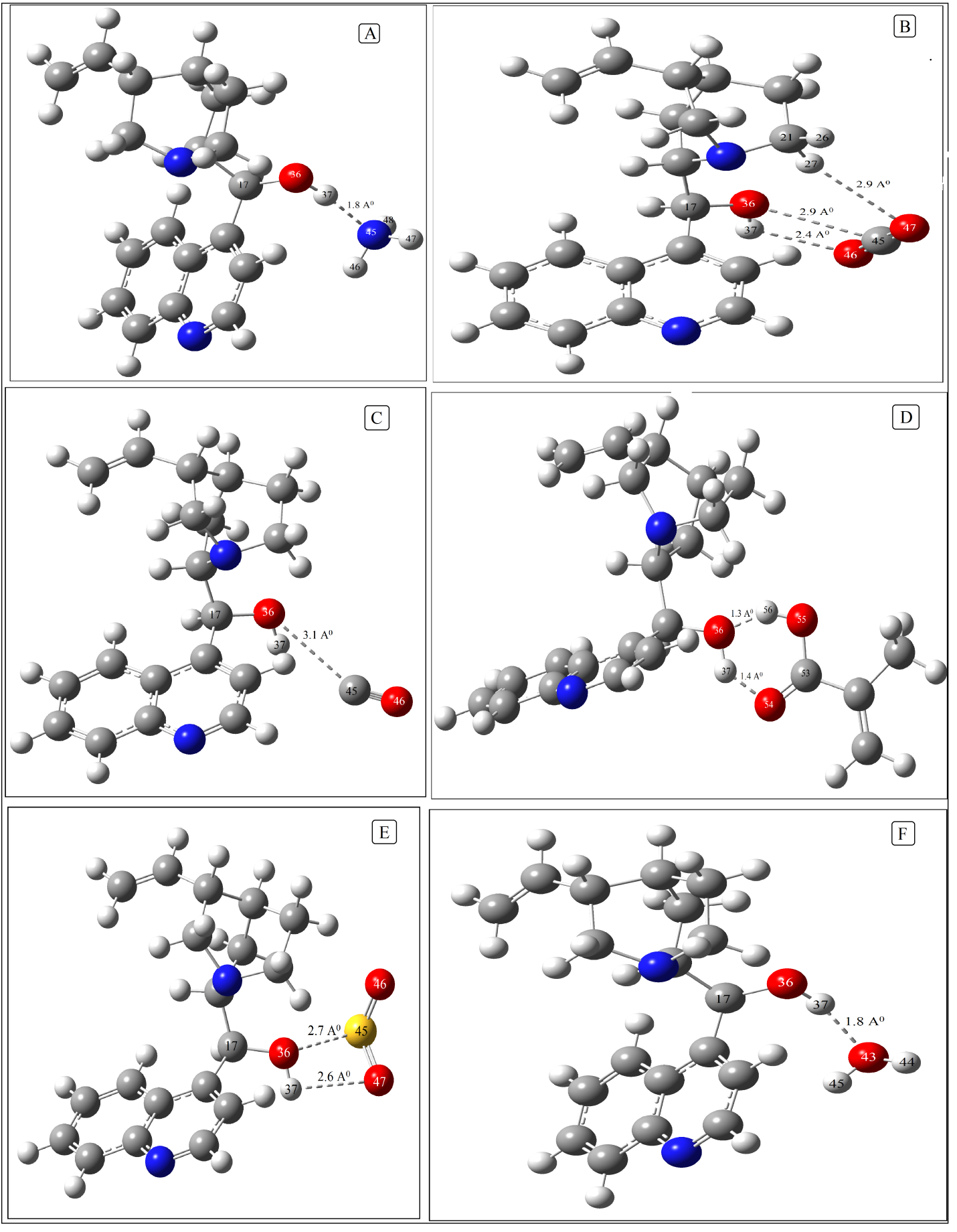


**FigureS5.** Optimized Geometry of *epi-CD* and epi-*CD*– X Complex with hydrogen bonding

**Table S3:** Optimized geometric parameters, ΔEint, ΔE_int, CP_, QNBO and QMulliken of *epi-CD-X*

(X=NH3, CO2, H2O, CO, MA, and CO): Atomic labels are with reference to Fig 6

| **Species** | **˂C_17_O_36_H_37_** | **ΔEint** | **ΔE_int_,_CP_** | **ΔEgCP-D3** | **Q_NBO_** | **Q_Mulliken_** |
| --- | --- | --- | --- | --- | --- | --- |
| epi-CD | 107.6 |  |  |  | 0 | 0 |
| epi-CD-NH_3_ | 107.6 | -11.10 | -9.28 | -11.54 | 0.007 | 0.012 |
| epi-CD-CO_2_ | 108.0 | -2.44 | -1.63 | -2.76 | 0.012 | 0.001 |
| epi-CD-CO(1) | 109.1 | -2.25 | -1.88 | -3.07 | 0.036 | 0.049 |
| epi-CD-CO(2) | 107.6 | -1.75 | -1.01 | -2.00 | -0.007 | -0.018 |
| epi-CD-MA | 108.4 | -12.86 | -9.01 | -11.79 | -0.697 | 0.000 |
| epi-CD-SO_2_ | 108.3 | -4.76 | -1.15 | -6.27 | 0.037 | 0.099 |
| epi-CD-H_2_O | 107.4 | -9.72 | -8.55 | -9.41 | -0.004 | -0.005 |


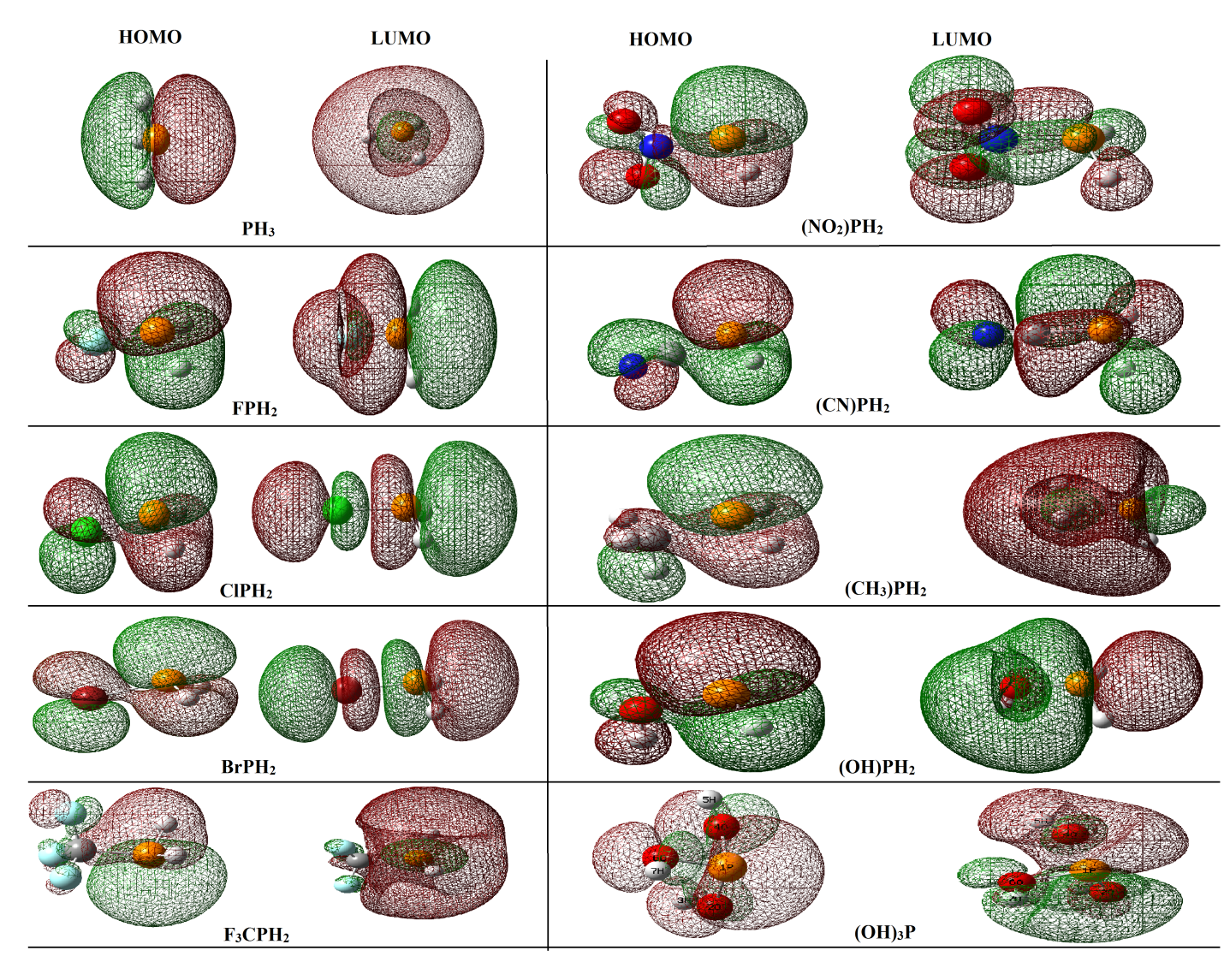


**FigureS6**. Frontier Molecular Orbitals of PH_3_ and their derivatives

**Table S4:**  SCF energy (Hartree) of epi-CD, analyte (X), and *epi-CD*-X complex

(X=PH_3_, BrPH_2_, CF_3_PH_2_, CH_3_PH_2_, ClPH_2_, FPH_2_, CNPH_2_, (OH)PH_2_, and NO_2_PH_2_)

**Table S5:** Thermochemistry of epi-CD, analyte (X), and *epi-CD*-X complex

(X=PH_3_, BrPH_2_, CF_3_PH_2_, CH_3_PH_2_, ClPH_2_, FPH_2_, CNPH_2_, (OH)PH_2_, and NO_2_PH_2_)

| Entry | X | Sum of electronic and zero-point Energies^a^ | Sum of electronic and thermal Energies^a^ | Sum of electronic and thermal Enthalpies^a^ | Sum of electronic and thermal Free Energies^a^ | Entropy Cal/Mol-Kelvin |
| --- | --- | --- | --- | --- | --- | --- |
| 1 | PH_3_ | -1264.7320 | -1264.7092 | -1264.7083 | -1264.7886 | 169.1180 |
| 2 | BrPH_2_ | -3835.8642 | -3835.8408 | -3835.8398 | -3835.9224 | 173.8010 |
| 3 | (CF_3_)PH_2_ | -1601.7465 | -1601.7209 | -1601.7199 | -1601.8074 | 184.0010 |
| 4 | (CH_3_)PH_2_ | -1304.0192 | -1303.9951 | -1303.9942 | -1304.0787 | 177.9790 |
| 5 | ClPH_2_ | -1724.3503 | -1724.3272 | -1724.3262 | -1724.4065 | 168.8240 |
| 6 | (CN)PH_2_ | -1356.9640 | -1356.9397 | -1356.9388 | -1357.0235 | 178.3500 |
| 7 | FPH_2_ | -1363.9846 | -1363.9617 | -1363.9608 | -1364.0408 | 168.3950 |
| 8 | (OH)PH_2_ | -3835.8649 | -3835.8417 | -3835.8407 | -3835.9222 | 171.3840 |
| 9 | (NO_2_)PH_2_ | -1469.2211 | -1469.1961 | -1469.1952 | -1469.2819 | 182.5860 |
| 10 | PH_3_ | -1264.7295 | -1264.7066 | -1264.7057 | -1264.7863 | 169.6460 |
| 11 | BrPH_2_ | -3835.8725 | -3835.8491 | -3835.8481 | -3835.9304 | 173.0720 |
| 12 | (CF_3_)PH_2_ | -1601.7486 | -1601.7228 | -1601.7218 | -1601.8110 | 187.6930 |
| 13 | (CH_3_)PH_2_ | -1304.0195 | -1303.9953 | -1303.9944 | -1304.0795 | 179.0310 |
| 14 | ClPH_2_ | -1724.3490 | -1724.3255 | -1724.3246 | -1724.4079 | 175.2970 |
| 15 | (CN)PH_2_ | -1356.9681 | -1356.9440 | -1356.9431 | -1357.0267 | 175.9380 |
| 16 | FPH_2_ | -1363.9839 | -1363.9608 | -1363.9599 | -1364.0409 | 170.4200 |
| 17 | (OH)PH_2_ | -1339.9626 | -1339.9396 | -1339.9386 | -1340.0191 | 169.3760 |
| 18 | (NO_2_)PH_2_ | -1469.2282 | -1469.2036 | -1469.2026 | -1469.2878 | 179.3290 |

^a^Unit is Hartree.

**FigureS7.** Optimized Geometry of *epi-CD* -Br_2_PH Complex

**
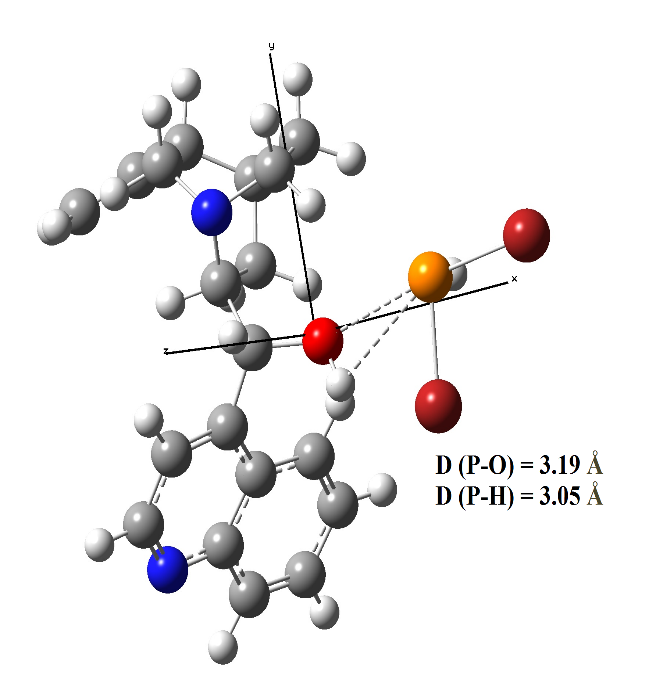

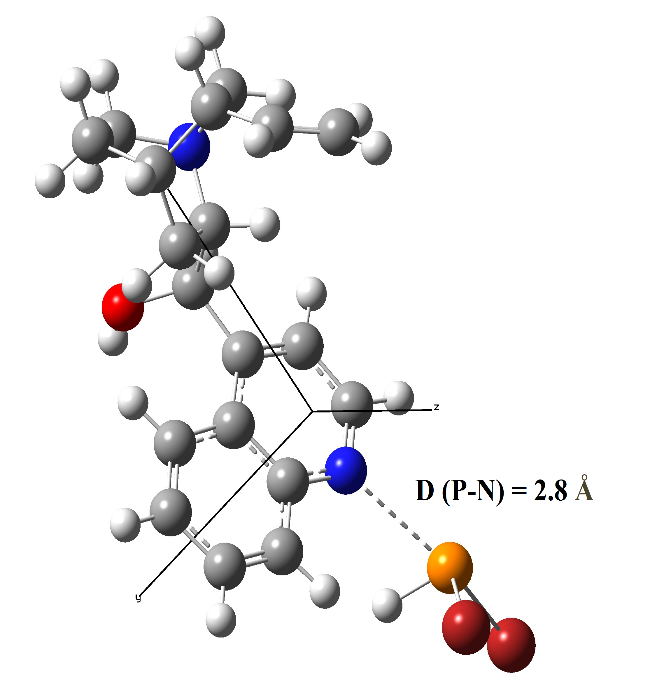
**

**FigureS8.** Optimized Geometry of *epi-CD* -Br_3_P Complex


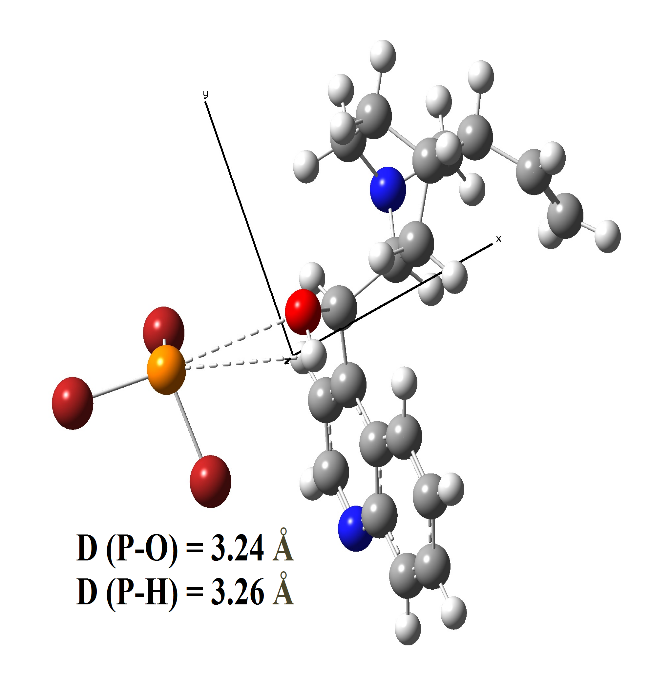

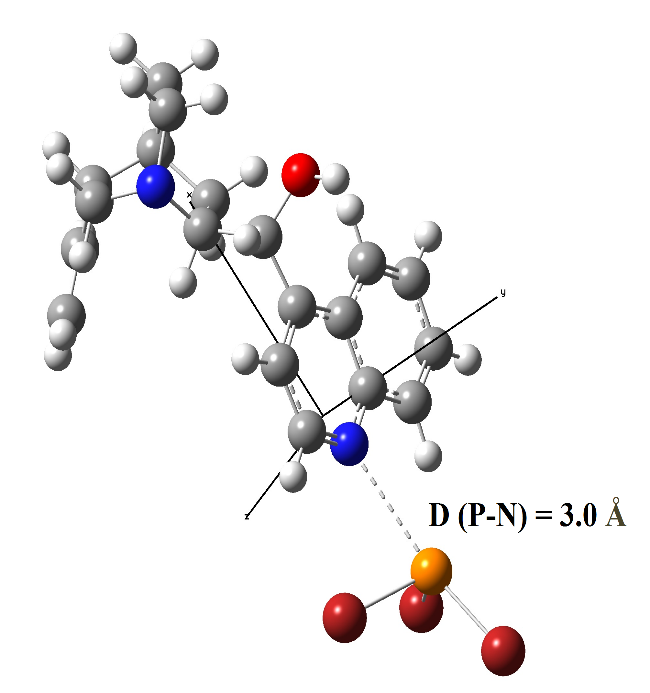


**FigureS9.** Optimized Geometry of *epi-CD-(CF_3_)2 PH* complex


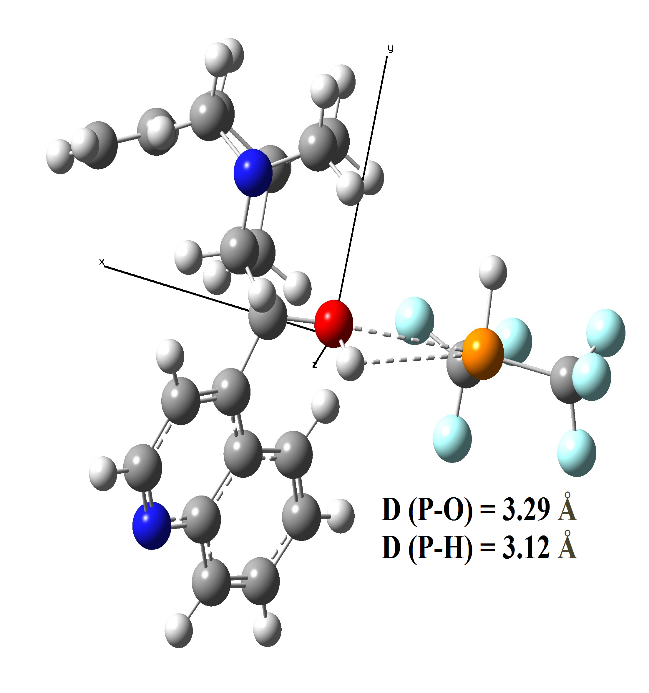

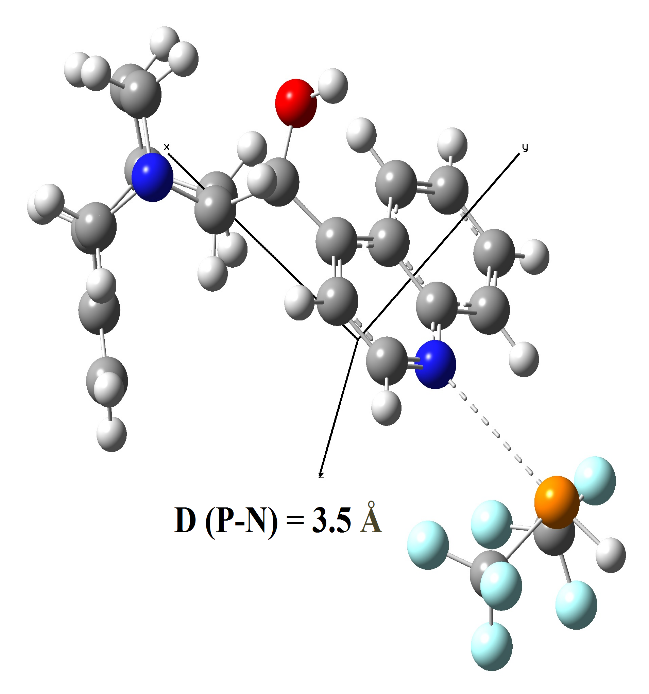


**FigureS10.** Optimized Geometry of *epi-CD-(CF_3_)3 P* complex


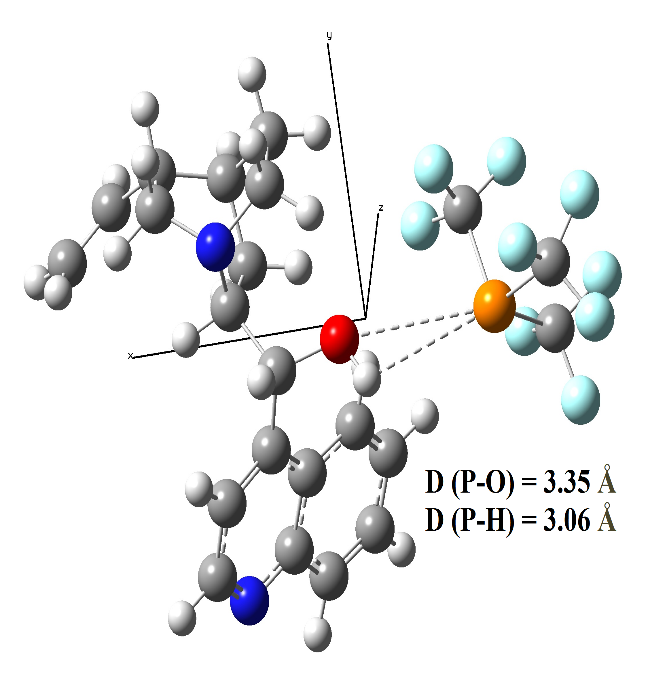

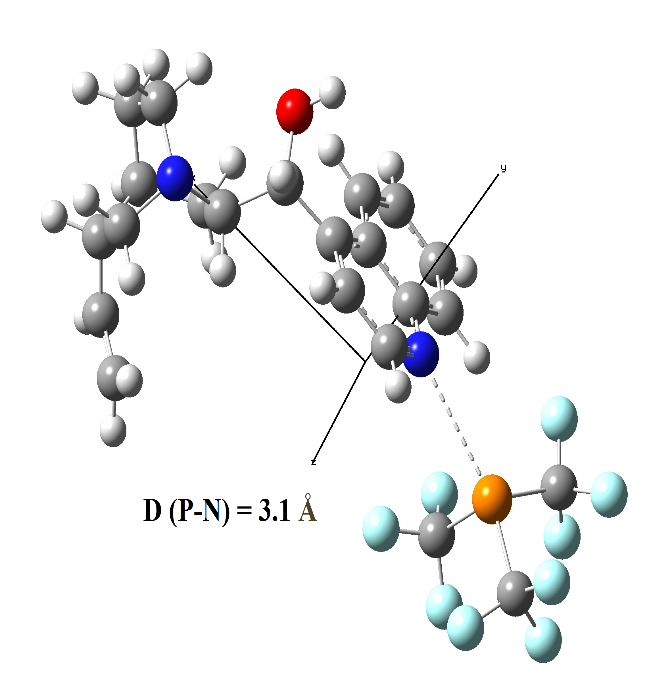


**FigureS11.** Optimized Geometry of *epi-CD-(CH_3_)_2_ PH* complex


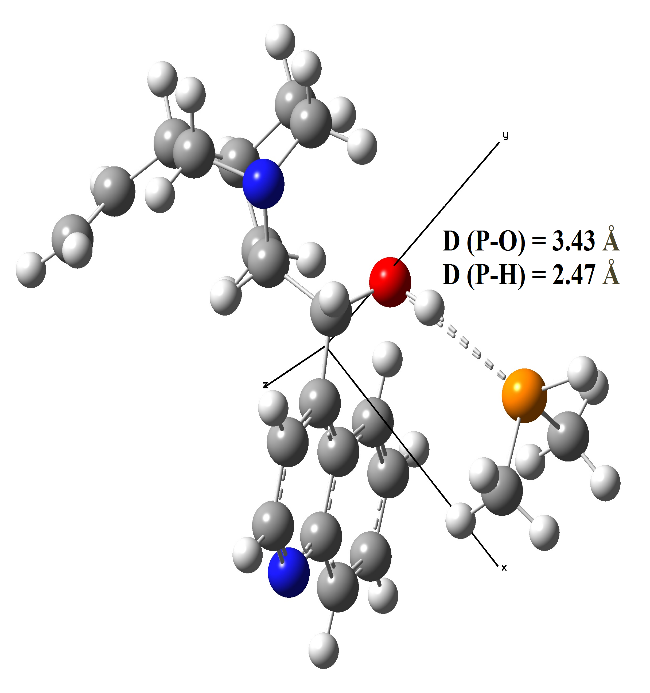

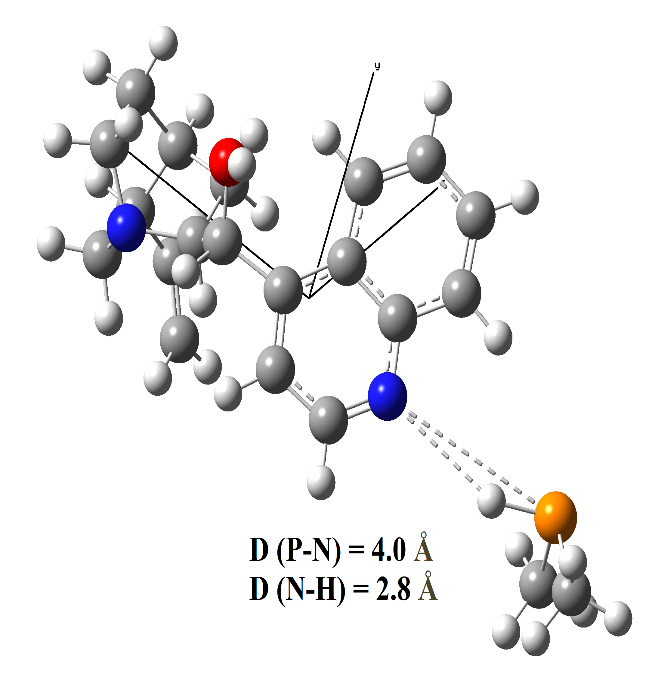


**FigureS12.** Optimized Geometry of *epi-CD-(CH_3_)_3_ P* complex


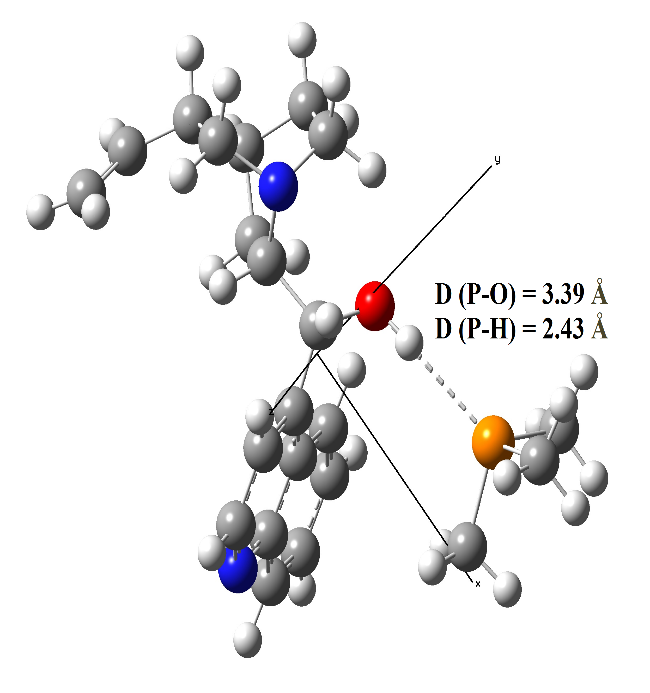

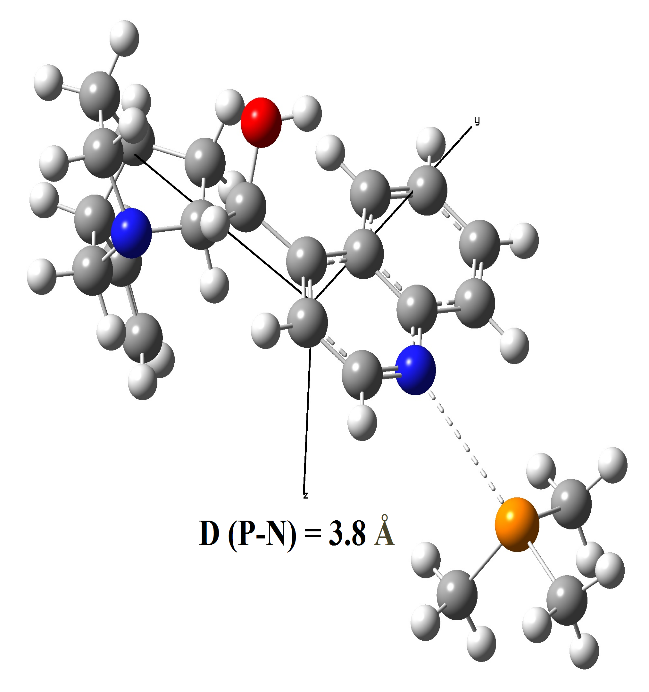


**FigureS13.** Optimized Geometry of *epi-CD-Cl_2_ PH* complex


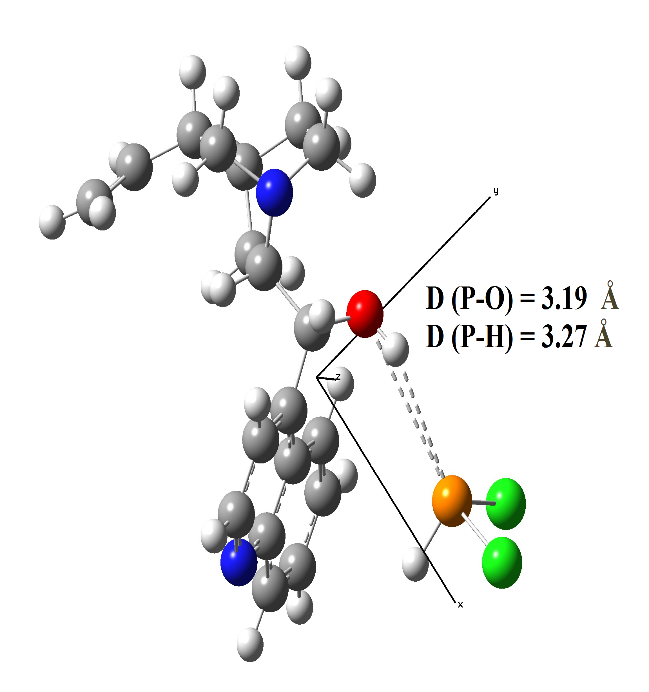

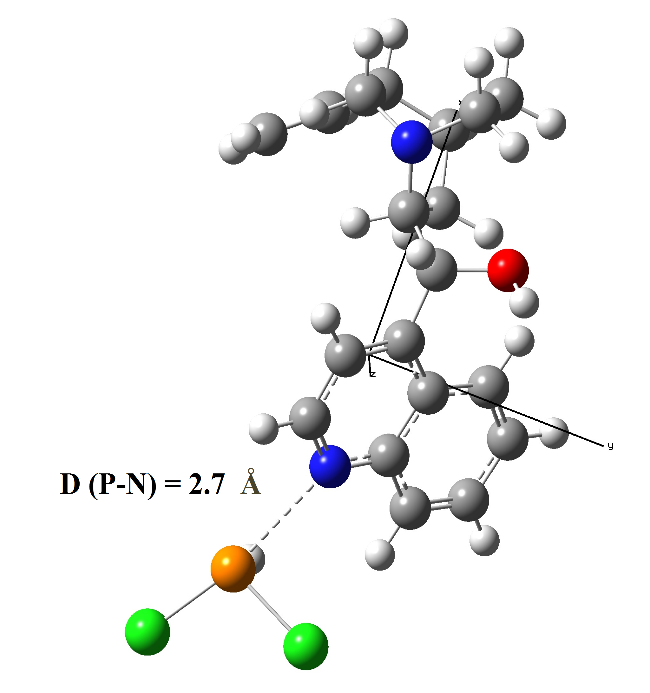


**FigureS14.** Optimized Geometry of *epi-CD-Cl_3_ P* complex


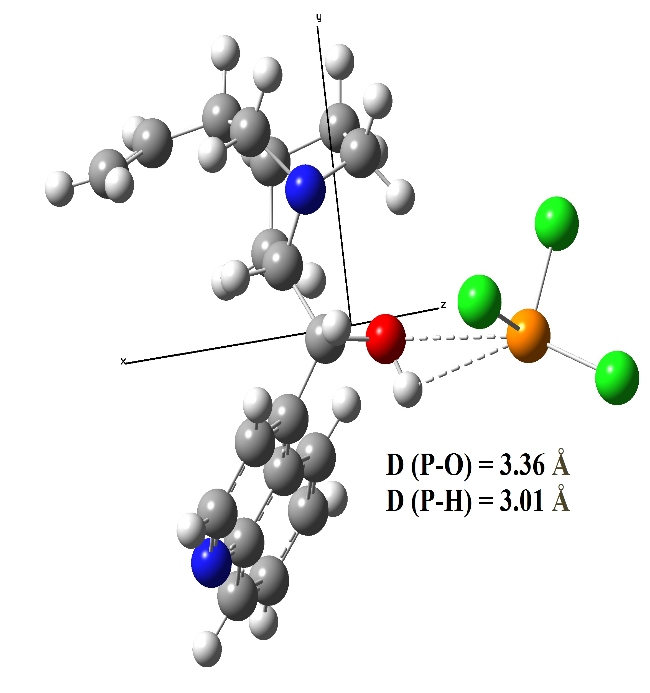

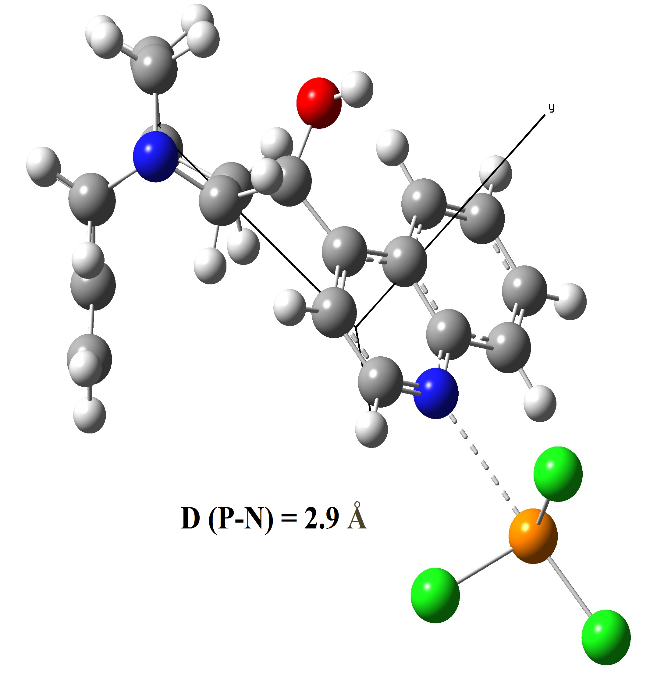


**FigureS15.** Optimized Geometry of *epi-CD-(CN)2 PH* complex

*
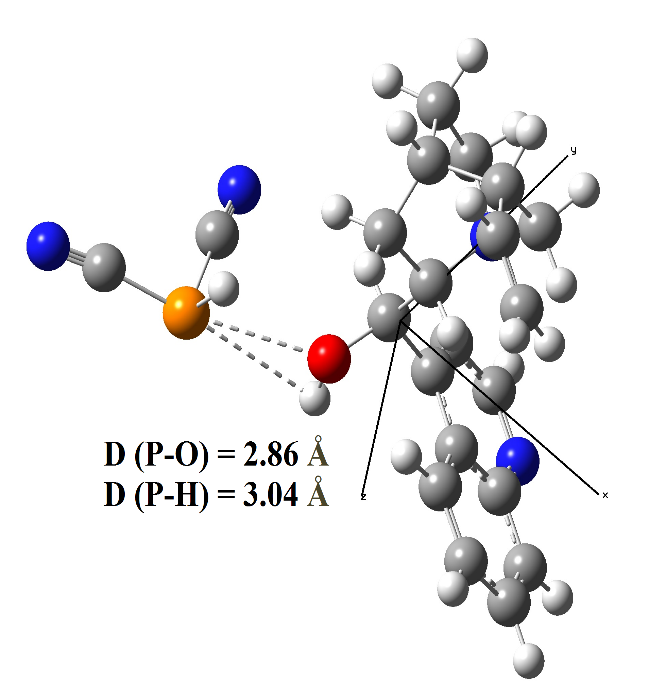

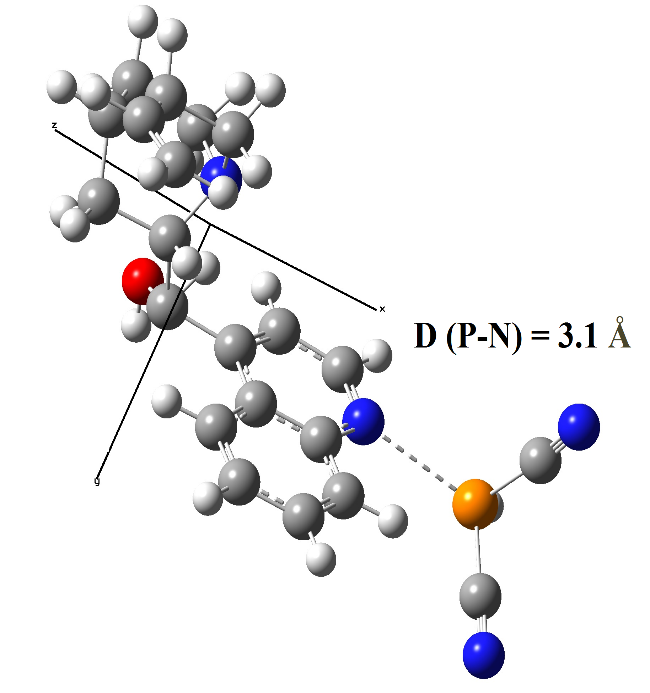
*

**FigureS16.** Optimized Geometry of *epi-CD-(CN)3 P* complex


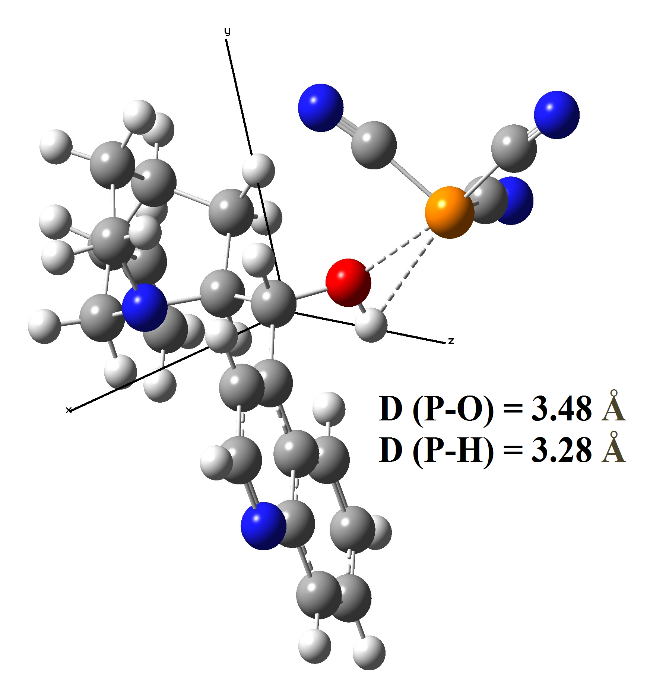

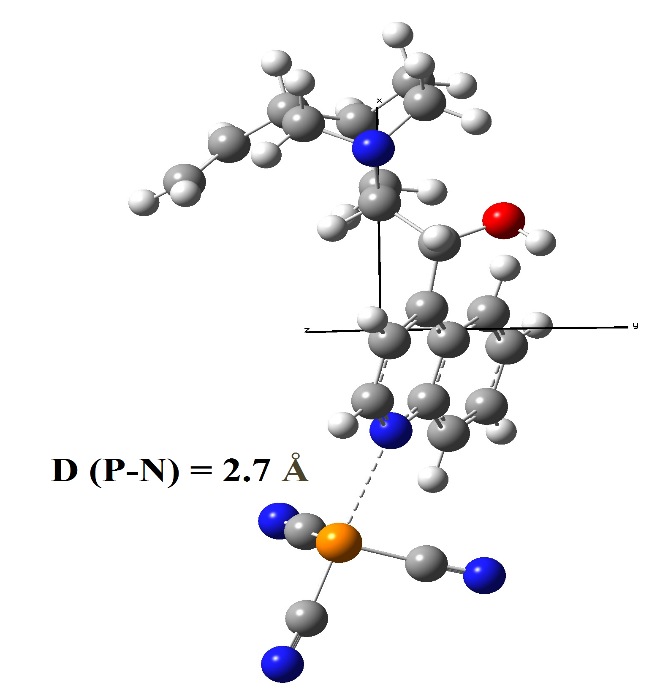


**FigureS17.** Optimized Geometry of *epi-CD-F_2_PH* Complex

*
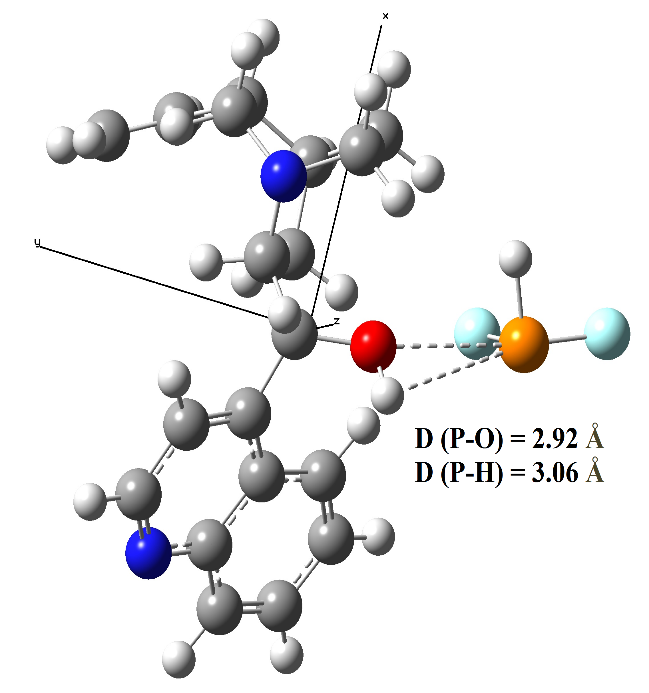

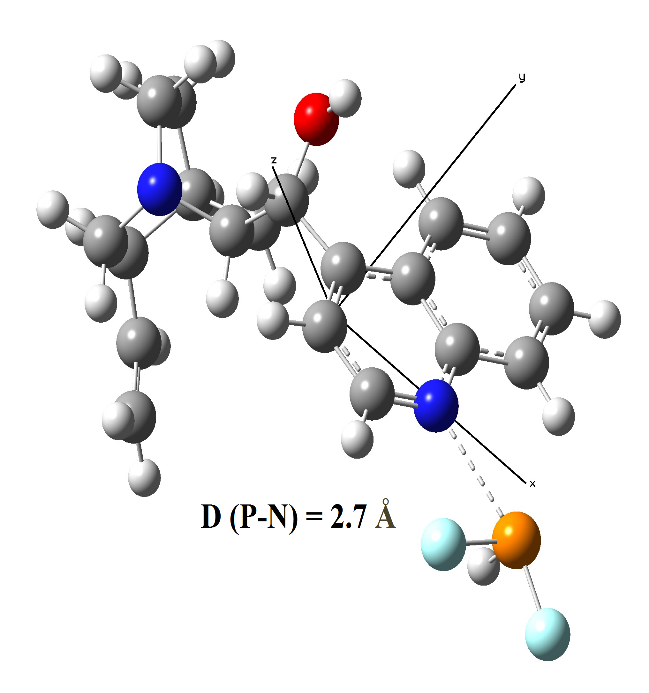
*

**FigureS18.** Optimized Geometry of *epi-CD-F_3_P* Complex


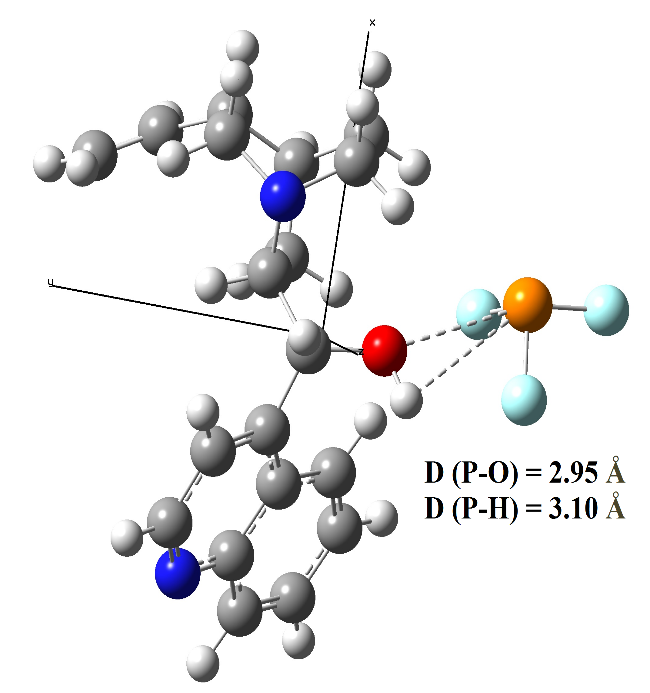

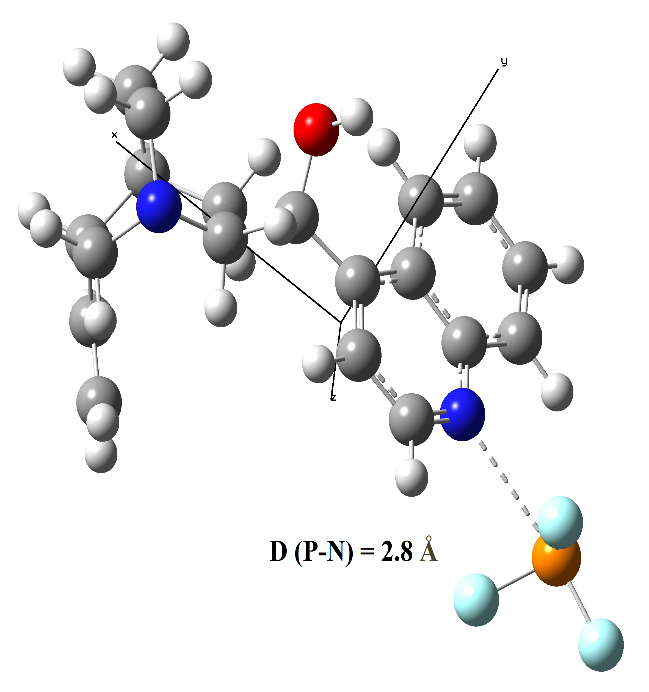


**FigureS19.** Optimized Geometry of *epi-CD -(OH)2 PH* complex

*
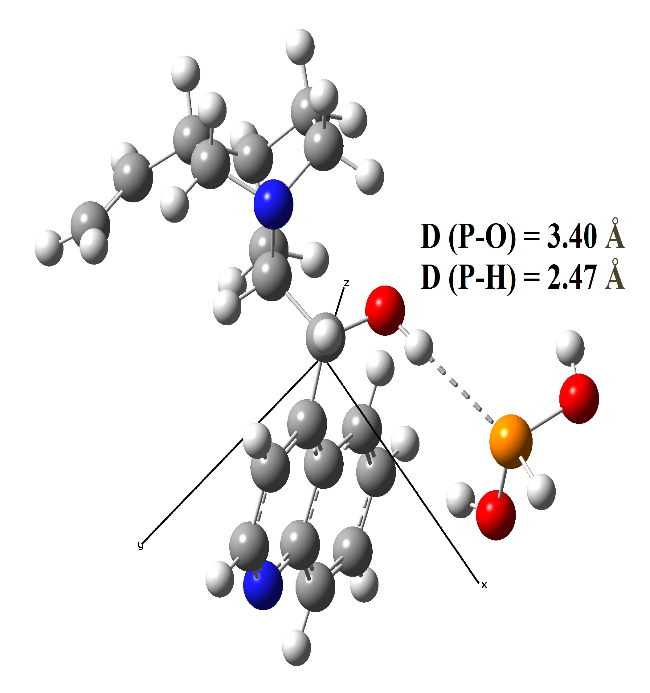

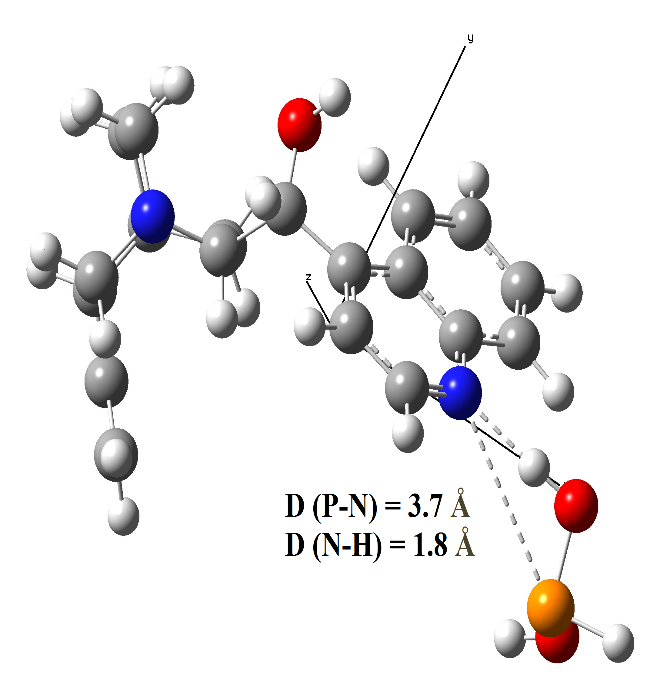
*

**FigureS20.** Optimized Geometry of *epi-CD-(OH)_3_ P* complex


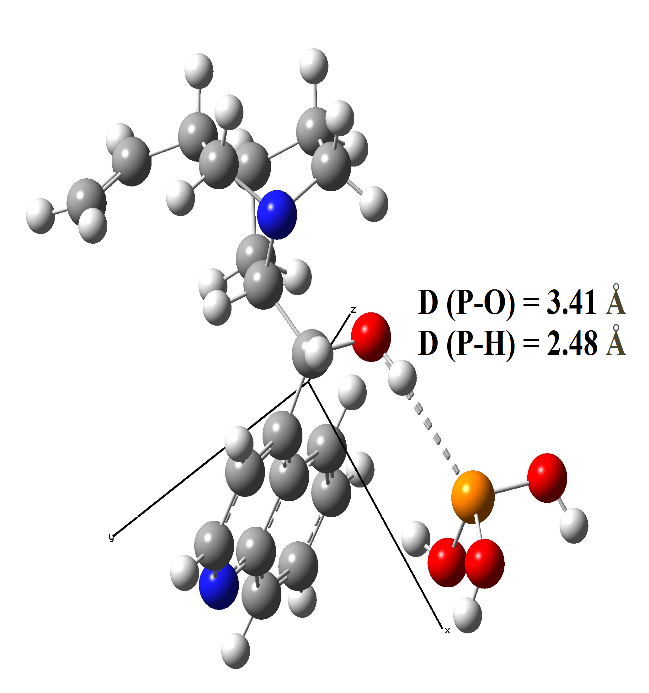

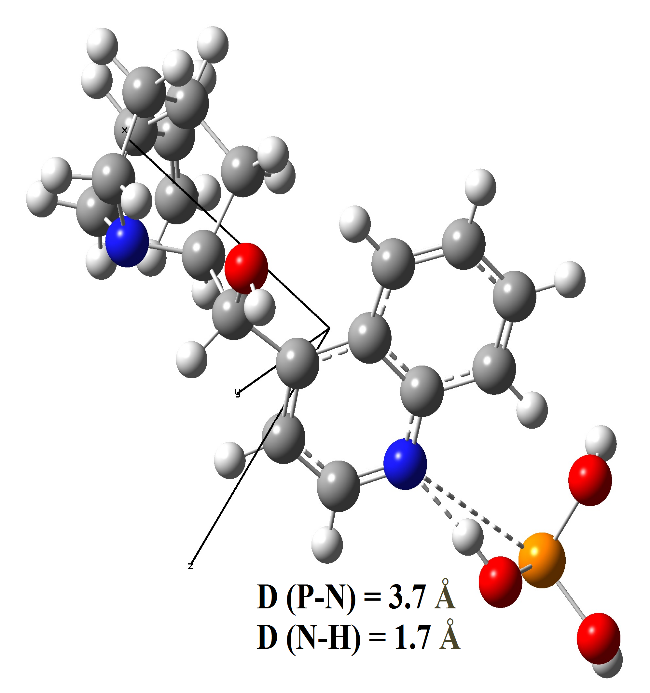


**FigureS21.** Optimized Geometry of *epi-CD-(NO_2_)2 PH* complex

*
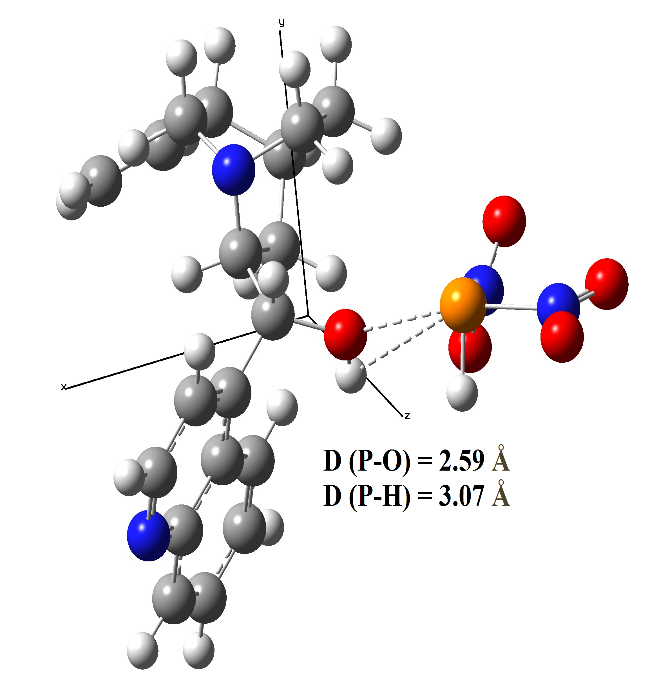

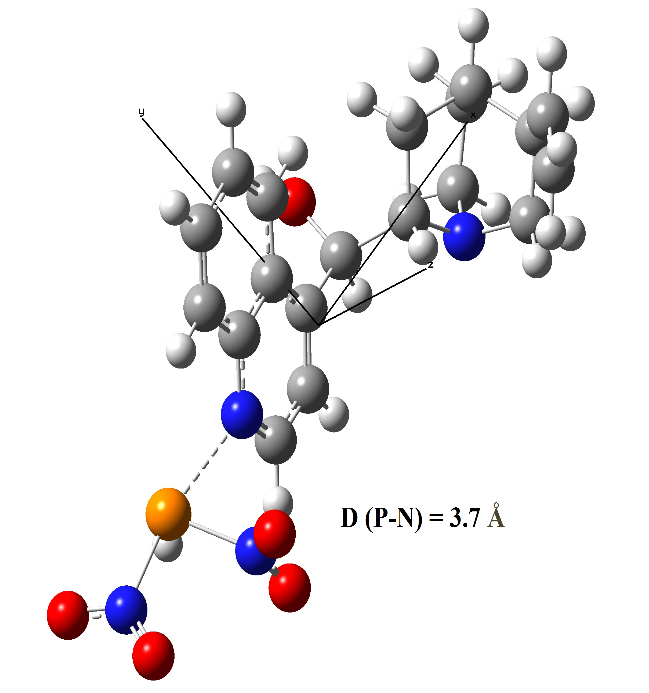
*

**FigureS22.** Optimized Geometry of *epi-CD-(NO_2_)3 P* complex


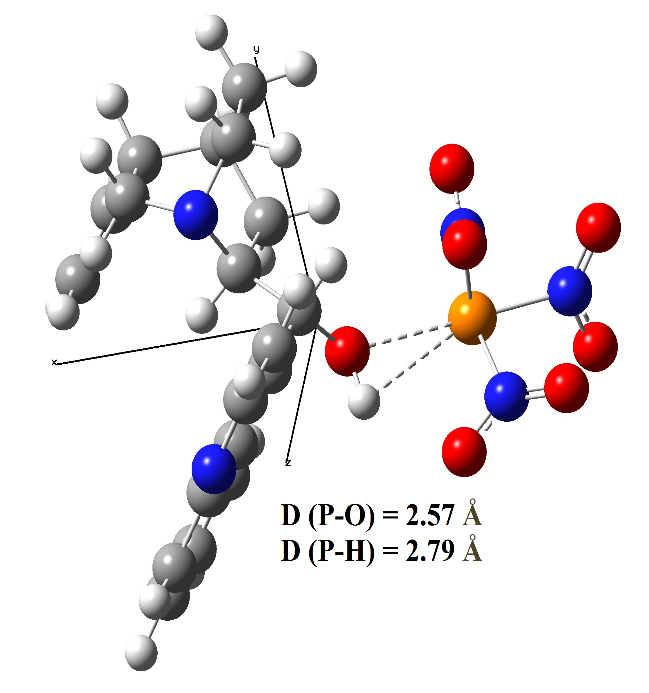

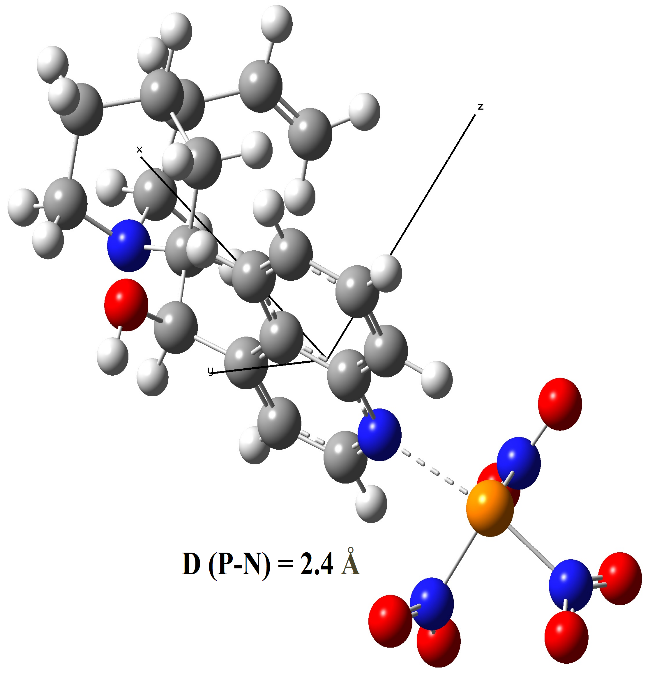

**Chem draw files for X-CD1-Complex and X-CD2-Complex**

**Representation pnicogen bonding as Chemdraw format**

**General synthetic procedure of epi-Cinchonidine:**

To stirred solution of Cinchonidine (1) (0.1 gr, 0.340 mmol) in 5 mL dry THF added PNB (0.062 g, 0.374 mmol) at 0^o^C then DEAD (0.059 mL, 0.374 mmol) was added slowly drop wise via syringe The reaction mixture was stirred at 0 °C for 30 min, then slowly allowed to room temperature and stirring was continued for 3 h. Then the reaction mixture was again cooled to 0 °C. 1 M aqueous LiOH solution (1.7 mL) followed by MeOH (0.34 mL) were added and the mixture was stirred at r.t. overnight. After completion of reaction the reaction mixture was quenched with H2O and the reaction mixture was extracted with DCM (2 X 20 mL), then the organic Phase was washed with brine, and dried over Na_2_SO_4_ and concentrated under reduced pressure to give a crude product, which was purified by silica gel column chromatography to give pure epi-Cinchonidine (**2**) in 0.078 g, 78%.

**Spectral data of *epi*-Cinchonidine**

^1^H NMR (600 MHz, CDCl_3_) δ 8.91 (d, *J* = 4.5 Hz, 1H), 8.36 (d, *J* = 8.4 Hz, 1H), 8.15 (d, *J* = 8.4 Hz, 1H), 7.72 (ddd, *J* = 8.3, 6.9, 1.2 Hz, 1H), 7.58 (ddd, *J* = 8.3, 6.9, 1.2 Hz, 1H), 7.51 (d, *J* = 4.5 Hz, 1H), 5.73 (ddd, *J* = 17.5, 10.4, 7.4 Hz, 1H), 5.12-4.93 (m, 1H), 4.98 (ddt, *J* = 21.9, 10.4, 1.3 Hz, 2H), 4.32-4.05 (bs, OH) 3.32 – 3.22 (m, 2H), 3.10 (dd, *J* = 18.2, 9.2 Hz, 1H), 2.87-2.79 (m, 2H), 2.37-2.32 (m, 1H), 1.76-1.71 (d, *J* = 2.4 Hz, 1H), 1.70 – 1.59 (m, 2H), 1.47 – 1.40 (m, 1H), 0.99 (dd, *J* = 13.6, 7.9 Hz, 1H); ^13^C NMR (150 MHz, CDCl_3_) δ 150.16, 148.61, 146.22, 141.16, 130.32, 129.08, 127.18, 126.48, 123.91, 119.76, 114.83, 70.45, 62.24, 55.73, 40.81, 39.74, 27.73, 27.23, 24.90 ppm.

**^1^H spectrum of compound-2 in CDCl_3_**

**^13^C spectrum of compound-2 in CDCl_3_**
